# Large‐scale bidirectional arrayed genetic screens identify *OXR1* and *EMC4* as modifiers of αSynuclein aggregation

**DOI:** 10.1002/2211-5463.70233

**Published:** 2026-03-30

**Authors:** Sandesh Neupane, Lea Nikolić, Lorenzo Maraio, Thomas Goiran, Nathan Karpilovsky, Stefano Sellitto, Vangelis Bouris, Jiang‐An Yin, Ronald Melki, Edward A Fon, Adriano Aguzzi, Elena De Cecco

**Affiliations:** ^1^ Institute for the Science of the Aging Brain St. Gallen Switzerland; ^2^ Institute of Neuropathology University of Zurich Switzerland; ^3^ Laboratory of Prion Biology, Department of Neuroscience, Scuola Internazionale Superiore di Studi Avanzati (SISSA) Trieste Italy; ^4^ Neurodegenerative Diseases Research Group, Department of Neurology and Neurosurgery, Montreal Neurological Institute‐Hospital McGill University Canada; ^5^ Department of Biology University of Padova Italy; ^6^ Aging and Regeneration Center, School of Basic Medical Sciences Tsinghua University Beijing P. R. China; ^7^ Institut François Jacob (MIRCen) and CNRS Fontenay‐aux‐Roses France

**Keywords:** autophagy‐lysosomal pathway, CRISPR screening, mitochondrial dysfunction, neurodegeneration, synucleinopathies, αSynuclein aggregation

## Abstract

In Parkinson's disease and other synucleinopathies, αSynuclein (αSyn) misfolds and forms Ser^129^‐phosphorylated aggregates (pSyn^129^) with the factors controlling this process largely unknown. Here, we used arrayed CRISPR‐mediated gene activation and ablation to discover new pSyn^129^ modulators. Using quadruple‐guide RNAs (qgRNAs) and Cas9, or an inactive Cas9 fused to a synthetic transactivator, we ablated 2304 and activated 2428 human genes related to mitochondria, trafficking, and motility functions in HEK293 cells. After exposure of cells to αSyn fibrils, pSyn^129^ signals were recorded by high‐throughput fluorescence microscopy and aggregates were identified by image analysis. We found that pSyn^129^ was increased by activating the mitochondrial protein *OXR1,* which decreased ATP levels and altered the mitochondrial membrane potential. Instead, pSyn^129^ was reduced by ablation of the endoplasmic reticulum (ER)‐associated protein *EMC4*, which enhanced ER‐driven autophagic flux and lysosomal clearance. OXR1 activation preferentially modulated cellular reactions to fibrils derived from multiple system atrophy (MSA) patients, whereas EMC4 ablation broadly reduced pSyn^129^ across diverse αSyn polymorphs. These findings were confirmed in human iPSC‐derived cortical and dopaminergic neurons, where OXR1 preferentially promoted somatic aggregation and EMC4 reduced both somatic and neuritic aggregates. These results uncover previously unrecognized roles for OXR1 and EMC4 in αSyn aggregation, thereby broadening our mechanistic understanding of synucleinopathies.

## Graphical abstract

Activation of the mitochondrial protein OXR1 increases pSyn129 αSynuclein aggregation by lowering ATP levels and altering mitochondrial membrane potential, particularly in response to MSA‐derived fibrils. In contrast, ablation of the ER protein EMC4 enhances autophagic flux and lysosomal clearance, broadly reducing α‐synuclein aggregates. These findings reveal new mitochondrial and ER pathways regulating α‐synuclein pathology.

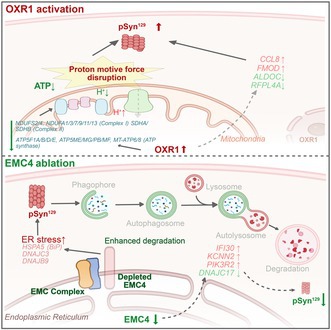

**Abbreviations**
BafA1: bafilomycin A1
Co‐IP: co‐immunoprecipitation
CRISPRa: CRISPR activation
CRISPRo: CRISPR knock‐out
DEG: Differentially Expressed Genes
DLB: Dementia with Lewy Bodies
EMC4: endoplasmic reticulum (ER)‐associated protein
ER: endoplasmic reticulum.
GSEA: Gene Set Enrichment Analysis
GWAS: Genome‐wide Association Studies
LDH: Lactate Dehydrogenase
MSA: Multiple System Atrophy
NTG: non‐targeting control
ORA: Over‐representation Analysis
OXR1: Oxidation Resistance Gene 1
PD: Parkinson's disease
PFFs: pre‐formed fibrils
pSyn129: Ser129‐phosphorylated aggregates
qgRNAs: quadruple‐guide RNAs
ROS: reactive oxygen species
SSMD: Strictly Standardized Mean Difference
ThT: Thioflavin T
αSyn: αSynuclein

Parkinson's disease (PD) is the second most prevalent neurodegenerative disorder, affecting over 10 million individuals worldwide, with its incidence steadily rising due to the aging population [1, 2]. Clinically, PD is characterized by progressive motor impairments (bradykinesia, rigidity, resting tremor) and a range of non‐motor symptoms frequently including cognitive decline [3, 4]. PD belongs to a broader class of disorders collectively termed synucleinopathies, which includes multiple system atrophy (MSA) and dementia with Lewy bodies (DLB) [5, 6, 7]. A unifying histopathological hallmark of these conditions is the accumulation of misfolded αSynuclein (αSyn) aggregates within neuronal and glial cells, giving rise to the formation of Lewy bodies, Lewy neurites, and glial cytoplasmic inclusions [8].

αSyn, encoded by the *SNCA* gene, is a small presynaptic protein that modulates synaptic vesicle trafficking and neurotransmitter release [9]. Under pathological conditions, αSyn aggregates into oligomers and protofibrils [10, 11], eventually forming amyloid inclusions through nucleated polymerization in a prion‐like fashion [12, 13, 14, 15]. More than 90% of αSyn aggregates in postmortem brains from individuals with synucleinopathies are phosphorylated at Ser^129^ (pSyn^129^) [16]. The functional role of phosphorylation in disease progression remains debated, with studies proposing both pro‐aggregatory [17] and neuroprotective effects [18, 19]. Nevertheless, its consistent association with pathological αSyn inclusions has led to its widespread adoption as a surrogate marker of αSyn aggregation in cellular and animal models [20]. These aggregates disrupt proteostasis, impair endoplasmic reticulum (ER)‐Golgi trafficking and mitochondrial function, and induce oxidative and nitrosative stress, ultimately leading to synaptic dysfunction and neuronal death [21, 22, 23].

Genetic and biochemical evidence implicates the dysfunction of mitochondria and lysosomes in idiopathic and genetic forms of PD [24, 25, 26]. 85–90% of PD cases are sporadic, with aging and exposure to environmental toxins as major risk factors [27, 28], whereas the remaining 10–15% are clustered in familial patterns. Studies of familial PD genes and genome‐wide association studies (GWAS) have highlighted polymorphisms and mutations of lysosomal/endosomal genes (*GBA1, LRRK2, TMEM175*, and *VPS35*) and mitochondrial quality‐control genes (*PINK1*, *PRKN*, *DJ‐1*), linking dysfunction in these organelles to αSyn aggregation and neurodegeneration [29]. Accumulation of misfolded proteins induces ER stress and subsequent activation of stress‐response pathways can contribute to neurodegenerative progression [30, 31]. These findings suggest that dissecting the causal links between organelle dysfunction and cellular pathology will enhance our mechanistic understanding of synucleinopathies.

Functional genomics provides a powerful toolbox to identify cellular modifiers of αSyn aggregation and propagation, and indeed siRNA and shRNA‐based screens have identified candidate regulators [32, 33, 34]. However, these approaches are limited by off‐target effects, inefficient suppression of target genes, and low specificity, which can confound the identification of crucial modifiers. Pooled CRISPR screens offer improved specificity and have provided meaningful insights into αSyn pathology [35, 36, 37, 38, 39], but are not well suited for scoring morphological phenotypes. In contrast, arrayed CRISPR screens enable high‐content phenotyping of individual gene perturbations, combining CRISPR activation (CRISPRa) and ablation (CRISPRo) to systematically map bidirectional regulatory networks [40, 41].

Here, we developed a dual CRISPRa/CRISPRo platform to target mitochondria, trafficking, and motility (MTM) genes in human cellular models. We performed an image‐based arrayed CRISPR screen to quantify pSyn^129+^ aggregates using custom libraries (T.gonfio for CRISPRa; T.spiezzo for CRISPRo), and key hits were validated in pathogenetically relevant human iPSC‐derived cortical and dopaminergic neurons. This approach identifies robust genetic modifiers of pSyn129 accumulation in a seeded αSyn aggregation model.

## Methods

### Generation and maintenance of HEK cell lines

The HEK293 QBI cell line, which stably expresses wild‐type αSynuclein (αSyn), referred to as HEK^Syn^, was used in this study and kindly provided by Prof. Kelvin C. Luk [42] (Kelvin C. Luk laboratory, University of Pennsylvania). Cells were maintained in DMEM (Catalog #31053‐036; Thermo Fisher Scientific, Waltham, MA, USA) supplemented with 10% FBS (Hyclone, Heat Inactivated, Catalog #SV30160.03HI; GE Healthcare BioSciences, Austria GmbH), 1% GlutaMax (Catalog #35050‐038; Thermo Fisher Scientific), and 1% Penicillin/Streptomycin (Catalog #15070063; Thermo Fisher Scientific). To ensure the continuous expression of αSyn, the culture medium was supplemented with Geneticin (0.4 mg·mL^−1^, Catalog #10131035; Thermo Fisher Scientific). Routine culture and expansion were performed in T75 flasks (TPP, Trasadingen, Switzerland) under standard conditions.

### Generation of CRISPR activation and CRISPR ablation cell lines

To establish CRISPR‐compatible cell lines, HEK293 QBI αSyn (referred to as HEK^Syn^) cells were transfected with dCas9‐VPR or Cas9. The dCas9‐VPR plasmid (pXPR_120, Catalog #96917; Addgene, Watertown, MA, USA) was introduced using Lipofectamine 2000 (Catalog #11668027; Thermo Fisher Scientific), following the manufacturer's protocol. After transfection, cells were subjected to antibiotic selection with Blasticidin S HCl (10 μg·mL^−1^, Catalog #A1113903; Thermo Fisher Scientific) to establish a stable dCas9‐expressing cell population.

Similarly, Cas9‐expressing cells were generated via lentiviral transduction using lentiCas9‐Blast (Catalog #52962; Addgene), followed by Blasticidin selection. To maintain the genetic modifications, the culture medium for dCas9 and Cas9‐expressing cells was supplemented with Blasticidin (10 μg·mL^−1^) alongside Geneticin (0.4 mg·mL^−1^).

For monoclonal cell line generation, polyclonal CRISPR cell lines underwent limiting dilution in 96‐well flat‐bottom plates (Catalog #7000209, TPP92096; TPP) to isolate single‐cell clones. Wells containing a single viable cell were monitored for colony formation, and successfully expanded clones were validated for Cas9 or dCas9‐VPR expression via molecular and phenotypic assays. The Cas9‐expressing Cl‐7‐Cas9‐aSyn HEK and dCas9‐VPR‐expressing Mo‐4‐dCas9‐aSyn HEK clones were designated as the CRISPRo and CRISPRa cell lines, respectively, in accordance with the experimental workflow.

Cells were passaged at 80–90% confluency using 0.25% Trypsin‐EDTA (Catalog #25200056; Thermo Fisher Scientific) and cryopreserved in Bambanker™ (Catalog #BB01; LuBio Science, Zurich, Switzerland). Cryovials were gradually cooled to −80°C before long‐term storage in liquid nitrogen. Mycoplasma testing was routinely conducted using the LookOut® Mycoplasma PCR Detection Kit (Catalog #MP0035; Sigma‐Aldrich, Darmstadt, Germany) according to the manufacturer's instructions.

### Transfection efficiency

Transfections were performed using ViaFect Transfection Reagent (Catalog #E4981; Promega), which is optimized for HEK cells. Plasmid DNA encoding BFP (T.gonfio) or a GFP plasmid of similar size (Addgene Plasmid #48138) was mixed with ViaFect in Opti‐MEM™ (Catalog # 31985062; Thermo Fisher) following the manufacturer's instructions. Forty‐eight hours post‐transfection, efficiency was evaluated by quantifying the percentage of GFP‐ or BFP‐positive cells using fluorescence microscopy and flow cytometry.

### Expression and purification of recombinant αSyn

Human wt full‐length αSyn (NM_000345) was cloned into the ampicillin‐resistant bacterial expression vector pRK172 and transformed into competent BL21(DE3) RIL E. coli cells. Transformed bacteria were plated on LB/Amp agar plates and incubated overnight at 37 °C. For preculture preparation, a single colony from the overnight plate was transferred to SOC medium (Catalog #15544034; Invitrogen™, Waltham, MA, USA) and incubated for 6 h at 37 °C, 200 rpm. The preculture was then used to inoculate Terrific Broth (TB) medium supplemented with ampicillin in baffled flasks, followed by overnight incubation at 37 °C with shaking to induce protein expression.

For cell harvesting, overnight cultures were transferred into centrifuge bottles, pelleted by centrifugation, and the supernatant was discarded. Cell pellets were stored at −20 °C until further processing. For αSyn purification, the pellets were thawed and lysed via freeze‐thaw cycles using liquid N_2_ and tap warm water. The lysates were cleared by centrifugation, and the supernatant was treated with streptomycin sulfate to precipitate DNA, followed by ammonium sulfate precipitation to isolate αSyn. The pellet was resuspended and dialyzed against ion exchange chromatography (IEC) start buffer for desalting.

Purification was performed in two steps: anion exchange chromatography (AEC) for initial purification and size exclusion chromatography (SEC) to achieve high purity of the monomeric αSyn. The purified αSyn was concentrated to ~ 700 μm (~ 10 mg·mL^−1^) using Amicon Ultra centrifugal filter devices (10 kDa MWCO, Catalog #UFC503024; Millipore, Darmstadt, Germany). The protein was aliquoted into 250 μL volumes and stored at −20°C. All steps were performed at room temperature following BSL‐2 biosafety guidelines.

### Preparation and sonication of αSyn pre‐formed fibrils

Purified monomeric αSyn was diluted to 345 μm (4.98 mg·mL^−1^) in PBS (300 μL/aliquot) and incubated in screw‐cap tubes at 60 °C with continuous agitation (1000 rpm, 72 h) in a Thermomixer with a heated lid to induce fibrillation. Fibrillation progression was monitored at 24, 48, and 72 h by centrifuging 20 μL samples (16 000 ***g***, 60 min, 25 °C), separating supernatants and pellets for storage at −20 °C. Supernatants and pellets were analyzed via SDS/PAGE (12% Bis‐Tris gel, MOPS buffer) after denaturation (95 °C, 10 min) and Coomassie staining (Instant Blue) to quantify αSyn. Aliquoted pre‐formed fibrils (PFFs) were stored at −80 °C to preserve integrity. For experiments, PFFs were thawed, diluted to 0.5 mg·mL^−1^ (monomer equivalent) in PBS, and sonicated (10‐min cycles: 30 s on/30 s off) using a high‐powered water bath sonicator filled with ice‐cooled water to ensure consistent fragmentation. This standardized protocol was consistently followed to ensure reproducibility across all experimental runs.

### Characterization by transmission electron microscopy (TEM)

αSyn monomers, PFFs, and sonicated PFF ultrastructures were analyzed by TEM. Samples were diluted to 0.5–1 mg·mL^−1^ in PBS (pH 7.4), applied to mesh copper grids for 1–2 min, and excess liquid blotted with filter paper. Grids were negatively stained with 2% uranyl acetate (Electron Microscopy Sciences, Catalog number: 22400‐2) for 30 s, rinsed three times with double‐distilled water, and air‐dried at room temperature. TEM imaging was performed on a FEI Talos 120 TEM (Thermo Fisher Scientific) at the Center for Microscopy and Image Analysis (University of Zurich), using magnifications of 6000× to 120 000× to capture an overview and structural features of αSyn fibrils.

### Thioflavin T (ThT) fluorescence assay

Fibril formation was monitored using ThT (Catalog number: T3516; Sigma‐Aldrich). A 10 mm ThT stock was prepared in ultrapure water, filtered (0.22 μm syringe filter), and diluted to 25 μm in PBS. For each time point (0, 1, 3, 12, 24, 48, 72 h), 5 μL of fibril suspension was added to 195 μL ThT solution in a black‐walled 96‐well plate (Catalog number: 655209; Greiner Bio‐One, Kremsmünster, Austria). After 5 min of incubation (25 °C), fluorescence was measured using a FLUOstar Omega Microplate Reader (BMG Labtech, Offenburg, Germany) at 450 nm excitation/480 nm emission.

### Preparation of fibrillar and patient‐derived fibrils αSyn strains

The generation of the two αSyn fibrillar strains (fibrils and ribbons, 350 μm) [43], and patient‐derived strains (PD, DLB, MSA; 100 μm) [44], their fragmentation and storage at −80 °C have been extensively described. For use, aliquots were thawed in a 37 °C water bath (3 min), equilibrated to room temperature, and diluted to 0.5 mg·mL^−1^ in PBS. No refreezing was permitted to preserve fibril integrity.

### LT1‐mediated PFF delivery

Sonicated PFFs (0.5 mg·mL^−1^) were complexed with TransIT™‐LT1 Transfection Reagent (Catalog #MIR 2306; Mirus Bio, Madison, WI, USA) at a 1 : 3 (v/v) ratio in Opti‐MEM™ (Catalog number: 31985070; Thermo Fisher Scientific) and incubated for 15 min (25 °C). Cells were washed with PBS, treated with the PFF‐LT1 complex (final PFF concentration: 7.5 μg mL^−1^), and maintained in DMEM complete medium (without penicillin/streptomycin).

### CRISPR activation and ablation primary screen

The screening assay was conducted in a high‐throughput 384‐well format. CRISPRa cells (3000 cells/well) were seeded in PDL‐coated 384‐well PhenoPlates (Catalog #6057300; PerkinElmer, Waltham, MA, USA) using the BioTek MultiFlo FX Multimode Dispenser (Agilent). Transfection was performed using a custom genome‐wide CRISPRa library (T.gonfio library; 2428 genes related to mitochondria, trafficking, and motility, with four non‐overlapping guide RNAs [qgRNAs] per gene) [41]. Here, “motility” in MTM refers to cytoskeletal and motor/adaptor pathways related genes that control intracellular transport. Each well received 60 ng plasmid DNA complexed with 0.3 μL ViaFect Transfection Reagent (#E4981; Promega) in Opti‐MEM™ I Reduced Serum Medium (#31985070; Thermo Fisher Scientific). Non‐targeting (scrambled) qgRNAs and *RAB13‐*positive controls were included. Post‐transfection, cells underwent 48‐h puromycin selection (0.6 μg·mL^−1^, #A1113803; Thermo Fisher Scientific) before treatment with 7.5 μg·mL^−1^ sonicated PFFs complexed with TransIT™‐LT1 Transfection Reagent (#MIR2306; Mirus Bio). After 72 h, cells were fixed and immunostained for phosphorylated αSyn at Ser^129^ (pSyn^129^). Imaging was conducted using the GE IN Cell Analyzer 2500HS high‐content microscopy system.

For ablation screen, CRISPRo cells (2000 cells/well) were plated and transfected with a custom CRISPRo library (T.spiezzo library; 2304 genes, 4 gRNAs per gene). Although alternative transcription start sites can sometimes inflate CRISPRa libraries, in this study the difference in library size mainly reflected sublibrary arrangement, with some “tail genes” continuing mid‐plate from previous collections, ensuring complete coverage but resulting in a slightly larger CRISPRa library. Non‐targeting controls and *PIKFYVE*‐positive controls were included. The transfection and PFF treatment protocols mirrored CRISPRa, but puromycin selection lasted 96 h to ensure stable ablation. pSyn^129^ inclusion analysis followed the same immunostaining, imaging, and analysis workflows. Imaging was conducted using the ImageXpress Confocal HT.ai (Molecular Devices). Both screenings were performed in duplicate, with each sample plated in two independent wells to ensure technical reproducibility.

### iPSCs culture and maintenance

The maintenance of iPSCs, neuronal differentiation, and maturation followed the protocols described here [45] and Tian *et al*., [46]. WTC11 human iPSCs harboring a trimethoprim (TMP)‐inducible CRISPR activation system (DHFR–dCas9–VPH) were kindly provided by the Kampmann Laboratory (University of California, San Francisco). iPSCs were cultured on Matrigel‐coated (Corning® Matrigel® hESC‐Qualified Matrix, LDEV‐free, Catalog #54277; Corning, Corning, NY, USA) cell culture dishes with daily media changes in STEM Flex Medium (StemFlex™ Medium, Catalog #A3349401; Thermo Fisher Scientific). Additionally, a fresh 10 μm ROCK inhibitor (Y‐27632, Catalog #1254/10; Tocris Bioscience, Minneapolis, MN, USA) was added to the medium. Cells were dissociated using Accutase (StemPro® Accutase® Cell Dissociation Reagent, Catalog #A1110501; Thermo Fisher Scientific). For long‐term storage, cells were cryopreserved in CryoStor® (Catalog #C2874; Sigma‐Aldrich). Routine mycoplasma testing was performed to ensure culture integrity. CRISPRa guide RNAs were introduced into iPSCs using lentiviral transduction at an MOI of 0.3, followed by puromycin selection to generate CRISPRa Non‐targeting and CRISPRa OXR1 lines before differentiation.

### Differentiation and culture of iPSCs into i3 cortical neurons

The iPSCs were differentiated into glutamatergic (i3) cortical neurons in Matrigel‐coated dishes using an induction medium composed of DMEM/F‐12, HEPES (Catalog #31330095; Invitrogen), supplemented with N2 supplement (N2 Supplement, 100X, Catalog #17502‐048; Invitrogen), non‐essential amino acids (NEAA MEM, Catalog #10370047; Thermo Fisher Scientific), and GlutaMAX Supplement 100X (Gluta‐MAX MEM, Catalog #41090028; Thermo Fisher Scientific). Additionally, 2 μg·mL^−1^ doxycycline (Catalog # D9891‐10G; Sigma) was freshly added to the medium daily.

Differentiated neurons at day 3 *in vitro* (DIV 03) were further matured in a neuronal maturation medium. Culture plates were coated with Poly‐D‐Lysine (Catalog #A3890401; Thermo Fisher Scientific), Poly‐L‐Ornithine (Catalog #P4957; Sigma‐Aldrich), and mouse Laminin (Natural, Catalog #23017015; Thermo Fisher Scientific). The maturation medium consisted of BrainPhys™ Neuronal Medium (Catalog #05790; StemCell Technologies, Vancouver, BC, Canada), supplemented with recombinant human NT‐3 (Neurotrophin‐3, Catalog #450‐03; Peprotech, Waltham, MA, USA), recombinant human/murine/rat BDNF (Brain‐Derived Neurotrophic Factor, Catalog #450‐02; Peprotech), serum‐free B‐27™ Supplement 50X (Catalog #A3582801; Thermo Fisher Scientific), and 100 nm TMP (Trimethoprim, Catalog #195527; Biomedicals Europe, Darmstadt, Germany) for dCas9‐VPH activation.

At DIV04, iPSC‐derived cortical neurons were treated with sonicated pre‐formed fibrils PFFs at a concentration of 7.5 μg·mL^−1^, utilizing half‐conditioned medium to minimize medium turnover effects. The following day, the medium was replaced, and every 2–3 days, iPSC‐derived cortical neurons were replenished with TMP‐containing neuronal maturation medium for a total duration of 4 weeks. For the shRNA‐based knockdown experiment, the EMC4 human shRNA Plasmid Kit and a 29‐mer scrambled shRNA cassette (non‐targeting control) (Catalog #TL300940; OriGene, Rockville, MD, USA) were introduced using lentiviral transduction at a multiplicity of infection (MOI) of 2 on Day 4 *in vitro* (DIV 04). After 24 h, the medium was fully replaced with half‐conditioned medium with PFFs.

### Differentiation of iPSCs into iDA neurons

iPSCs were differentiated into induced dopaminergic (iDA) neurons using an adapted protocol from Sheta *et al*., [47]. Cells were plated on Matrigel‐coated dishes and maintained in induction medium based on DMEM/F‐12 with HEPES (Table S5). The differentiation medium was freshly prepared and applied, and cells were left undisturbed for the first 3 days (DIV 0–3) to facilitate initial neuronal induction. On DIV 3, differentiated iPSCs were detached and replated onto Poly‐L‐Ornithine (PLO)‐ and Laminin‐coated plates in Neurobasal‐based PreDOPA medium (Table S5) to initiate dopaminergic differentiation. Cells were maintained in PreDOPA medium for 3 days (DIV 3–6) without disturbance to allow cell attachment and early differentiation into the dopaminergic lineage. Following the PreDOPA stage (DIV 6 onward), cells were transitioned to iDA dopaminergic neuron media, which was based on Neurobasal Medium (Table S5). To maintain optimal neuronal differentiation conditions, a half‐media change was performed every 5 days.

### iDA neuronal culture and PFF treatment assay

Neurons were treated with sonicated αSyn PFFs after 5 days in iDA dopaminergic neuron media at a concentration of 7.5 μg·mL^−1^, utilizing half‐conditioned medium. The following day, the medium was replaced, and every 5 days, neurons were replenished with TMP‐containing neuronal maturation medium for a duration of 4 weeks. Imaging was performed using the Operetta CLS High‐Content Analysis System (PerkinElmer). Image analysis was conducted using the CellProfiler pipeline as previously described. To account for batch‐to‐batch variability of PFFs, data from different PFF batches were normalized to the corresponding non‐targeting control (NTG) conditions, enabling conversion into effect size for comparative analysis.

### Immunostaining

Cells were washed once with Tris‐buffered saline (TBS) and fixed with 4% paraformaldehyde (PFA, methanol‐free) (Catalog #043368.9M; Thermo Fisher Scientific) for 15 min at room temperature (RT). After three washes with TBS, cells were permeabilized with 0.1% Triton X‐100 (Catalog #9036‐19‐5; Sigma) in TBS for 10 min, followed by another three washes with TBS. To minimize nonspecific binding, cells were incubated for 1 h at RT in 4% bovine serum albumin (BSA) in TBS. For primary antibody staining, cells were incubated overnight at 4 °C with anti‐αSyn (phospho S^129^; clone 81A) [81A] (Catalog #825701; BioLegend, San Diego, CA, USA) diluted 1 : 2500 in 0.5% BSA/TBS. The following day, after three washes with TBS, cells were incubated for 1 h at RT with the secondary antibody, diluted 1 : 400 in 0.5% BSA/TBS. For whole‐cell labeling, HCS Cell Mask™ Deep Red Stain (Catalog #H32721; Thermo Fisher Scientific) was diluted 1 : 5000 in PBS, and nuclei were counterstained with DAPI (1 : 10 000 dilution, Catalog #D9542; Sigma‐Aldrich) for 10 min. All washing steps were performed using the BioTek 405 TS Washer (Agilent) to ensure consistency across experiments. Unless otherwise specified, 81A was used for pSyn^129^ immunostaining. For experiments requiring alternative validation, phospho‐αSyn (pS^129^) antibody [EP1536Y] (Catalog #ab51253; Abcam, Cambridge, UK) was employed. Additional antibodies and their specific dilutions are listed in Table S6.

### Imaging and image analysis

Image acquisition was performed using a GE IN Cell Analyzer 2500HS widefield system (10X or 20X or 40X objectives) or an ImageXpress Confocal HT.ai (Molecular Devices; 10X or 20X objective). For nuclear and pSyn^129^ signal segmentation, ilastik 1.4.0 was used, employing a random forest classifier algorithm trained manually on raw images. Probability maps generated in ilastik were exported to cellprofiler 4.2.1 for further analysis. Cell segmentation was performed using the Propagation algorithm with the nucleus as a reference, while object segmentation was achieved using the minimum cross‐entropy algorithm in cellprofiler. Quantitative assessments included total cell counts, the percentage of pSyn^129+^ cells, total pSyn^129^ spot area and total pSyn^129^ signal intensity. Raw images were used for pSyn^129^ spot intensity calculations, without applying pretrained ilastik models. For confocal 3D imaging, a FluoView FV10i Confocal Laser Scanning Microscope (Olympus, Tokyo, Japan) was used. Images were adjusted uniformly, and pseudo‐coloring was applied to generate representative images using imagej. For most experiments, each condition included at least ~ 10 000 cells across technical replicates (unless otherwise stated in the figure legends).

### Neuronal image analysis

Neuronal image analysis was conducted using CellProfiler to quantify the pSyn^129^ spots in the neurite and soma regions separately. Segmentation was applied to delineate these compartments. Feature enhancement and suppression techniques were applied to refine segmentation and associate pSyn^129^ spots with their respective neuronal compartments. The detected pSyn^129^ signal was normalized to the MAP2‐positive area to account for morphological variations. pSyn^129^ spots outside defined neurite and soma regions were excluded to maintain specificity.

### Labeling and treatment of fibrils

Pre‐formed fibrils (PFFs) were labeled with Alexa Fluor™ 488 (Alexa Fluor™ 488 NHS Ester Succinimidyl Ester, Catalog #A20000; ThermoFisher Scientific) following the manufacturer's protocol. PFFs (2 mg·mL^−1^) were sonicated and incubated with Alexa Fluor™ 488 for 1 h at room temperature with continuous shaking at 300 rpm. Labeled fibrils were then transferred to a dialysis membrane (Slide‐A‐Lyzer™ MINI Dialysis Device, 10K MWCO, 0.1 mL, Catalog #69570; ThermoFisher Scientific) and dialyzed against PBS with magnetic stirring. The PBS buffer was replaced three times over 24 h to remove unbound dye. After dialysis, labeled PFFs were collected, aliquoted, and stored at −80 °C until use. For neuronal uptake experiments, fluorescently labeled αSyn PFFs were incubated with neurons for 6 h, and uptake was quantified using flow cytometry.

### Dextran uptake assay

HEK cells were incubated with Alexa Fluor™ 568–dextran (10 kDa, D22192; Thermo Fisher Scientific) for 1 h at 37 °C. Cells were then washed with PBS to remove extracellular dextran, detached using 0.25% trypsin‐ EDTA, and resuspended in complete medium. Cells were pelleted, washed with PBS, and resuspended in buffer (PBS with BSA and EDTA) for acquisition by flow cytometry. Dextran uptake was quantified as Alexa Fluor 568 fluorescence (median fluorescence intensity) in single cells.

### Lentiviral production and titration

HEK293T cells were plated on poly‐*d*‐lysine (PDL)‐coated dishes to enhance cell adherence and growth. Once cells reached 50–60% confluency, they were transfected using Lipofectamine 3000 (Catalog #L3000008; Thermo Fisher) following the manufacturer's protocol. The transfection cocktail included transfer plasmids carrying CRISPR guides, along with packaging plasmids: psPAX2 (Addgene #12260) and VSV‐G (Addgene #8454) to facilitate lentiviral particle production. Six hours post‐transfection, the medium was replaced with Virus Harvesting Medium, composed of DMEM supplemented with 10% fetal bovine serum (FBS) and 10 mg·mL^−1^ bovine serum albumin (BSA) to optimize viral particle collection. The viral supernatant was carefully harvested and stored appropriately. For viral titration, target cells were seeded at 2.5 × 10^5^ cells per well in PDL‐coated 24‐well plates, followed by the addition of serial dilutions of the harvested virus. Three days post‐infection, intracellular flow cytometry staining and analysis were performed to determine the transduction efficiency and quantify viral titers. Titer (transducing units per milliliter; TU·mL^−1^) was calculated based on the proportion of fluorescent‐positive cells.

### Intracellular flow cytometry staining

Cells were trypsinized, pelleted, and washed with TBS before fixation and permeabilization using Cyto‐Fast™ Fix/Perm Buffer (Catalog #426803; BioLegend) for 20 min at room temperature (RT). Following fixation, cells were washed twice with 1X Cyto‐Fast™ Perm Wash solution before incubation with Alexa Fluor® 594 81A antibody (Catalog #825708; BioLegend) for 20 min in the dark at RT. After staining, cells were washed again with 1X Cyto‐Fast™ Perm Wash solution, followed by a final wash with Cell Staining Buffer (Catalog #420201; BioLegend). The cells were then resuspended in Cell Staining Buffer and acquired on an LSR II Fortessa 4L flow cytometer for the quantification of intracellular αSyn phosphorylation (pSyn^129^).

For data analysis, flow cytometry data were exported and analyzed in flowjo v10.1. Gating was performed to exclude debris and doublets, ensuring an accurate representation of single, viable cells. CRISPR‐expressing cells were identified by BFP positivity (qgRNA expressing cells), and within this population, the percentage of Alexa Fluor® 594‐positive cells was quantified as a measure of 81A‐positive cells.

### Gene expression analysis using quantitative real‐time PCR (qRT‐PCR)

Gene expression levels were analyzed using quantitative real‐time PCR (qRT‐PCR). Total RNA was extracted using TRIzol™ Reagent (Catalog #15596018; Thermo Fisher Scientific) or the RNeasy Mini Kit (Catalog #74104; Qiagen), following manufacturer protocols. RNA purity and concentration were assessed using a NanoDrop spectrophotometer (Catalog #ND‐1000; Thermo Fisher Scientific). cDNA synthesis was performed with the QuantiTect reverse transcription kit (Catalog #205311; Qiagen, Hilden, Germany) using 1 μg of RNA per reaction. qRT‐PCR was carried out with the FastStart Universal SYBR Green Master Mix (Catalog #491385000; Sigma‐Aldrich) on a ViiA 7 Real‐Time PCR System (Catalog #4453536; Thermo Fisher Scientific) under the following thermal cycling conditions: initial denaturation at 95 °C for 10 min, followed by 40 cycles of 95 °C for 15 s and 60 °C for 1 min, with a post‐amplification melting curve analysis to verify amplicon specificity. Relative gene expression was quantified using the $2^{-\Delta \Delta C_{t}}$ method, normalized to the housekeeping gene β‐actin, and compared to control conditions. Primer sequences are listed in Table S7.

### Western blot

Cells were lysed using either lysis buffer (50 mm Tris pH 8, 150 mm NaCl, 1% Triton® X‐100) or RIPA buffer (RIPA 10X, Catalog #9806S; Cell Signaling Technology), supplemented with protease inhibitors (Complete EDTA‐free Protease Inhibitor Cocktail, Catalog #11873580001; Sigma‐Aldrich) and phosphatase inhibitors (PhosSTOP, Catalog #4906845001; Roche). Protein concentrations were determined using the Bicinchoninic Acid (BCA) Protein Assay (Pierce™ BCA Protein Assay Kit, Catalog #A55864; Thermo Scientific™) according to the manufacturer's instructions. Lysates were resolved by SDS/PAGE and transferred onto PVDF membranes (iBlot™ 2 Transfer Stacks, PVDF, Catalog #IB24002; Thermo Scientific™) using the Invitrogen™ iBlot™ 2 Gel Transfer Device. Membranes were blocked with 5% SureBlock (Catalog #SB232010‐500G; LubioScience) in PBST (0.1% Tween, Catalog #9005‐64‐5, Sigma, in 1X PBS) for 1 h at room temperature. Primary antibody incubation was performed overnight at 4 °C with shaking at optimized dilutions, followed by three 10‐min washes in PBST (PBS + 0.2% Tween). All the antibodies used for western blot with specific dilutions are listed in Table S6.

For secondary detection, membranes were incubated for 45 min at room temperature with HRP‐conjugated respective secondary antibodies at 1 : 10 000 dilution. After three 10‐min washes in PBST, signals were developed using Classico, Crescendo, or Forte ECL solutions (Immobilon Western HRP substrate, Merck Millipore, Darmstadt, Germany) depending on signal intensity. β‐actin was used as a loading control, where membranes were blocked for 1 h, followed by a 30‐min incubation with β‐actin HRP‐conjugated antibody (anti‐actin HRP, Catalog #A3854‐200UL; Sigma‐Aldrich). Signal acquisition was performed immediately after three 10‐min washes in PBST. Uncropped/unadjusted western blot images are available as Data S1.

### Triton X‐100 and SDS fractionation assay

Biochemical fractionation assay was adapted based on the protocol outlined in a previous study [48]. Initially, cells were detached using Trypsin (0.05% Trypsin‐EDTA (1×), phenol red, Catalog # 25300096; Invitrogen) and washed with ice‐cold PBS. Following this, the cells were lysed using STET buffer (comprising 150 mm NaCl, 50 mm Tris pH 7.6, 1% Triton X‐100, and 2 mm EDTA), which was supplemented with both protease (Complete EDTA‐free Protease Inhibitor Cocktail, Catalog #11873580001; Sigma Aldrich) and phosphatase inhibitors (PhosSTOP, Catalog #4906845001; Roche, Basel, Switzerland). This lysate was then incubated on ice for 30 min. The lysates were then subjected to centrifugation at 17 000 ***g*** for 1 h at 4 °C. The resulting supernatant was referred to as the Triton soluble fraction or Tx‐soluble.

The residual pellet was washed twice with ice‐cold PBS and dissolved in a freshly prepared 2% SDS buffer (containing 150 mm NaCl, 50 mm Tris pH 7.6, 2% SDS, and 2 mm EDTA, also supplemented with protease and phosphatase inhibitors) and processed at room temperature. This pellet underwent sonication for cycles of 6 min on, followed by a 2‐min off interval, then again for 2 min on, using a high‐power water bath sonicator. This resulted in what is referred to as the SDS‐soluble fraction. Before electrophoresis, the Triton‐extractable fraction was heated to 95 °C, whereas the SDS‐extractable fraction was heated to 42 °C, both for 10 min. Fractions were mixed with Laemmli buffer+ DTT prior to heating and SDS/PAGE.

### Co‐immunoprecipitation (Co‐IP)

CRISPRo HEK cells were washed with PBS and lysed in CHAPS lysis buffer (25 mm Tris, 150 mm NaCl, 1 mm EDTA, 1% CHAPS, 5% glycerol, pH 7.4) supplemented with protease and phosphatase inhibitors. Protein concentration was determined using the BCA assay, and 1 mg of total lysate was diluted in CHAPS lysis buffer to a final volume of 500 μL per tube. For immunoprecipitation, Recombinant Anti‐EMC4 antibody (Catalog #ab184544; Abcam) was conjugated to Dynabeads™ Protein G (Catalog #10003D; Thermo Fisher Scientific) for 1 h at 4 °C on a spinning wheel at 30 rpm. Normal Rabbit IgG (Catalog #12‐370; Merck) was used as an isotype control. Lysates were pre‐cleared (Input Lysate) to remove nonspecific binding, then incubated overnight at 4 °C with antibody‐conjugated beads under constant rotation (30 rpm). After incubation, tubes were placed on a magnetic rack, and the cleared supernatant (flow‐through, FT) was collected. The supernatant from the EMC4 IP was referred to as EMC4 FT, and the flow‐through from the IgG control was referred to as IgG FT. Beads were then washed four times with 200 μL of CHAPS lysis buffer per tube to remove unbound proteins. Immunoprecipitated proteins were eluted in 20 μL of 2X SDS Loading Buffer (LB) with DTT (DL‐Dithiothreitol, Catalog #10708984001) and subjected to SDS/PAGE. After electrophoresis, proteins were transferred onto PVDF membranes and immunoblotted with mouse anti‐αSyn antibody (Syn 211, Catalog #AHB0261; Thermo Fisher Scientific). Membranes were subsequently stripped and re‐probed for EMC4 to confirm successful immunoprecipitation.

### Lactate dehydrogenase (LDH) release assay

Lactate dehydrogenase (LDH) release was measured using the LDH‐Glo™ Cytotoxicity Assay (Catalog #J2380; Promega) according to the manufacturer's instructions. LDH storage buffer was freshly prepared at a final concentration of 200 mm Tris/HCl (pH 7.3), 10% Glycerol, and 1% BSA, stored at 4 °C, and used for diluting samples. Following experimental treatments, 5 μL of supernatant was carefully collected from each well to avoid disturbing adherent cells and transferred to a white‐walled opaque plate. To determine maximum LDH release, a subset of wells was treated with 0.2% Triton X‐100, serving as a positive control for complete cell lysis. Samples were diluted in LDH storage buffer. LDH detection enzyme mix was combined with the Reductase Substrate and thoroughly mixed with the diluted samples in LDH buffer (1 : 1) in an opaque plate. Plates were incubated at room temperature in the dark for 60 min, after which luminescence was measured using an EnVision plate reader (PerkinElmer). LDH release values were normalized and expressed as relative cytotoxicity.

### CellTiter‐Glo/cell viability assay

Intracellular ATP levels were measured using the CellTiter‐Glo® Luminescent Cell Viability Assay (Catalog #G7572; Promega) according to the manufacturer's instructions. Briefly, cells and cellTiter‐Glo® reagent were equilibrated to room temperature, an equal volume of CellTiter‐Glo reagent was added to each well, and plates were shaken to induce cell lysis. After incubation at room temperature to stabilize the luminescent signal, luminescence was recorded on a plate reader and reported as relative luminescence units (RLU). Values were normalized to the non‐targeting control.

### MitoSOX‐based detection of mitochondrial ROS

Mitochondrial reactive oxygen species (ROS) were measured using MitoSOX™ (Catalog #M36007; Thermo Fisher Scientific). As a positive control, cells were treated with 2 μm rotenone (Catalog #R8875‐1G; Sigma‐Aldrich) for 2 h to induce ROS. A 5 mm MitoSOX stock solution was prepared by dissolving 13 μL of anhydrous DMSO into the supplied aliquot. The working solution was prepared by diluting MitoSOX to a final concentration of 1 μm in colorless Opti‐MEM medium. Cells were incubated with MitoSOX and Hoechst 33342 (Catalog #62249; Thermo Fisher Scientific) for 30 min at room temperature (RT), protected from light. After incubation, cells were gently washed 3× with pre‐warmed medium. Live‐cell imaging was performed at 20× or 40× magnification using the GE IN Cell Analyzer.

### Mitochondrial membrane potential measurement

Mitochondrial membrane potential (ΔΨm) was assessed using Image‐iT™ TMRM Reagent (Catalog #I34361; Thermo Fisher Scientific). Nuclear labeling was performed using Hoechst 33342 (Catalog #62249; Thermo Fisher Scientific) at a 1 : 2000 dilution from a 10 mg·mL^−1^ stock solution. Cells were incubated with 50 nm TMRM for 30 min at 37 °C to establish baseline ΔΨm. Regions of interest (ROI) were selected to ensure coverage of at least 1000 cells per condition. Following baseline measurement: Oligomycin (2 μm, Catalog #ab141829; Abcam) was added to inhibit ATP synthase (Complex V), initially increasing ΔΨm due to reduced proton leakage. TMRM fluorescence was recorded every 3 min for 15 min to monitor changes in mitochondrial polarization. FCCP (2 μm, Catalog #ab120081; Abcam) was then added to collapse ΔΨm, inducing complete mitochondrial depolarization. TMRM fluorescence was measured every 3 min for an additional 15 min to assess mitochondrial uncoupling. Images were consistently captured from the same ROI throughout the experiment. ΔΨm changes were analyzed by comparing TMRM fluorescence intensity before and after treatments to quantify mitochondrial depolarization.

### Lysosomal and proteasomal inhibition

MG132 (Catalog #C2211, stock solution 5 mm; Sigma‐Aldrich) and Bafilomycin A1 (BafA1, Catalog #BML‐CM110, stock solution 1 mm; Enzo Life Sciences, Farmingdale, NY, USA) were prepared in DMSO and stored at −20°C. For proteasomal inhibition, cells were treated with MG132 (5 μm) for 48 h, with media replenished every 24 h. For lysosomal inhibition, cells were treated with BafA1 (50 nm) for 18 h following αSyn PFF addition. Equivalent volumes of DMSO were used as vehicle controls. After treatments, cells were either lysed for western blot analysis or fixed for immunostaining and imaging.

### Bulk RNA sequencing

Total RNA was extracted using RNeasy Mini Kit following the manufacturer's protocol. RNA integrity and purity were assessed using a Qubit® Fluorometer (Life Technologies, Carlsbad, CA, USA) and a Fragment Analyzer (Agilent). Samples with a 260/280 nm ratio between 1.8 and 2.1 and a 28S/18S ratio between 1.5 and 2 were considered suitable for sequencing. RNA samples (100–1000 ng) underwent poly(A) enrichment and were reverse transcribed into double‐stranded cDNA using the TruSeq Stranded mRNA Kit (Illumina, San Diego, CA, USA).

cDNA libraries were enzymatically fragmented, end‐repaired, adenylated, and ligated with TruSeq adapters containing unique dual indices (UDI). Adapter‐ligated fragments were enriched by PCR amplification, and final libraries were assessed for quality and quantity using a Qubit® Fluorometer and Fragment Analyzer. The final product had an average fragment size of ~ 260 bp and was normalized to 10 nm in Tris/Cl (10 mm, pH 8.5, 0.1% Tween‐20) before sequencing. Sequencing was performed on an Illumina NovaSeqX platform using a paired‐end 2 × 150 bp strategy. Raw reads were pre‐processed using fastp [49] with the following parameters: *‐‐trim_front1 1 ‐‐cut_tail 20 ‐‐trim_poly_x ‐‐poly_x_min_len 10 ‐‐length_required 18*.

Transcript quantification was performed with kallisto using the GRCh38.p13 reference transcriptome (Annotation Release 42, downloaded on 2023‐01‐30, doi: 10.1038/nbt.3519). Differential gene expression analysis was conducted using edger [50]. All computational analyses, including Gene Set Enrichment Analysis (GSEA) and Over‐Representation Analysis (ORA), were executed via the SUSHI platform at the Functional Genomics Center Zurich [51].

### Statistical analysis

Statistical tests were performed using rstudio (R). For the primary screening datasets, differential effects were assessed using limma moderated *t*‐tests (empirical Bayes variance estimation). As a multiple‐testing assessment, Benjamini–Hochberg FDR correction was evaluated across all genes within each primary screen; however, no genes reached FDR < 0.05. Therefore, the primary CRISPRa/CRISPRo screens were treated as hypothesis‐generating and used for candidate prioritization based on nominal (unadjusted) two‐sided *P* values from limma (reported in Tables S1, S2), pre‐specified effect‐size thresholds, and cell‐count filtering. Confirmatory statistics were applied in the secondary screen with increased technical replicates using appropriate multiple‐comparison testing. For correlation test, data were initially evaluated for normality using the Shapiro–Wilk test (*α* = 0.05). Data that passed the normality assumption were analyzed with Pearson's correlation whereas data violating normality assumptions Spearman's correlation was used. For multiple comparisons, one‐way ANOVA followed by Dunnett's *post hoc* test was applied, while two‐tailed Student's *t*‐test or Welch's *t*‐test (for unequal variance) was used for two‐group comparisons. The primary CRISPR screens were performed in duplicate wells (technical duplicates). All experiments included at least three biological replicates, with 3–4 technical replicates per condition. iPSC‐derived neuronal experiments were based on three independent differentiations. Detailed statistical tests and significance values are reported in the figure legends.

## Results

### A high‐content CRISPR screening platform to identify regulators of αSyn aggregation

To establish a microscopy‐based CRISPR screen for genetic regulators of αSyn aggregation, we used HEK293 cells stably overexpressing human wild‐type (wt) αSyn (henceforth named HEK^Syn^). Despite being immortalized, HEK293 cells share some biochemical features with neurons [52] and provide the scalability and susceptibility to genetic manipulation necessary for arrayed high‐throughput screens. Upon addition of exogenous αSyn pre‐formed fibrils (PFFs), HEK^Syn^ cells form intracellular pSyn^129^ aggregates that resemble Lewy body‐like inclusions [42].

**Fig. 1:**
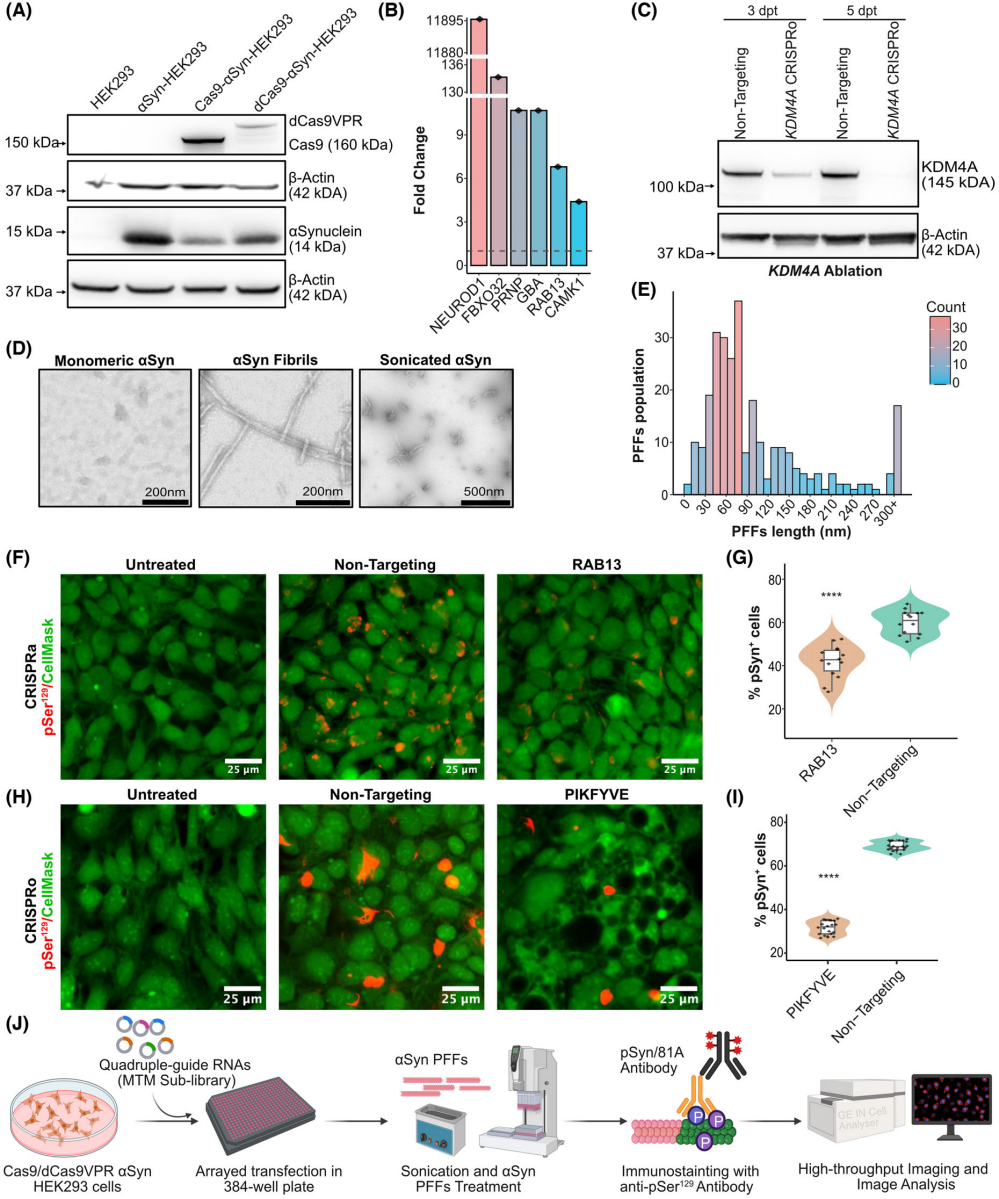
CRISPR screening workflow and αSynuclein (αSyn) phosphorylation assay in a cellular model. (A) Western blot analysis of HEK293 clones overexpressing αSyn (αSyn‐HEK293/HEK^Syn^) with either dCas9‐VPR (CRISPRa) or Cas9 (CRISPRo). (B) RT‐qPCR of arbitrarily selected genes for the assessment of dCas9‐VPR activity normalized to non‐targeting controls. (C) Gene ablation efficiency/Cas9 activity on the cells measured at 3‐ and 5‐days post‐transfection (dpt). (D) Representative negative‐stain TEM micrographs of *in vitro* produced αSyn PFFs. (E) Distribution of quantified sonicated PFFs lengths. (F, H) Representative immunofluorescence of HEK293 cells showing pSyn aggregates (red) and cytosol (green). Scale bar, 25 μm. (G, I) Quantification of p‐Ser129^+^ cells normalized to total DAPI count. Inner box plots: median (center line), 75th and 25th percentile (top and bottom edge). (J) Schematic representation of the high‐content arrayed CRISPR screening assay for phosphorylated αSynuclein at Ser129 (pSyn) detected using antibody 81A. Welch's *t*‐test (unequal variance *t*‐test, ****P < 0.0001).

We generated HEK^Syn^ clones expressing Cas9 or the tripartite programmable transactivator dCas9‐VPR [53] for CRISPR ablation (CRISPRo) and CRISPR activation (CRISPRa), respectively (Fig. 1A). We isolated and expanded five single‐cell clones for each of the CRISPRo and CRISPRa lines. One clone per condition was then selected after evaluating (i) stable expression of Cas9 or dCas9‐VPR, (ii) stable overexpression of αSyn, and (iii) the ability to form intracellular αSyn aggregates upon exposure to αSyn PFFs. These clones retained the CRISPR machinery, αSyn expression, and generated pSyn^129^ aggregates after exposure to PFF, confirming their suitability for all subsequent experiments. To modulate gene expression, we used the T.gonfio and T.spiezzo arrayed libraries [41] which target each gene with four non‐overlapping single guide RNAs (qgRNAs). We then optimized transfection conditions for high qgRNA delivery efficiency in 384‐well format (Fig. S1). CRISPRa lines showed robust transcriptional upregulation, with mRNA expression levels increasing up to ~ 10 000‐fold (Fig. 1B), and western blotting confirmed elevated expression of a representative target protein (Fig. S1). Similarly, CRISPR‐based gene ablation in CRISPRo lines with randomly selected qgRNAs resulted in a ~ 95% reduction in target gene expression within 5 days after transfection (Figs 1C and S1). Next, we generated αSyn PFFs from purified monomeric human wt αSyn and confirmed their amyloid fibrillar structure by negative‐stain transmission electron microscopy (TEM), SDS/PAGE, and Thioflavin T (ThT) fluorescence assays (Figs 1D and S1). Since smaller fibrils exhibit potent seeding capability [54], we sonicated the PFFs preparation to obtain a heterogeneous population of fibrillar fragments (average length: 30–100 nm; Fig. 1E). We then treated HEK^Syn^ cells with sonicated PFFs mixed with the transfection reagent LT1 to facilitate efficient intracellular delivery through lipid‐based complex formation. As a proxy for αSyn aggregation, we quantified pSyn^129+^ inclusions [55, 56]. Confocal imaging confirmed the presence of pSyn^129^ within the cell body exclusively in PFFs‐treated αSyn overexpressing cells (Fig. S1).

PFF treatment induced a robust cytoplasmic pSyn^129^ signal in α‐Syn–overexpressing HEK^Syn^ cells, whereas no cytoplasmic pSyn^129^ signal was detected in parental HEK293 cells that do not overexpress αSyn (Fig. S1). This indicates that the pSyn^129^ signal is specific for endogenous, newly aggregated αSyn and is not attributable to the exogenous PFF seed. Immunoblotting with pSyn^129^ antibody D1R1 confirmed that PFF exposure to HEK^Syn^ robustly induces different molecular weight pSyn^129^ species (Fig. S1). Consistent with seeded aggregation, biochemical fractionation revealed increased accumulation of total αSyn (including high–molecular weight species) in the SDS soluble/Triton‐X100 (Tx) insoluble fraction in PFF‐treated cells (Fig. S1). Monomeric αSyn levels in the Tx‐soluble fraction were lower in the PFF‐treated sample than in the control, suggesting the formation/accumulation of aggregated species in the Tx‐soluble fraction. Collectively, these findings lay the foundation for a platform enabling the systematic interrogation of genetic regulators of αSyn aggregation.

To monitor the quality and reliability of the screen, we incorporated *RAB13* and *PIKFYVE* as positive control genes, which have been previously reported to modulate pSyn^129^ levels. For the CRISPRa screen, we used *RAB13* which has been shown to reduce αSyn aggregates upon overexpression [33]. For the CRISPRo screen, we used *PIKFYVE*, whose inhibition [57] decreases αSyn aggregation. High‐throughput fluorescence imaging revealed a significant reduction in percentage of pSyn^129+^ cells with activated *RAB13* (Fig. 1F,G). Likewise, ablation of *PIKFYVE* resulted in cytoplasmic vacuolation and a decrease in the percentage of pSyn^129+^ cells (Fig. 1H,I). These results validate the robustness of our screening platform and establish stringent quality‐control metrics for identifying genetic modulators of pSyn^129^.

### Arrayed CRISPRa and CRISPRo screens identify modulators of pSyn^129^

Mitochondrial dysfunction is a key contributor to PD pathology [58]. Recent reports suggest that the inner and outer mitochondrial membranes act as primary sites for early aggregation events [59]. Hence, we conducted CRISPRa and CRISPRo screens targeting genes involved in mitochondrial homeostasis and function, intracellular trafficking and cytoskeletal reorganization. HEK^Syn^ cells expressing Cas9 or dCas9‐VPR were transfected with 2304 or 2428 individual qgRNA plasmids, respectively, as duplicates in separate 384‐well plates, selected with puromycin for 48 h (CRISPRa) or 96 h (CRISPRo). Cells were then treated for 72 h with sonicated PFFs delivered via lipofection using the LT1 reagent (Fig. 1J). Cell nuclei were stained with DAPI, pSyn^129^ aggregates were labeled using the 81A antibody, and the entire cell was uniformly labeled with HCS CellMask™ Deep Red. Images were acquired using automated widefield imaging systems and analyzed by semi‐automated machine learning‐based pixel classification (ilastik) in combination with object segmentation (CellProfiler) (Fig. S2A). We created two ilastik probability maps: one for the DAPI‐stained nuclei and one for the pSyn^129^ aggregates. These maps were then imported into CellProfiler, where the nuclear map served as a reference to segment cells based on the CellMask channel. The pSyn^129^‐aggregate map was overlaid with the segmented cells to determine the number of cells positive for at least one aggregate. We defined the fraction of pSyn^129+^ cells as the number of cells containing at least one aggregate divided by the total number of segmented cells. The robustness of the primary screens was quantitated by calculating strictly standardized mean difference (SSMD) scores based on non‐targeting qgRNAs (NTG) and moderate‐strength positive controls. CRISPRa and CRISPRo screens achieved SSMD values above 1 and 2, respectively (Fig. S2B–E). These metrics indicate a good quality of the screen [60]. Next, we evaluated reproducibility between replicates using correlation analyses suited to each dataset's distribution. Since the CRISPRa data followed a normal distribution, we applied Pearson's correlation coefficient (*r* = 0.75, adjusted *R* ^2^ = 0.59; Fig. S2C). In contrast, the CRISPRo data deviated from normality, so we used Spearman's rank correlation coefficient (*ρ* = 0.856; Fig. S2F). Replicates from both screens correlated strongly, demonstrating technical reproducibility. To visualize the frequency distribution of individual samples for all targeted genes, we plotted histograms of the median values of pSyn^129+^ cell fraction. Most genes showed no significant impact on pSyn^129+^ levels (Fig. S2D,G).

**Fig. 2:**
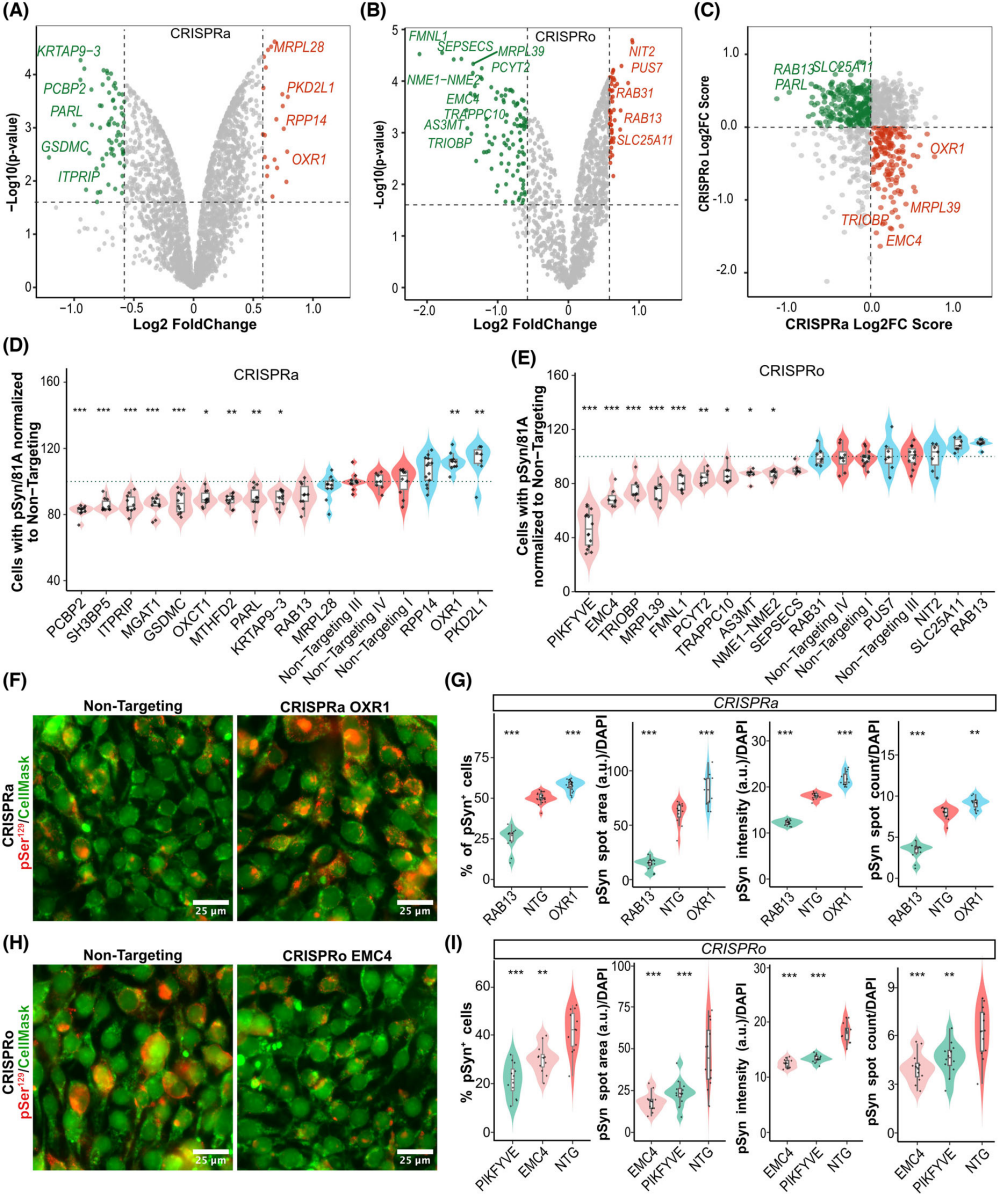
CRISPRa/CRISPRo screens for genetic modulators of αSynuclein aggregation. (A, B) Volcano plots showing log_2_ fold change versus −log_10_ (*P*‐value) from limma moderated *t*‐tests (nominal, unadjusted), highlighting genes increasing (red) or decreasing (green) pSyn^129^ levels. (C) Cross‐tabulation of effect sizes in CRISPRa vs CRISPRo screens. Green and red: bidirectional modifiers. (D, E) Validation of hits with increased replicates. (F, H) pSyn^129+^ aggregates in HEK^Syn^ cells following OXR1 activation (F) or EMC4 ablation (H). CellMask: red; pSyn^129+^ aggregates: yellow; DAPI: blue. Scale bar, 25 μm. (G, I) Quantification of pSyn^129+^ cells, aggregate area arbitrary units (a.u.) per DAPI, aggregate intensity (a.u.) per DAPI, and aggregate count (per DAPI) following OXR1 activation (G) or EMC4 (I) ablation. NTG: Non‐Targeting. Inner box plots display the median (center line), the 75th and 25th percentile (top and bottom edge). One‐way ANOVA followed by Dunnett's *post hoc* test; *P < 0.05, **P < 0.01, ***P < 0.001.

Primary hit lists were nominated using mean log_2_‐transformed fold change (log_2_FC > 0.58 and < −0.58 for up‐ and downregulators, respectively), nominal moderated *t*‐test *P*‐value using limma in R (moderated *t*‐test *P* < 0.01; Table S1: CRISPRa, and Table S2: CRISPRo). The cutoff values for the log_2_FC account for the overall baseline variability among the NTG controls. To correct for multiple testing, we calculated Benjamini–Hochberg FDR‐adjusted *P* values across all genes within each primary screen; no genes reached FDR < 0.05 in the primary dataset, which may be due to the limited replication of the primary screen (duplicate wells). We therefore treated the primary screens as hypothesis‐generating and used them to prioritize candidates for confirmatory secondary screening with increased replication and orthogonal validation. To minimize artifacts driven by cytotoxicity or poor segmentation, wells with total cell counts < 50% of the non‐targeting median were discarded. Both CRISPR screens identified multiple genetic modifiers of pathological pSyn^129^, using the percentage of pSyn^129+^ cells as a readout of aggregated pSyn^129^ levels (Fig. 2A,B). As a benchmark control, RAB13 perturbation showed the expected directional effects on pSyn129^+^ prevalence. *RAB13* overexpression (CRISPRa screen) reduced pSyn129^+^ prevalence, whereas *RAB13* ablation (CRISPRo screen) enhanced pSyn129^+^ levels (Fig. 2D,E), consistent with previous findings [33], further validating the robustness of our assay. To assess the bidirectional regulatory effects of the identified hit genes on pSyn^129+^ prevalence, we compared log_2_FC scores between CRISPRa and CRISPRo screens (Fig. 2C). Genes with opposing effects clustered in distinct quadrants, with CRISPRa upregulators acting as downregulators in CRISPRo, and vice versa. However, bidirectional effect sizes were modest, suggesting that while such regulation exists, its impact on pSyn^129+^ prevalence may be limited.

### Secondary screen identified EMC4 and OXR1 as strong modulators of pSyn^129^

To refine our candidate selection and eliminate false positives, we retested all selected hits using additional technical replicates. Each candidate gene was statistically compared to the non‐targeting control group using a one‐way ANOVA followed by Dunnett's *post hoc* test. Most hits maintained the same directional trend observed in the primary screen, with 11 of 13 CRISPRa hits and 9 of 14 CRISPRo hits reaching statistical significance (*P* < 0.05; Fig. 2D,E). In the CRISPRa screen, *PCBP2, SH3BP5, ITPRIP, MGAT1, GSDMC, OXCT1, MTHFD2, PARL*, and *KRTAP9‐3* were confirmed as pSyn^129^ downregulators whereas *OXR1* and *PKD2L1* emerged as upregulators (Fig. 2D). For the CRISPRo screen, *EMC4, TRIOBP, MRPL39, FMNL1, PCYT2, TRAPPC10, AS3MT*, and *NME1‐NME2* were confirmed as pSyn^129^ downregulators (Fig. 2E). *RAB13* was identified as an upregulator alongside *SLC25A11*, although the latter did not reach statistical significance.

**Fig. 3:**
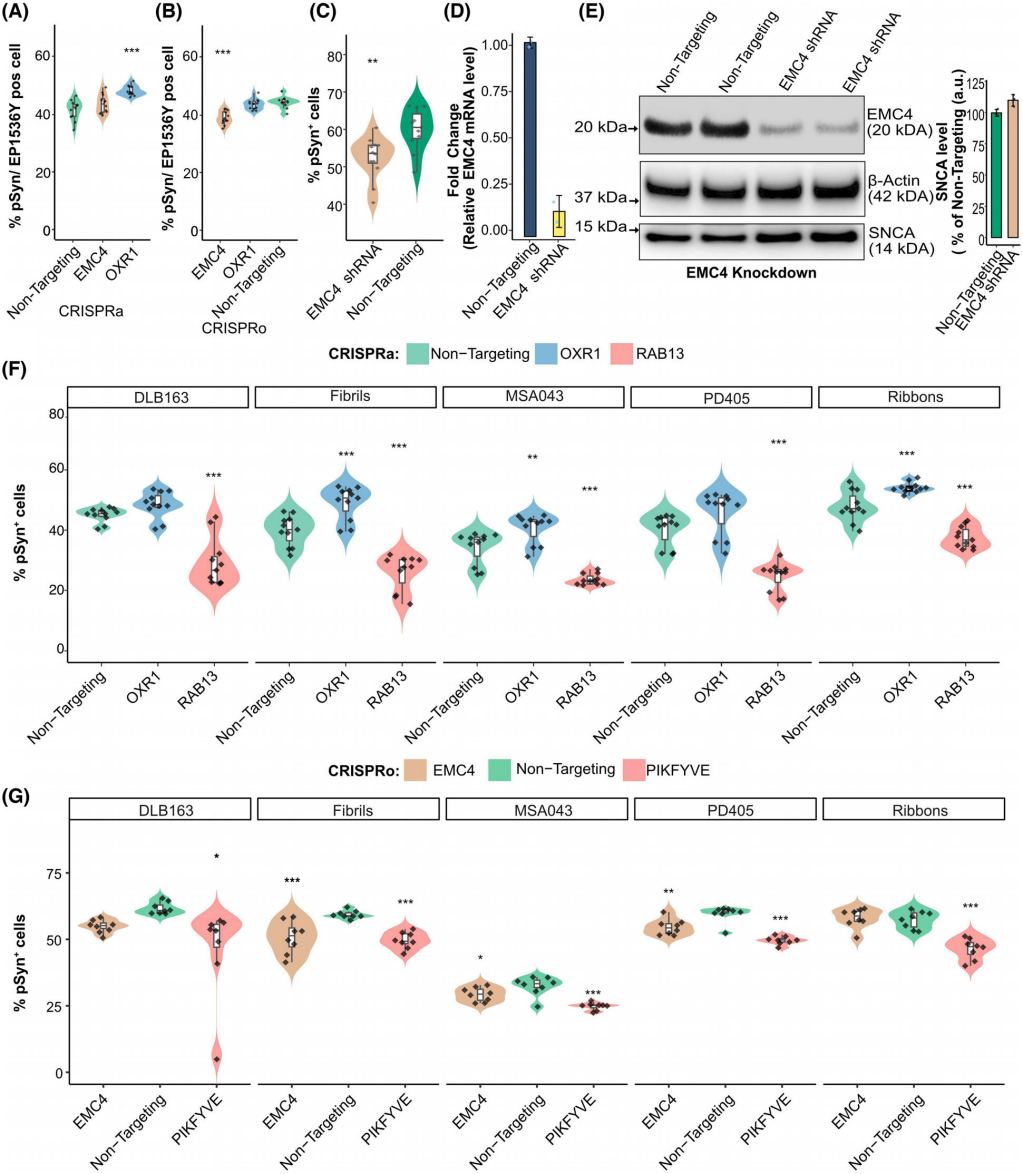
Differential effects of modifiers across different patient‐derived and recombinant fibrillar strains and orthogonal hit validation in HEK^Syn^ cell lines. (A, B) pSyn^129^ levels following EMC4 and OXR1 perturbations (EP1536Y antibody). (C) shRNA‐mediated inhibition of EMC4 and its impact on pSyn^129^ (81A antibody). Welch's *t*‐test (unequal variance *t*‐test, *P < 0.05, **P < 0.01). (D) EMC4 mRNA levels after shRNA‐mediated knockdown (RT‐qPCR, Mean ± SEM). (E) Representative western blot showing EMC4 protein and SNCA protein after shRNA‐mediated inhibition (left) and quantification of SNCA levels normalized to β‐actin, shown as percentage of the non‐targeting control. Bar plots: mean ± SEM. (F, G) Effect of OXR1 activation (f) or EMC4 ablation (G) on pSyn^129^ for human PD, DLB, and MSA patient‐derived fibrils as well as recombinant fibrils and ribbons (81A antibody). Inner box plots display the median (center line), the 75th percentile (top edge), and the 25th percentile (bottom edge). One‐way ANOVA followed by Dunnett's *post hoc* test. *P < 0.05, **P < 0.01, ***P < 0.001.

To determine whether any of our hits have previously been implicated in PD, we obtained a PD‐associated gene set from the Open Targets Platform (https://platform.opentargets.org/). Open Targets dataset compiles genes from GWAS and expression studies, as well as literature‐curated and other disease‐linked genes. Among our validated hits, only GSDMC (Gasdermin C) overlapped with the genes from the Open Targets dataset (Fig. S2H) [61, 62]; however, its modulation of pSyn^129^ was minimal and was not further investigated. Instead, we focused on the two strongest and most reproducible modifiers: the mitochondrial Oxidation Resistance Gene 1 (*OXR1*) and the ER Membrane Protein Complex Subunit 4 (*EMC4/TMEM85*). Both genes are highly expressed in the human brain, including neurons (https://www.proteinatlas.org/) [63]. CRISPR‐based manipulation of *OXR1* and *EMC4* did not affect cellular viability as indicated by lactate dehydrogenase (LDH) release assay (Fig. S3A,B), nor cell density as shown by quantification of DAPI‐positive nuclei (Fig. S3F,G). pSyn^129^ was assessed with the 81A and EP1536Y antibodies. *OXR1* upregulation caused a significant increase in the fraction of pSyn^129+^ cells, total pSyn^129^ spot area, total pSyn^129^ spot intensity, and the number of pSyn^129^ puncta divided by the number of DAPI+ nuclei (Figs 2F,G, 3A, and S3D). This effect was further supported by flow cytometry, which showed a consistent trend, albeit with lower effect size (Fig. S4A,B). Western blotting confirmed increased OXR1 protein levels in CRISPRa‐activated cells (Fig. S2K). RT‐qPCR confirmed that CRISPRa activation of OXR1 led to a substantial increase in OXR1 mRNA levels (Fig. S2L).

Similarly, ablation of *EMC4* significantly reduced the fraction of pSyn^129+^ cells, total pSyn^129^ spot area, total pSyn^129^ spot intensity, and the number of pSyn^129^ puncta (Figs 2H,I, 3B, and S3E). Similar results were obtained by flow cytometry (Fig. S4A,C). Although CRISPR‐based ablation of *EMC4* led to a substantial reduction in its mRNA levels, we were unable to achieve complete depletion of the EMC4 protein (Fig. S2I,J). Attempts to isolate stable clonal lines lacking EMC4 entirely were unsuccessful: the few clones that initially survived showed poor viability and failed to expand beyond one or two passages, suggesting that complete *EMC4* ablation may compromise cell fitness. This is consistent with data from the International Mouse Phenotyping Consortium (MGI : 1915282), which indicates that homozygous *EMC4* knockout severely compromises viability in mice. Nonetheless, the partial EMC4 depletion was sufficient to significantly reduce pSyn^129+^ prevalence.

We then tested the effect of partially reducing *EMC4* expression through a shRNA‐based approach, rather than CRISPR. Knockdown of *EMC4* through shRNA recapitulated the phenotypic effects observed in the CRISPRo experiments, including significant reduction in pSyn^129^‐positive cells (Figs 3C and S3C). This effect was accompanied by a marked reduction in *EMC4* mRNA and protein levels (Fig. 3D,E). We next examined potential bidirectional effects but found that OXR1 ablation and EMC4 activation did not alter pSyn^129^ levels in HEK^Syn^ cells (Fig. S5C,D). Since altered pSyn^129^ accumulation might be a result of altered endocytosis of the exogenous PFF acting as seeds, we measured internalization of 10 kDa Alexa Fluor 568–dextran by flow cytometry upon CRISPR‐based perturbation of candidate genes. Neither OXR1 activation nor EMC4 ablation significantly changed dextran uptake compared with non‐ targeting controls, indicating that the pSyn^129^ phenotypes are unlikely to be explained by altered bulk endocytosis/uptake in HEK cells (Fig. S4D,E).

### Strain‐specific effects of OXR1 activation and EMC4 depletion on pSyn^129^ levels

Synucleinopathies encompass diverse disorders, each defined by structurally distinct αSyn assemblies. These strains exhibit unique pathogenic properties, reflecting their clinical origins [43, 44, 64]. We tested the effect of *OXR1* and *EMC4* perturbation across diverse αSyn assemblies: patient‐derived strains (PD, MSA, DLB) and recombinant polymorphs (fibrils, ribbons). Activation of OXR1 led to a moderate increase in pSyn^129+^ prevalence across all strains, with statistically significant effects observed for recombinant polymorphs and MSA‐derived fibrils (Figs 3F and S5A), suggesting that strain conformation influences OXR1‐mediated aggregate accumulation. In contrast, EMC4 depletion modestly but significantly reduced pSyn^129+^ prevalence across PD, MSA, and fibrillar strains (Figs 3G and S5A), supporting its function as a broad‐spectrum modulator of αSyn aggregation.

### OXR1 overexpression impairs ATP synthesis and elevates pSyn^129^ accumulation

**Fig. 4:**
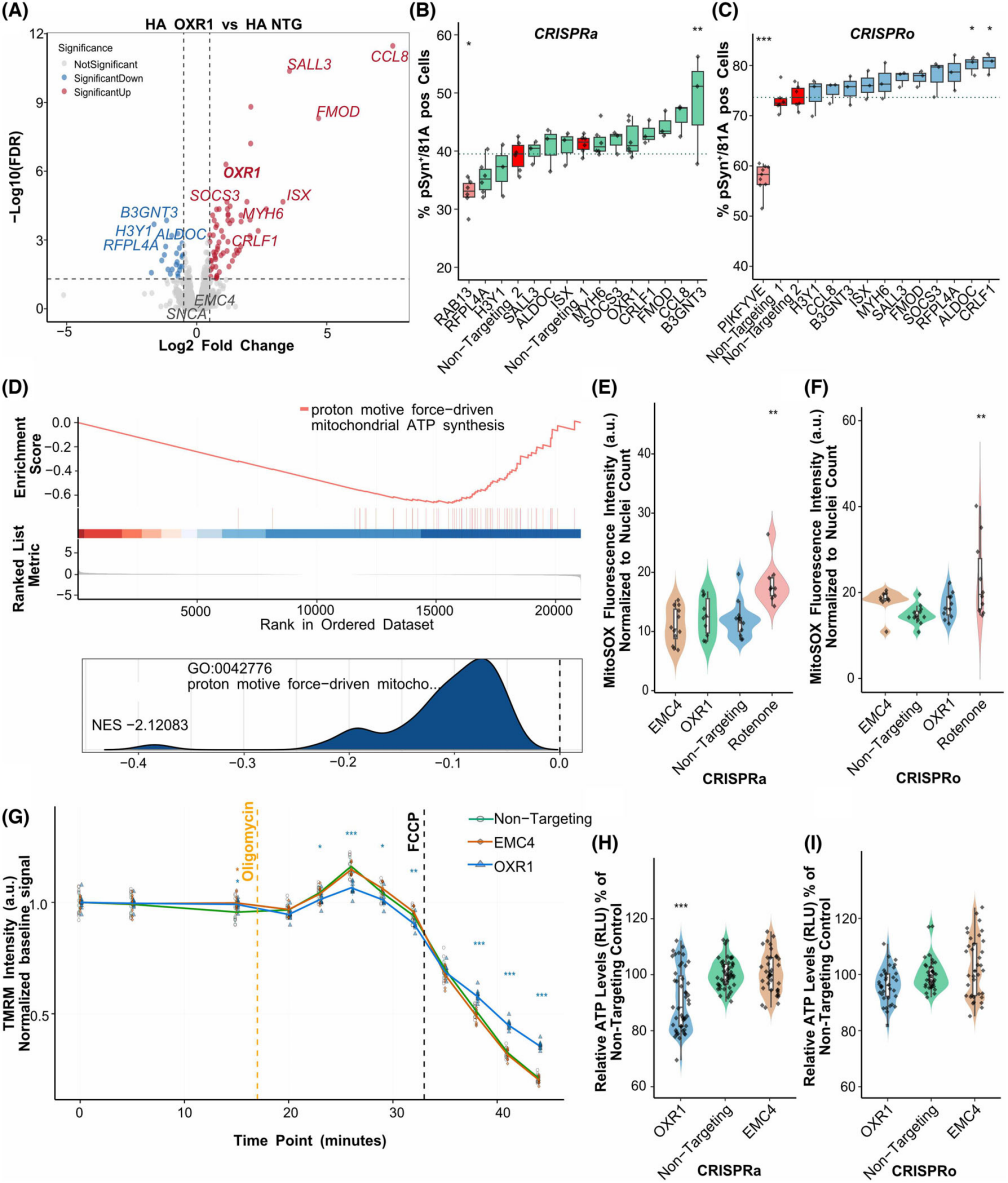
Transcriptional and functional consequences of OXR1 activation. (A) RNA sequencing showing transcripts up (red) and downregulated (blue) by OXR1 activation in HEK^Syn^ cells. NTG: non‐targeting control. (B, C) Individual perturbation of DEGs by OXR1 activation. Box plots display the median (center line), the 75th percentile (top edge), and the 25th percentile (bottom edge). (D) Gene set enrichment analysis (GSEA) highlighting the mitochondria‐related pathway with the normalized enrichment score (NES). Candidate terms are based on a false‐discovery rate ≤0.05; genes are ranked by effect size. Upper panel: running enrichment score plot; lower panel: ridge plot. (E, F) Mitochondrial superoxide levels measured by MitoSOX™ Red dye in live cells. Fluorescence intensity was normalized to the total number of cells. (G) Time‐resolved measurement of mitochondrial membrane potential (ΔΨm) using TMRM fluorescence under basal conditions and after sequential exposure to oligomycin and FCCP. Data are shown as mean ± S.D. (H, I) Intracellular ATP levels measured using CellTiter‐Glo (RLU: Relative Luminescence Units) following CRISPR activation (H) and CRISPR ablation (I). Violin plots represent data distribution. Inner box plots display the median (center line), the 75th percentile (top edge), and the 25th percentile (bottom edge). One‐way ANOVA followed by Dunnett's *post hoc* test; *P < 0.05, **P < 0.01, ***P < 0.001.

OXR1 is involved in several key processes that maintain neuronal homeostasis, including protection from oxidative stress and DNA repair [65]. To identify the downstream effectors responsible for its modulation of pSyn^129^, we compared the transcriptome of OXR1‐overexpressing HEK^Syn^ cells against HEK^Syn^ cells transduced with NTG CRISPR guides. Differentially expressed genes (DEGs) were defined by stringent filtering criteria (FDR ≤ 0.05, log_2_FC ≥ 0.5 or ≤ −0.5, *P* ≤ 0.01; Fig. 4A and Table S3). *SNCA* mRNA levels were not changed by *OXR1* activation, suggesting that OXR1 modulates pSyn^129^ levels posttranscriptionally or through indirect mechanisms (Fig. 4A). Next, we individually activated or ablated the top upregulated (*FMOD, CCL8, SALL3, SOCS3, ISX, CRLF1, MYH6*) and downregulated (*B3GNT3, H3Y1, ALDOC, RFPL4A*) genes to determine how each hit influences pSyn^129^ levels in CRISPRa HEK^Syn^ cells exposed to αSyn PFFs. pSyn^129^ was quantified by flow cytometry following 81A antibody staining (Fig. S6A). Activation of OXR1‐induced genes, *FMOD* (fibromodulin, an extracellular matrix remodeling protein) and *CCL8* (C‐C Motif Chemokine Ligand 8, an immune signaling molecule), resulted in an increased pSyn^129+^ prevalence (Fig. 4B). Similarly, ablation of the *OXR1‐*repressed genes *ALDOC* (aldolase C, a key glycolytic enzyme cleaving fructose‐1,6‐bisphosphate) and *RFPL4A* (ubiquitin protein ligase activity) also increased pSyn^129+^ prevalence (Figs 4C and S6B). Additionally, flow cytometry results were further confirmed by immunofluorescence imaging, which showed concordant changes in pSyn^129^ levels for all four genes (Figs S6C,D). These genes share OXR1 as a regulator and, when perturbed individually, promote pSyn^129^ accumulation. We next investigated the relationship between OXR1 activation and the upregulation of FMOD and CCL8, each of which individually increase pSyn^129^ prevalence. If their mechanisms of action were independent of each other, co‐activation might have synergistic effects on pSyn^129^. However, dual activation of OXR1 and either FMOD or CCL8 did not produce synergistic increases in the percentage of pSyn^129^/81A‐positive cells compared with single‐gene activation. This outcome is compatible with the notion that OXR1 and its targets share the same pathway of pSyn129 modulation (Fig. S6E).

Gene Set Enrichment Analysis (GSEA) of RNA‐seq data from HEK^Syn^ cells overexpressing OXR1 showed a significant enrichment of downregulated genes associated with the proton motive force‐driven mitochondrial ATP synthesis pathway (NES = −2.12083) (Figs 4D and S7A). The running enrichment curve indicates that genes in this pathway are significantly enriched among the most downregulated transcripts (Fig. 4D, upper panel). Key components of this pathway include *ATP5F1A, ATP5F1B* (ATP synthase subunits), *NDUFS2, NDUFS4* (Complex I), and *MT‐ATP6* (mitochondrial ATP synthase). The accompanying ridge‐density plot (Fig. 4D, lower panel) shows the distribution of pathway genes shifted toward negative log_2_FC values, confirming collective repression in OXR1‐overexpressing cells. Since these genes are linked to mitochondrial function, we examined whether OXR1 overexpression alters two key indicators of mitochondrial health: reactive oxygen species (ROS) production and membrane potential. We used MitoSOX™ Red, a fluorescent probe that selectively detects superoxide in mitochondria, to determine if OXR1‐induced changes might elevate oxidative stress. We found no significant difference in MitoSOX fluorescence between OXR1‐overexpressing HEK^Syn^ and HEK^Syn^ cells transduced with NTG guides (Figs 4E,F and S7B,C). Next, we evaluated mitochondrial membrane potential (ΔΨm) using TMRM, a dye accumulating in polarized mitochondria whose fluorescence intensity reflects membrane potential. To disrupt mitochondrial polarization, we treated cells with oligomycin to inhibit ATP synthase, followed by FCCP to induce complete depolarization [66]. Live‐cell imaging with TMRM showed minimal impact of OXR1 activation on mitochondrial membrane potential (ΔΨm) under basal conditions (Figs 4G and S7D). A transient increase in ΔΨm at 26 min (***P < 0.001) did not persist after FCCP‐induced depolarization, suggesting no sustained effect on mitochondrial polarization. These findings suggest that although OXR1 overactivation downregulates components of the ATP synthase machinery, it does not markedly alter mitochondrial ROS production and exerts only a modest, transient effect on basal membrane potential. We then measured total ATP levels using CellTiter‐Glo to test whether overall cellular ATP production was affected (Fig. 4H,I). *OXR1* activation reduced total ATP levels by ~ 15% (Dunnett's post hoc test, *P* = 2.9 × 10^− 7^) compared to non‐targeting controls (Fig. 4H), suggesting a functional role in mitochondrial ATP synthesis. However, *OXR1* ablation did not impact mitochondrial ATP production (Fig. 4I). Together, these findings show that OXR1 overexpression modestly reduces ATP levels, and mitochondrial membrane potential, while additional regulation by *FMOD*, *CCL8*, *ALDOC*, and *RFPL4A* underscores the interplay between mitochondrial function and gene‐specific modulators in driving pSyn^129^ accumulation.

### OXR1 activation exacerbates pSyn^129^ pathology in human iPSC‐derived cortical neurons

**Fig. 5:**
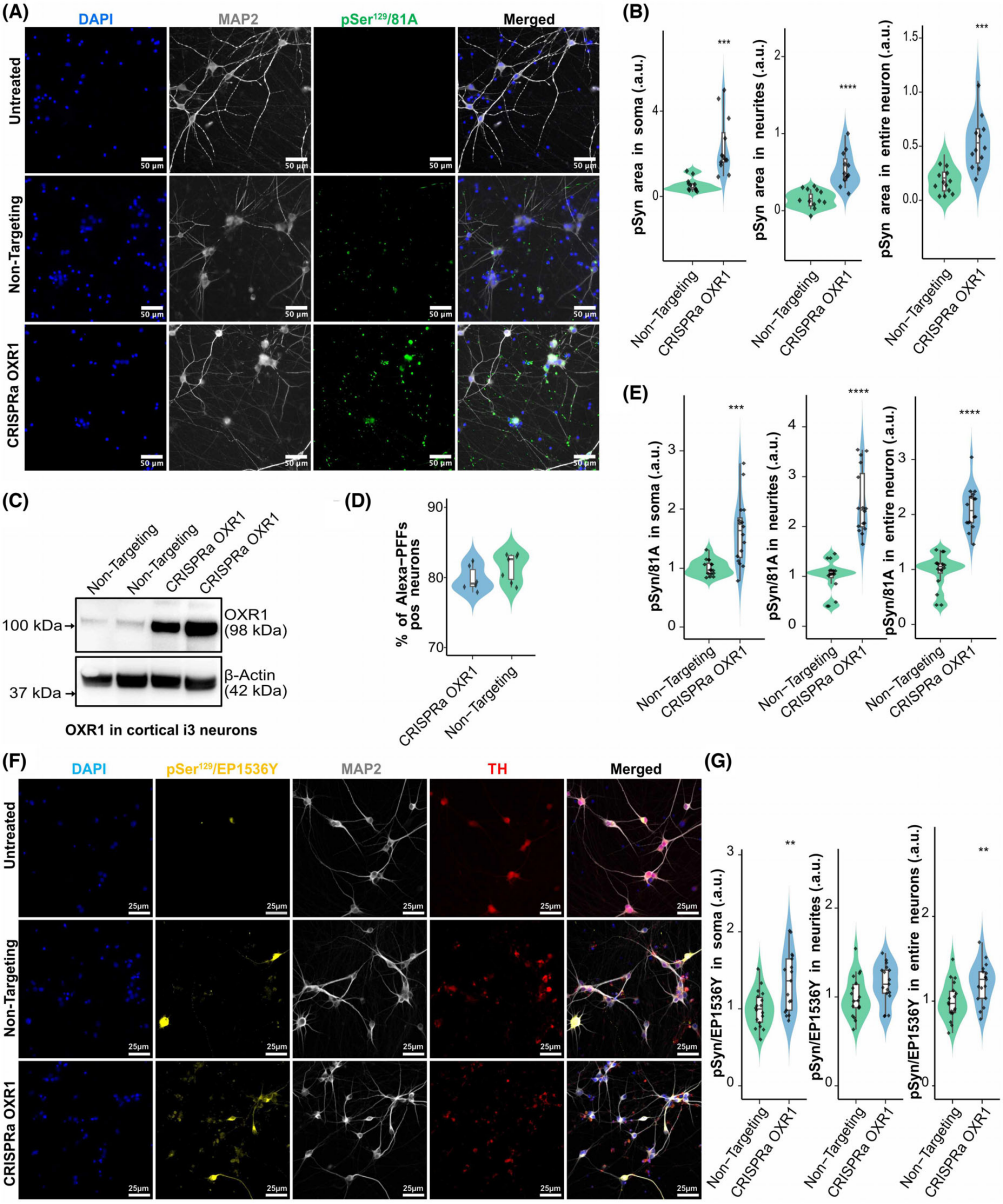
OXR1 activation modulates phosphorylated αSynuclein at Ser129 (pSyn^129^) in human iPSC‐derived cortical and dopaminergic (iDA) neurons. (A) iPSC‐derived cortical neurons stained with DAPI (blue), MAP2 (gray), and pSyn^129^ (81A, green). Scale bar, 25 μm. (B) Quantification of pSyn^129^ spot area in soma (left) neurites (middle) and whole neurons (right) per total MAP2 area. (C) Immunoblot confirming OXR1 expression in iPSC‐derived cortical neurons. (D) Flow cytometry of Alexa Fluor 488‐labeled PFFs uptake in iPSC‐derived cortical neurons. (E) pSyn^129^ levels in iDA neurons in soma, neurites, and whole neurons. (F) iDA neurons stained with DAPI (blue), MAP2 (gray), EP1536Y (yellow, pSyn^129^ aggregates), and tyrosine hydroxylase (TH; red, dopaminergic marker). Scale bar, 25 μm. (G) Quantification of pSyn^129^ spot area in soma, neurites and in entire iDA neurons normalized to MAP2 area. Box plots: median (center line), 75th), and 25th percentile (top and bottom edges). Welch's *t*‐test (unequal variance *t*‐test); **P < 0.01, ***P < 0.001, ****P < 0.001.

To test our hits in a disease‐relevant neuronal model, we assessed the effect of OXR1 activation in neurons derived from human iPSC line with an inducible NGN2 (neurogenin 2) cassette, and CRISPRa machinery dihydrofolate‐reductase destabilizing domain (DHFR) fused with transactivator dCas9‐VPH [46]. Following the lentiviral delivery of CRISPRa qgRNAs, the iPSCs were differentiated into integrated, isogenic, inducible (i3) glutamatergic cortical neurons [45, 67]. CRISPRa activity was then triggered by adding trimethoprim (TMP), which stabilizes the DHFR‐dCas9‐VPH fusion. The resulting iPSC‐derived cortical neurons were treated with αSyn PFFs for 4 weeks (Fig. S8A). Immunofluorescence staining revealed pSyn^129^ accumulation in both soma and neurites, exclusively within MAP2‐positive regions (Fig. 5A). OXR1 activation significantly increased the pSyn^129+^ puncta area in both compartments (Fig. 5B). Western blot analysis confirmed OXR1 upregulation in CRISPRa iPSC‐derived cortical neurons (Fig. 5C).

Next, we investigated whether *OXR1* activation enhances PFF internalization. We quantified Alexa Fluor 488‐labeled PFFs in iPSC‐derived cortical neurons treated for 6 h via flow cytometry. No differences were observed between *OXR1*‐activated iPSC‐derived cortical neurons and control (NTG) iPSC‐derived cortical neurons (Figs 5D and S8D), ruling out PFF uptake modulation as the cause of enhanced pSyn^129^ accumulation. Finally, western blot analyses confirmed that OXR1 activation does not alter endogenous SNCA expression (Fig. S8B,C), reinforcing that increased pSyn^129^ accumulation is not due to elevated αSyn levels. In iPSC‐derived cortical neurons, OXR1 activation was associated with a modest reduction in ATP levels (Fig. S8E), consistent with the mitochondrial phenotype observed in HEK^Syn^ cells.

### OXR1 activation promotes pSyn^129^ accumulation in human iPSC‐derived dopaminergic neurons

As pSyn^129^ accumulation mostly affects the dopaminergic neurons of the substantia nigra, we differentiated NGN2 dCas9VPH‐expressing iPSCs into dopaminergic neurons (iDA) using a rapid NGN2‐based differentiation protocol (Fig. 5F), which yields a homogeneous population of tyrosine hydroxylase (TH)‐positive neurons with mature dopaminergic features [47]. To quantify pSyn^129^ accumulation in OXR1‐overexpressing iDA neurons, we used the antibodies 81A and EP1536Y. Notably, 81A staining showed increased pSyn^129^ accumulation in both the soma and neurites, leading to an overall increase in total neuronal pSyn^129^ burden (Figs 5E and S9A). In contrast, EP1536Y staining revealed a significant increase in pSyn^129^ signal in neuronal somata (Fig. 5F,G), while neuritic levels exhibited a similar trend that did not reach statistical significance (Fig. 5G). In parallel, we quantified the pSyn^129^ phenotype specifically in tyrosine hydroxylase expressing (TH^+^) dopaminergic neurons. We generated a TH^+^ mask and quantified EP1536Y‐stained pSyn^129^ signal within TH^+^ regions, normalized to TH^+^ area. Using this TH‐restricted analysis, OXR1 activation increased pSyn^129^ burden in TH^+^ regions (Fig. S9B).

### EMC4 ablation enhances lysosomal but not proteasomal degradation

**Fig. 6:**
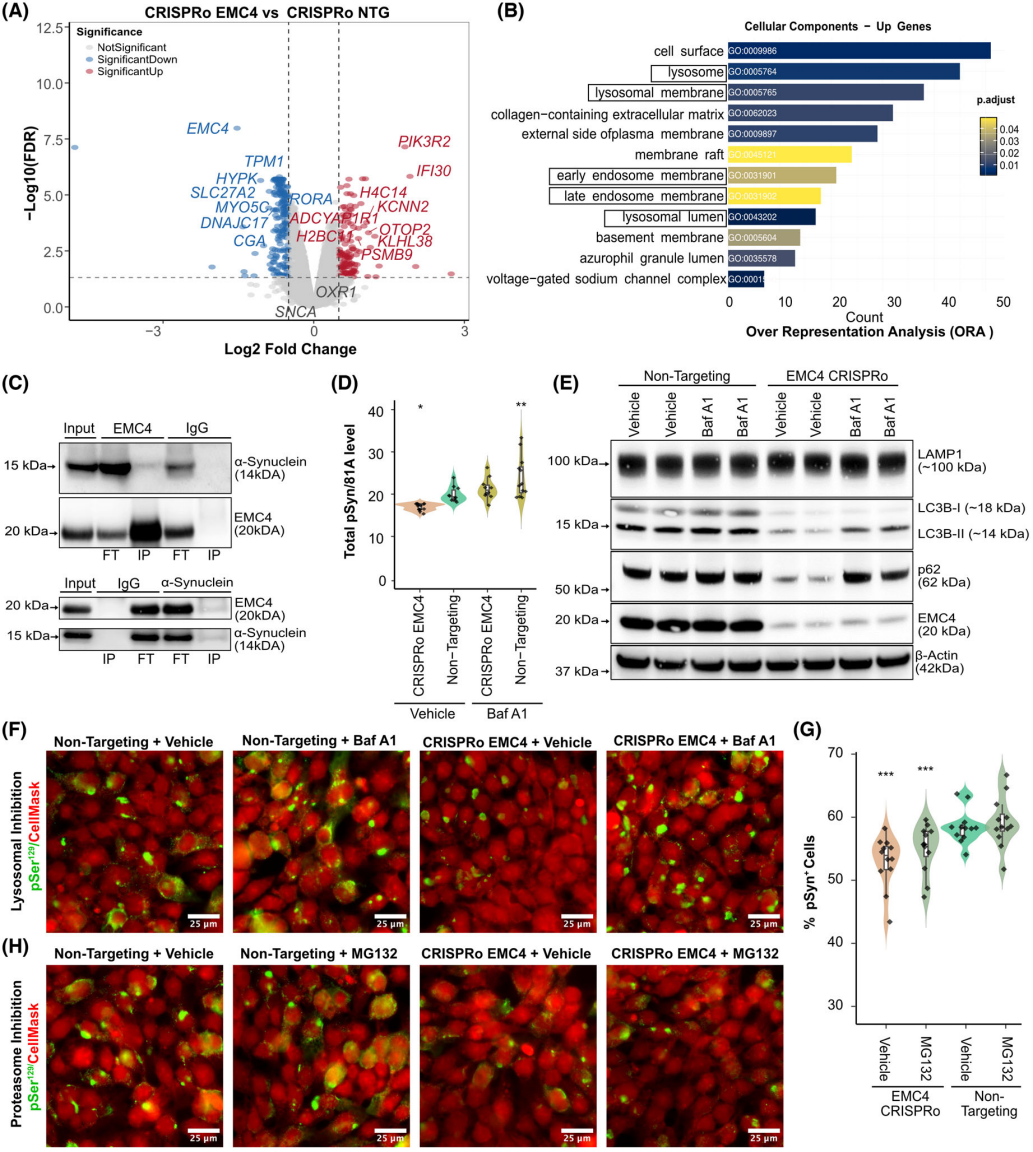
EMC4 ablation and lysosomal function in HEK^Syn^ cells. (A) RNA sequencing showing DEGs between CRISPRo EMC4 and CRISPRo non‐targeting (NTG) controls. (B) Hypergeometric ORA of cellular compartments with FDR ≤0.05 associated with upregulated genes. (C) Bidirectional co‐immunoprecipitation (Co‐IP) of EMC4 and αSynuclein (αSyn). Top: Western blots of EMC4 immunoprecipitates probed for EMC4 or αSyn. Bottom: Reciprocal Co‐IP showing αSyn immunoprecipitation and detection of both EMC4 and αSyn. Input lanes: lysate prior to immunoprecipitation, FT (flow‐through) and IP (immunoprecipitated) fractions. (D) Quantification of lysosomal inhibition (Baf A1) effects on total phosphorylated αSynuclein at Ser129 (pSyn129) spot intensity levels, normalized to the total cell number in CRISPRo EMC4 cells. DMSO was used as a vehicle control. (E) Western blot analysis of lysosomal and autophagy markers, including LAMP1, LC3B‐I/II, and p62, in CRISPRo EMC4 and CRISPRo NTG cells treated with vehicle, Baf A1. (F) Representative immunofluorescence images showing the effect of lysosomal inhibition with Baf A1. (G) Quantification of pSyn129^+^ cells under proteasomal inhibition (MG‐132) in EMC4 ablation and NTG conditions. (H) pSyn129^+^ cells under proteasomal inhibition (MG‐132) in CRISPRo EMC4 and NTG conditions. (Red: HCS CellMask; Green: pSyn129/81A). Scale bar, 25 μm. Inner box plots display the median (center line), the 75th percentile (top edge), and the 25th percentile (bottom edge). One‐way ANOVA followed by Dunnett's *post hoc* test; *P < 0.05, **P < 0.01, ***P < 0.001.

We next sought to clarify the mechanism by which EMC4 ablation reduces pSyn^129^ levels. To identify the downstream mechanisms by which EMC4 ablation decreases pSyn^129^, we sequenced mRNA of EMC4‐ablated HEK^Syn^ cells and compared to that of CRISPRo HEK^Syn^ cells transduced with NTG CRISPR guides. DEGs were selected following the same criteria used for OXR1 upregulation (FDR ≤ 0.05, log_2_FC ≥ 0.5 or ≤ −0.5, *P* ≤ 0.01) (Fig. 6A and Table S4). Several genes were significantly upregulated, including *ADCYAP1R1, H2BC11, H4C14, IFI30, KCNN2, KLHL38, OTOP2, PIK3R2*, and *PSMB9*. Downregulated genes included *CGA, DNAJC17, EMC4, HYPK, MYO5C, RORA, SLC27A2*, and *TPM1*. Notably, neither αSyn mRNA (Fig. 6A) nor αSyn protein levels changed upon EMC4 depletion (Fig. 3E), indicating that EMC4 depletion modulates pSyn^129^ levels independently of endogenous SNCA expression.

Next, we tested the top 8 DEGs (upregulators and downregulators) for their effect on pSyn^129^ accumulation. Most of them were associated with lysosomal or proteostatic pathways. Several candidates influenced pSyn^129^ prevalence upon individual perturbation, with some exhibiting bidirectional effects under opposite perturbations. Notably, overexpression of the lysosomal thiol reductase *IFI30* significantly reduced pSyn^129^ load (Fig. S10A). Thiol reductases help unfold proteins destined for lysosomal degradation. Hence, *IFI30* overexpression may facilitate the disassembly of pSyn^129^ aggregates prior to lysosomal digestion. Additionally, genetic ablation of *DNAJC17*, encoding an essential chaperone, decreased pSyn^129^ accumulation, while its activation promoted pSyn^129^ accumulation (Fig. S10A–D). *DNAJC17* mRNA levels decreased following EMC4 ablation, whereas EMC4 levels remained unaffected by *DNAJC17* depletion, suggesting that *DNAJC17* may act downstream of *EMC4* (Fig. S10E–G). Combined ablation of *EMC4* and *DNAJC17* showed no additive effects, indicating that they may share the same pathway (Fig. S10H).

We next sought to identify broader pathway‐level changes underpinning EMC4's mechanism of action. Hypergeometric over‐representation analysis (ORA) revealed significant enrichment of lysosome‐associated cellular components (lysosome, membrane/lumen), as well as endosomal transport pathways and dysregulation of both early and late endosomal compartments (Fig. 6B). Moreover, we observed upregulation of ER stress‐response pathways (Fig. S11A,B) and genes associated with ER homeostasis including *HSPA5*, *DNAJC3*, *DNAJB9*, and *PIK3R2* (Fig. S11C). Since our transcriptomic profiling highlighted an enrichment of lysosome‐associated processes (Fig. 6B) and given that the EMC complex is also implicated in ER‐associated degradation (ERAD), we hypothesized that EMC4 depletion might reduce pSyn^129^ levels by promoting its degradation via these pathways. To test this, we first inhibited lysosomal activity using Bafilomycin A1 (BafA1), a selective inhibitor of lysosomal acidification. The decrease in pSyn^129^ levels following EMC4 depletion was suppressed by BafA1 treatment (Fig. 6D–F), indicating that the reduction was lysosome‐dependent. EMC4‐depleted HEK^Syn^ cells exhibited reduced levels of LC3B‐II (Fig. 6E), a marker of autophagic flux, and p62/SQSTM1, a cargo receptor for autophagic degradation, consistent with increased lysosomal clearance and turnover. Treatment with BafA1 restored both LC3B‐II and p62 levels. Hence, we speculate that pSyn^129^ reduction upon EMC4 ablation proceeds through an increase in the autophagic flux and enhanced lysosomal degradation. No alterations in LAMP1 levels were observed, suggesting that EMC4 ablation does not broadly impact lysosomal biogenesis. Secondly, to determine whether *EMC4* ablation also enhances proteasomal degradation, we treated cells with the proteasomal inhibitor MG132. Unlike BafA1, MG132 treatment failed to restore pSyn^129^ levels (Fig. 6G,H), indicating that *EMC4* depletion does not promote proteasomal clearance of pSyn^129^. Interestingly, co‐immunoprecipitation (Co‐IP) analysis revealed a physical interaction between EMC4 and αSyn. Immunoprecipitation of EMC4 co‐precipitated αSyn, and vice versa, immunoprecipitation of αSyn enriched EMC4 in the pull‐down fraction (Fig. 6C). Specificity was confirmed using IgG controls, which showed no interaction. These results establish EMC4 as a regulator of lysosome‐mediated aggregated αSyn proteostasis and highlight ER stress–lysosomal crosstalk as a key pathway in synucleinopathies.

### EMC4 depletion reduces pSyn^129^ levels in iPSC‐derived cortical neurons

**Fig. 7:**
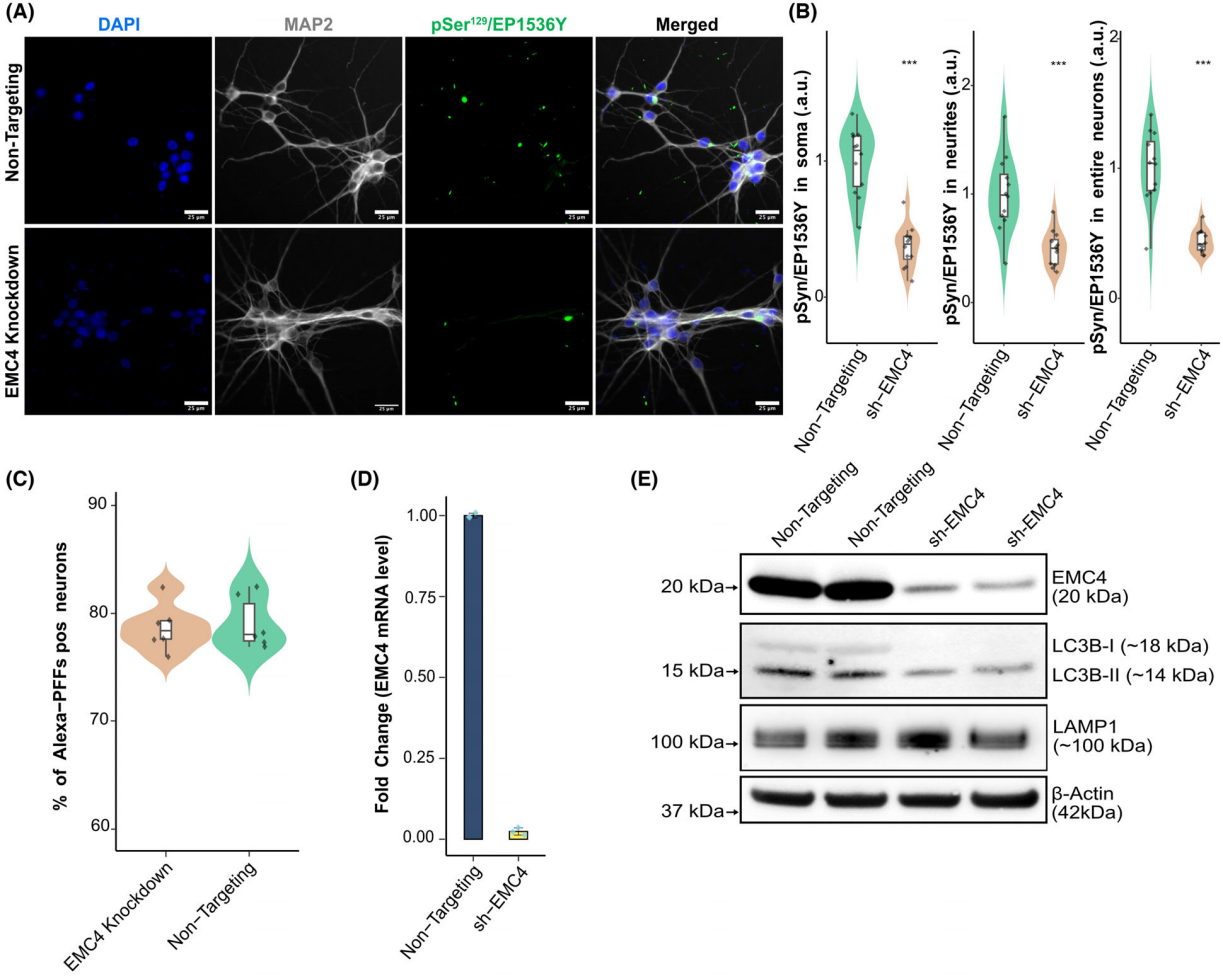
EMC4 depletion modulates phosphorylated αSynuclein at Ser129 (pSyn^129^) levels in human iPSC‐derived cortical neurons. (A) Neurons subjected to EMC4 shRNA or to control conditions. DAPI (blue), MAP2‐labeled neurons (gray), and EP1536Y stained pSyn^129^ aggregates (green). Scale bar, 25 μm. (B) Quantification of pSyn^129^ spot area: (left) in soma, (middle) neurites and (right) whole neurons normalized to total MAP2 area. (C) Flow cytometry analysis of fluorescent PFF uptake by neurons. (D) RT‐qPCR analysis of EMC4 mRNA levels in knockdown neurons. Bar plot data are presented as mean ± SEM. (E) Western blot analysis of lysosomal and autophagy markers in cortical neurons after shRNA‐mediated knockdown of EMC4. Violin plots represent data distribution. Box plots display the median (center line), the 75th percentile (top edge), and the 25th percentile (bottom edge). Welch's *t*‐test (unequal variance *t*‐test), ***P < 0.001.

To investigate the effects of *EMC4* depletion in iPSC‐derived cortical neurons, we used shRNA to deplete its expression (Fig. 7A,B). shRNA‐mediated EMC4 depletion resulted in a significant reduction in pSyn^129^ levels stained with antibody EP1536Y, consistent with the effect observed in HEK cells. No differences were detected in PFF internalization between EMC4‐depleted and scrambled shRNA iPSC‐derived cortical neurons (Figs 7C and S12A), confirming that the reduced pSyn^129^ accumulation cannot be explained by altered PFF uptake. Efficient knockdown of EMC4 was confirmed by RT‐qPCR (Fig. 7D) and by immunoblotting, which demonstrated an ~ 75% reduction in EMC4 protein (Fig. 7E). This reduction in pSyn^129^ was corroborated by 81A immunostaining in iPSC‐derived cortical neurons (Fig. S13A,B). Furthermore, in iPSC‐derived cortical neurons, shRNA‐mediated EMC4 knockdown did not significantly alter neuronal viability as measured by CellTiter‐ Glo (Fig. S13C). Consistent with findings in HEK^Syn^ cells, *EMC4* knockdown in iPSC‐derived cortical neurons also led to reduced LC3B‐II levels, while LAMP1 expression remained unchanged (Fig. 7E), supporting engagement of the autophagy‐lysosome pathway. These data, obtained in disease‐relevant neuronal models, validate the lysosome‐dependent mechanism of pSyn^129^ clearance observed in *EMC4*‐ablated HEK^Syn^ cells.

## Discussion

Human and experimental genetics converge to indicate that αSyn aggregation is a major contributor to PD, MSA, and DLB. Hence, the identification of targetable modifiers of this process may help devise causal treatments for these ailments [14, 68, 69]. Yet few such modifiers are known. We reasoned that large‐scale interrogations of appropriate cellular models of disease may help discover wholly new actors, including those that may not be identifiable by studies of human genetics. We therefore deployed arrayed dual CRISPR activation/ablation screens targeting all genes associated with mitochondrial dynamics and intracellular trafficking, and assessed their impact on pSyn^129^ accumulation, a key hallmark of synucleinopathies.

In contrast to pooled CRISPR screens, image‐based arrayed CRISPR screens provide single‐cell resolution for thousands of cells, enabling the statistically robust detection of subtle phenotypes. However, they can suffer from replicability issues due to imaging artifacts, signal fluctuations and missegmentation during image analysis. Furthermore, despite the high specificity of CRISPR‐based gene perturbations, off‐target effects and false positives remain challenging in large‐scale screens [70]. We mitigated these limitations by using quadruple guides (qgRNA) per gene to ensure targeting redundancy. We also note that some true regulators may be missed (false negatives) if they are not efficiently perturbed in this system (e.g., due to variable guide performance, locus accessibility, or baseline expression), and bandwidth limitations prevented us from measuring expression changes for every screened gene in the primary screen.

We implemented multiple orthogonal validation strategies, robust image analysis pipelines, as well as multiple distinct cell‐based models (HEK293 cells and iPSC‐derived neurons) to ensure biological relevance. HEK293 cells are often described as epithelial, yet they likely arose from neuroendocrine cells [52], express specific neuronal markers, and are responsive to neuronal stressors. Though imperfect, these neuron‐like characteristics, in combination with their rapid cell cycles and ease of culture, make HEK293 cells suitable for genetic screens related to neurological diseases.

To maximize discovery of novel modulators, we designed a broad screen encompassing genes implicated in mitochondrial function, trafficking, cytoskeletal and motor/adaptor pathways (motility), or proteostasis, some of which have been identified as integral to Lewy body and Lewy neurite pathology [13]. This approach identified the mitochondrial *OXR1* and ER‐associated *EMC4* proteins as regulators of pSyn^129^. OXR1 activation exacerbated αSyn pathology via compromised mitochondrial function, whereas EMC4 depletion facilitated αSyn clearance through lysosomal degradation independently of proteasomal activity.

Mitochondrial dysfunction is a hallmark of PD and is linked to the accumulation of pSyn^129^. Recent studies suggest a self‐reinforcing cycle: pSyn^129^ accumulation impairs mitochondrial Complex I and increases ROS, exacerbating pSyn^129^ accumulation [25, 26], while mitochondrial malfunction and ATP depletion promote αSyn misfolding [71].

This bidirectional crosstalk creates a pathological loop, yet the initial trigger—mitochondrial insult or αSyn toxicity—remains unclear [72]. We observed that OXR1 activation coincided with elevated pSyn^129^ levels and was associated with reductions in mitochondrial membrane potential and ATP synthesis. This may impair ATP‐dependent proteostasis mechanisms, such as chaperone‐assisted refolding and proteasome‐mediated degradation of misfolded proteins [73]. As a result, αSyn misfolds and accumulates. This is consistent with prior evidence of mitochondrial dysfunction and ATP deficits exacerbating αSyn pathology [74, 75, 76].

Besides protecting neurons against oxidative stress [65], OXR1 has been implicated in retromer maintenance [77] and epigenetic regulation in neurodevelopment [78], suggesting a broader role in protein homeostasis. *OXR1* expression is disease‐ and context‐dependent: while it is downregulated in PD patient brains [79], it is upregulated in ALS human neuronal models [80], suggesting that its function may shift depending on cellular stress conditions. Importantly, *OXR1* loss in humans is associated with severe neurological defects and premature death [81]. Exosomal miR‐137 downregulates OXR1, worsening oxidative stress in a PD mouse model [82] and underscoring the critical role of OXR1 in neurodevelopment. Thus, while OXR1 activation may enhance neuronal survival at physiological levels, supraphysiological overexpression (as achieved here via CRISPRa) could overwhelm mitochondrial buffering capacity, shifting from protective to detrimental outcomes. These findings suggest that while *OXR1* activation promotes pSyn^129^ accumulation, its neuroprotective roles must be carefully considered in therapeutic strategies, as indiscriminate inhibition could have unintended consequences on neuronal function. While *OXR1* is essential for oxidative stress resistance, we found that its overexpression disrupts mitochondrial homeostasis, suppressing proton motive force genes (*ATP5F1A/B*, *NDUFS2*, *NDUFS4*) and driving pSyn^129^ accumulation. Studies suggest that excessive antioxidant activity can induce reductive stress, which in turn disrupts mitochondrial metabolism and proteostasis [83, 84]. Hence, reductive stress—rather than oxidative stress alone—may contribute to αSyn pathology, potentially through impaired NAD+/NADH homeostasis, ATP depletion, or dysregulated chaperone‐mediated refolding.

In iPSC‐derived cortical neurons, OXR1 activation increased pSyn^129^ burden in both soma and neurites. Similarly, 81A‐stained iDA cultures showed increased pSyn^129^ in both compartments whereas EP1536Y revealed a stronger, statistically significant somatic increase with a similar directional trend in neurites. This compartmental difference may reflect assay sensitivity/variability and the time‐dependent redistribution/maturation of seeded inclusions. These data are consistent with a model in which OXR1‐linked mitochondrial perturbation may preferentially coincide with increased phosphorylation during inclusion maturation/accumulation in the soma, in line with reports that seeded αSyn aggregates can emerge in neurites before redistributing to the soma and accumulating into larger phosphorylated inclusions [13]. Although our PFF‐based model reliably induces pSyn^129^ inclusions, it does not fully capture the complexity of patient‐derived Lewy bodies, which often incorporate lipids and membranous organelles [85, 86]. Nevertheless, seeded PFF aggregates in neuronal culture can partly recapitulate organelle and lipid incorporation, indicating some overlap with the pathology observed in patient brain tissue [13].

Antibody specificity remains a critical consideration in αSyn aggregation studies. While the 81A antibody, a widely used pan‐pSyn^129^ marker, has been reported to cross‐react with phosphorylated neurofilaments in pathological inclusions [87], we observed no detectable 81A signal in untreated control conditions, confirming no off‐target binding in our experimental system. Concordant results obtained with EP1536Y—a selective antibody for pSer^129^ αSyn [88]—strengthen the validity of our findings, as both antibodies consistently reflected αSyn pathology across models. In addition, 81A is being used widely in αSyn aggregation studies and is considered robust when used alongside EP1536Y [89].

Although Ser^129^ phosphorylation dominates PD pathology, it remains unknown whether our identified genetic modifiers also affect other phosphorylation sites (e.g., Tyr125, Ser87, and Tyr39) reported to be critical for αSyn membrane binding [90]. The strain‐specific sensitivity of MSA and fibrillar αSyn strains to OXR1 activation suggests a structure/pathology relationship. Further structural characterization of αSyn strains may clarify this selectivity and provide insights into differential aggregation mechanisms.

MSA‐derived αSyn strains are often reported to exhibit more aggressive prion‐like propagation than PD‐ or DLB‐derived strains [91, 92]. αSyn aggregates extracted from MSA glial cytoplasmic inclusions are conformationally distinct and ~ 1000‐fold more potent than Lewy body–derived αSyn in seeding intracellular αSyn aggregation [92]. However, in our HEK^Syn^ pSyn^129^ assay, the MSA‐derived preparation elicited a lower seeding response than the PD/DLB material, suggesting that strain potency is modulated by the cellular context.

The EMC complex maintains ER homeostasis, and its malfunction leads to the accumulation of misfolded proteins and unfolded protein response (UPR) activation [93]. Aberrantly folded proteins are typically cleared through ERAD or ER‐to‐lysosome‐associated degradation (ERLAD) [94]. Notably, while EMC4 plays an important role in EMC functionality, its depletion does not compromise other EMC subunits' stability or abundance, indicating that EMC4 is not structurally essential for the complex [93]. In PD, αSyn aggregates localize to the ER in both human postmortem brains and mouse models [95]. αSyn also interacts with ER chaperones, leading to impaired ER‐associated proteostasis [96]. The finding that *EMC4* ablation reduces pSyn^129^ burden via enhanced lysosomal degradation reinforces the emerging role of ER‐lysosome crosstalk in αSyn clearance and offers a mechanistic bypass for ERAD‐compromised neurons in synucleinopathies. These findings align with recent reports demonstrating the involvement of lysosomal degradation [97] and ER‐phagy, a selective form of autophagy that degrades ER components to maintain ER homeostasis [98] in αSyn aggregate removal. They are further supported by evidence that enhancing ER proteostasis and protein trafficking in patient‐derived neurons can synergistically reduce αSyn pathology [99]. Lysosomal dysfunction is intimately linked to αSyn pathology, as exemplified by *GBA1*, which encodes the lysosomal enzyme glucocerebrosidase and constitutes one of the strongest genetic risk factors for Parkinson's disease [100]. Loss‐of‐function mutations in *GBA1* impair lysosomal clearance of αSyn, thereby exacerbating its aggregation and toxicity. Although *GBA1* was not included in our sublibrary, our identification of *EMC4* as a lysosomal regulator underscores that multiple, potentially convergent pathways can influence αSyn proteostasis.

We found that *EMC4* depletion downregulates *DNAJC17*, a proteostasis‐associated chaperone, and its ablation reduces pSyn^129+^ prevalence. While *DNAJC17* has been implicated in nuclear mRNA processing and splicing [101], its direct role in pSyn^129^ clearance whether as a mediator of lysosomal degradation or an epiphenomenon, remains unresolved. While our findings highlight EMC4 as a potent regulator of αSyn pathology, existing large‐scale transcriptomic and proteomic studies in PD and MSA do not explicitly report EMC4 dysregulation. However, this does not exclude the possibility of subtle or region‐specific changes in EMC4 expression or function that may fall below detection thresholds in bulk analyses.

In conclusion, our work defines mitochondrial OXR1 (activation‐driven enhancer) and ER‐associated EMC4 (ablation‐dependent suppressor) as important regulators of αSyn proteostasis. The polygenic and multifactorial nature of synucleinopathies demands a shift toward combinatorial strategies targeting mitochondrial resilience, ER‐lysosome coordination, and post‐translational modification networks, some of which are being identified in the current study. By leveraging advanced models such as patient‐derived fibrillar strains, human iPSC‐derived neurons, and CRISPR tools to target these pathways, we may disrupt self‐reinforcing cycles of proteostatic collapse in Parkinson's disease and possibly in other synucleinopathies.

## Conflict of interest

The authors declare no conflict of interest.

## Author contributions

SN designed, performed or contributed to all experiments, analyzed data (including statistical, image analyses, data visualization), and wrote the manuscript. LN performed co‐immunoprecipitation (Co‐IP) experiments, and αSyn uptake assays in iPSC‐derived neurons; assisted in cell culture, immunostaining and imaging. LM contributed to intracellular flow cytometry‐based screening of RNA‐seq DEGs, lentivirus production, CTG viability assays, and western blotting. TG designed dopaminergic neuron experiments, performed assays in dopaminergic neuronal models, and provided technical expertise. NK assisted in imaging and analysis of induced dopaminergic (iDA) cultures. SS maintained iPSC cultures and assisted in differentiation protocols. VB advised on flow cytometry experimental design and data interpretation. J‐AY provided CRISPR guide RNAs and CRISPR‐related technical guidance. RM prepared distinct αSynuclein (αSyn) fibril strains and gave inputs on fibrillar strain.

## Supporting information


**Fig. S1.** High‐content CRISPRa and CRISPRo αSyn aggregation assay validation and workflow optimization.
**Fig. S2.** Development of image analysis pipeline, data quality assessment, and formal validation of key hits in HEK^Syn^ cell lines.
**Fig. S3.** Toxicity assessment and representative immunofluorescence images of HEK^Syn^ cells showing phosphorylated αSynuclein at Ser129 (pSyn^129^) aggregates.
**Fig. S4.** Gating strategy for flow cytometry and measurement of phosphorylated αSynuclein at Ser^129^ (pSyn^129^) positive cells.
**Fig. S5.** Effects of CRISPR activation (CRISPRa) /CRISPR ablation (CRISPRo) perturbations on phosphorylated αSynuclein at Ser^129^ (pSyn^129^) levels in response to αSynuclein polymorphs.
**Fig. S6.** Flow cytometry gating strategy and intersection analysis of differentially expressed genes (DEGs) modulating phosphorylated αSynuclein at Ser129 (pSyn^129^).
**Fig. S7.** Pathway enrichment analysis, measurement of MitoSOX‐based superoxide levels and TMRM‐based mitochondrial membrane potential using live‐cell imaging.
**Fig. S8.** Generation and analysis of human iPSC‐derived cortical neurons.
**Fig. S9.** Assessment of αSynuclein aggregate levels in iPSC‐derived dopaminergic (iDA) neurons.
**Fig. S10.** Transcriptomic changes and functional effects of downstream genes on phosphorylated αSynuclein at Ser^129^ (pSyn^129^).
**Fig. S11.** Pathway enrichment analysis in *EMC4* ablated HEK^Syn^ cells.
**Fig. S12.** Gating strategy for quantifying αSyn PFF uptake by flow cytometry.
**Fig. S13.** Assessment of αSynuclein aggregate levels and toxicity in iPSC‐derived cortical neurons upon EMC4 knockdown.

**Table S1.** Excel spreadsheet listing CRISPRa screen data (genename, pvalue, log_2_FC, Cell number threshold).

**Table S2.** Excel spreadsheet listing CRISPRo screen data (genename, pvalue, log_2_FC, Cell number threshold).

**Table S3.** Excel spreadsheet of RNAseq data (NTG vs OXR1).

**Table S4.** Excel spreadsheet of RNAseq data (EMC4 vs OXR1).

**Table S5.** Excel spreadsheet listing iDA dopaminergic neurons media components.

**Table S6.** Excel spreadsheet listing antibodies application and dilution.

**Table S7.** Excel file containing qPCR Primers sequence.

**Data S1.** Uncut western blot.

**Data S2.** Excel File Source Data.

## Data Availability

All data supporting the findings of this study are included in this published article and its Supporting Information files. Raw microscopy images generated during this study have been deposited in FigShare (https://figshare.com/s/ce7273dfd93096f9c01e; will be made public upon publication) and Zenodo (DOI: 10.5281/zenodo.15358052). Bulk RNA sequencing data will be available through the Gene Expression Omnibus (GEO) under accession number GSE295558 upon publication, in accordance with NIH data sharing policies. Source data are provided with this paper. This study did not generate novel computational code. Data analysis and visualization (e.g., statistical tests, violin plots, volcano plots) were performed using standard functions in R (version 4.3.1) with publicly available packages (e.g., ggplot2, dplyr). The CellProfiler pipeline used to analyze pSyn^129^ staining in HEK cells, neurites, and soma is available from the corresponding author upon reasonable request.
