# Supplementary material for: Large‐scale bidirectional arrayed genetic screens identify OXR1 and EMC4 as modifiers of αSynuclein aggregation
Source: FEBS Open Bio. 2026 Mar 30;16(8):1563–92. doi: 10.1002/2211-5463.70233 (PMC13398675; doi:10.1002/2211-5463.70233)
Supplement: Supplementary file 1 — Fig. S1. High‐content CRISPRa and CRISPRo αSyn aggregation assay validation and workflow optimization. Fig. S2. Development of image analysis pipeline, data quality assessment, and formal validation of key hits in HEKSyn cell lines. Fig. S3. Toxicity assessment and representative immunofluorescence images of HEKSyn cells showing phosphorylated αSynuclein at Ser129 (pSyn129) aggregates. Fig. S4. Gating strategy for flow cytometry and measurement of phosphorylated αSynuclein at Ser129 (pSyn129) positive cells. Fig. S5. Effects of CRISPR activation (CRISPRa) /CRISPR ablation (CRISPRo) perturbations on phosphorylated αSynuclein at Ser129 (pSyn129) levels in response to αSynuclein polymorphs. Fig. S6. Flow cytometry gating strategy and intersection analysis of differentially expressed genes (DEGs) modulating phosphorylated αSynuclein at Ser129 (pSyn129). Fig. S7. Pathway enrichment analysis, measurement of MitoSOX‐based superoxide levels and TMRM‐based mitochondrial membrane potential using live‐cell imaging. Fig. S8. Generation and analysis of human iPSC‐derived cortical neurons. Fig. S9. Assessment of αSynuclein aggregate levels in iPSC‐derived dopaminergic (iDA) neurons. Fig. S10. Transcriptomic changes and functional effects of downstream genes on phosphorylated αSynuclein at Ser129 (pSyn129). Fig. S11. Pathway enrichment analysis in EMC4 ablated HEKSyn cells. Fig. S12. Gating strategy for quantifying αSyn PFF uptake by flow cytometry. Fig. S13. Assessment of αSynuclein aggregate levels and toxicity in iPSC‐derived cortical neurons upon EMC4 knockdown. [file FEB4-16-1563-s009.docx]

**Supplementary Figures S1-S13**

**
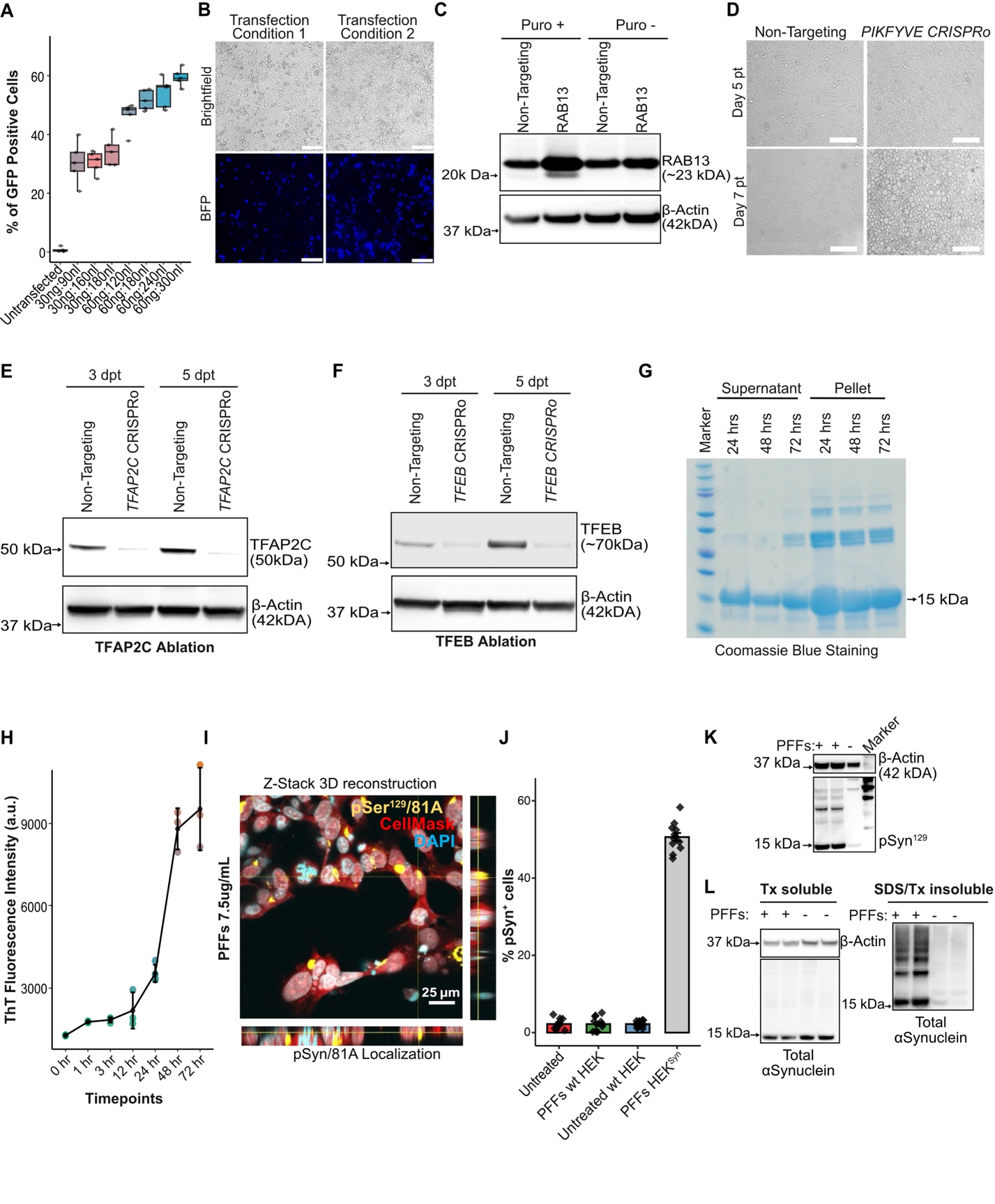
**

**Figure S1: High-content CRISPRa and CRISPRo αSyn aggregation assay validation and workflow optimization.** **(A)** Transfection efficiency optimization in a 384-well plate using different ratios of plasmid DNA concentration to the transfection reagent ViaFect. Box plots display the median (centre line), the 75th percentile (top edge), and the 25th percentile (bottom edge). **(B)** Representative micrographs showing brightfield (BF) and blue fluorescent protein (BFP) under two transfection conditions: (Left) Day 1 seeding at 5000 cells followed by transfection on Day 2; (Right) Day 1 seeding at 3000 cells followed by transfection on Day 3. Scale bar: 200 µm. **(C)** Western blot showing dCas9 activity in RAB13 CRISPRa guide-transfected cells under puromycin selection (+Puro) and without selection (-Puro). **(D)** Brightfield images showing *PIKFYVE* ablation inducing extensive vacuolation in CRISPRo cells. Scale bar: 200 µm. pt, post transfection. **(E, F)** Western blot analysis of TFAP2C and TFEB ablation efficiency in CRISPRo HEK293 cells at 3 and 5 days post-transfection (dpt). **(G)** Coomassie-stained gel showing progressive fibrillation of αSyn over 72 hours. **(H)** Thioflavin T (ThT) assay to measure αSyn fibrillation kinetics. Data are presented as mean ± SEM. **(I)** In vitro assessment of αSyn PFF transduction using the transfection reagent Mirus in HEK293 cells, with 3D Z-stack reconstruction showing phosphorylated αSynuclein at Ser^129^ /81A localisation. CellMask (red), pSyn^129^ detected using the 81A (yellow), and DAPI-stained nuclei (blue). Scale bar, 25 µm. **(J)** Quantification of pSyn^129+^ cells under different experimental conditions: PFF-treated wild-type HEK cells (wt HEK; HEK293T cells with no or very low αSyn expression) and PFF-treated HEK293 cells overexpressing αSyn. Bars show mean ± s.e.m. **(K)** Representative immunoblot of HEK^Syn^ cells treated with αSyn PFFs, probed for pSyn^129^ using the D1R1 anti-pSyn antibody. **(L)** Biochemical fractionation of HEK^Syn^ cells treated with αSyn PFFs, followed by immunoblotting for total αSyn in the Triton X-100 (Tx)-soluble fraction (left) and the SDS/Tx-insoluble fraction (right). Data: mean ± SEM.


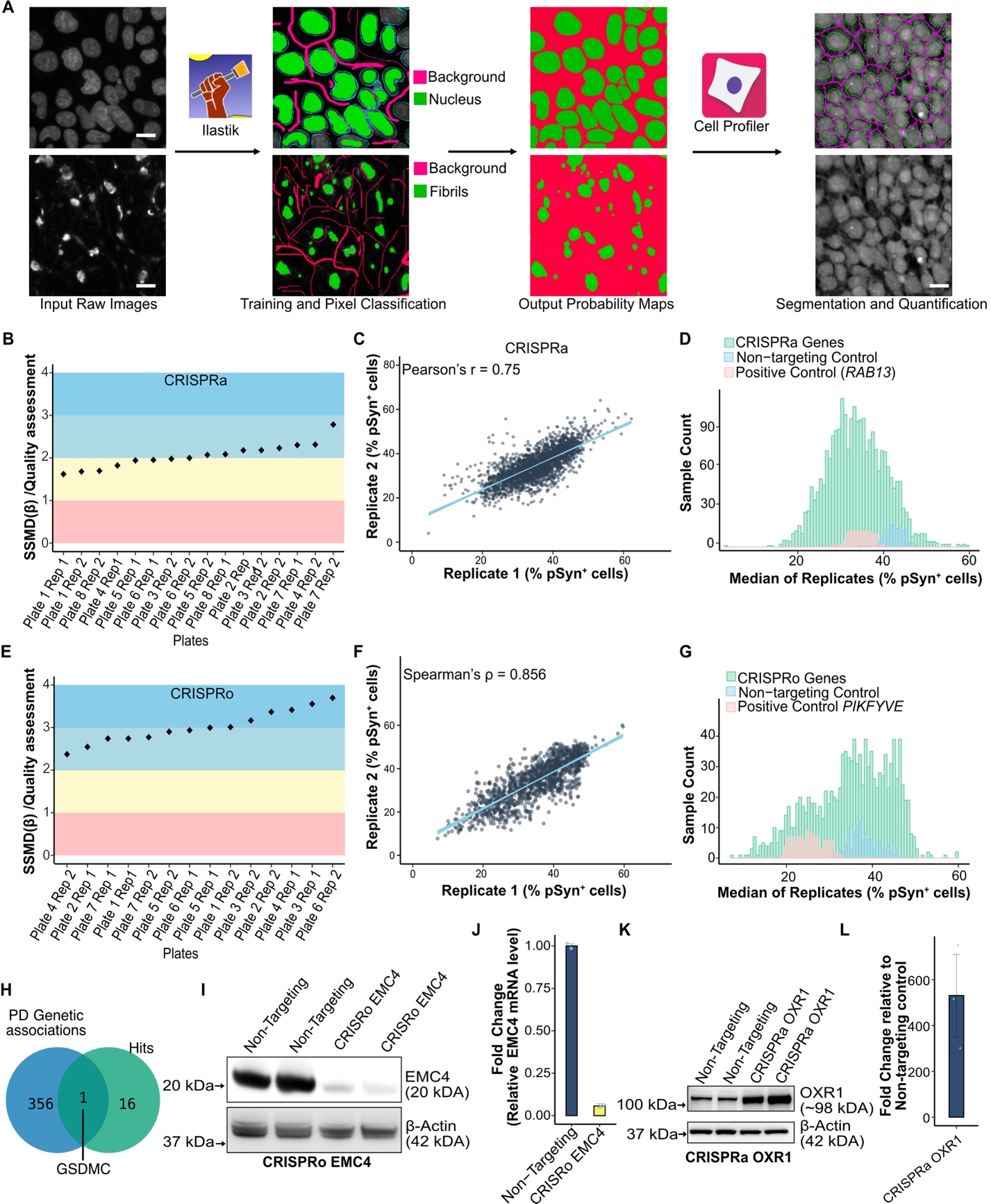


**Figure S2: Development of image analysis pipeline, data quality assessment, and formal validation of key hits in HEK^Syn^ cell lines.** **(A)** Workflow for segmentation and quantification using ilastik for pixel classification (nuclei: pink; fibrils: green; background: red) and CellProfiler for segmentation and quantification. Scale bar: 10 µm. (**B, E)** Strictly standardised mean difference (SSMD) scores assessing the quality of individual CRISPRa (**B)** and CRISPRo (**E)** plates based on non-targeting and positive controls. **(C, F)** Scatter plots showing correlation between duplicates of the primary CRISPRa (Pearson’s correlation) **(C)** and CRISPRo **(F)** (Spearman’s correlation) screens. (**D, G)** Histograms showing the frequency distribution of individual CRISPRa **(D)** and CRISPRo **(G)** screens, including non-targeting and positive controls: *RAB13* for CRISPRa and *PIKFYVE* for CRISPRo. **(H)** Venn diagram showing the overlap of validated CRISPRa and CRISPRo hits with Parkinson's disease genetic associations from the Open Targets platform. **(I)** Western blot analysis of EMC4 protein levels in non-targeting and CRISPRo *EMC4* ablation conditions. **(J)** RT-qPCR showing the relative mRNA levels of *EMC4* in CRISPRo *EMC4* ablation cells. **(K)** Western blot analysis of OXR1 protein levels in CRISPRa *OXR1*-activated cells. **(L)** RT-qPCR showing the relative mRNA levels of *OXR1* in CRISPRa *OXR1*-activated cells. Bar plots data are presented as mean ± SEM.


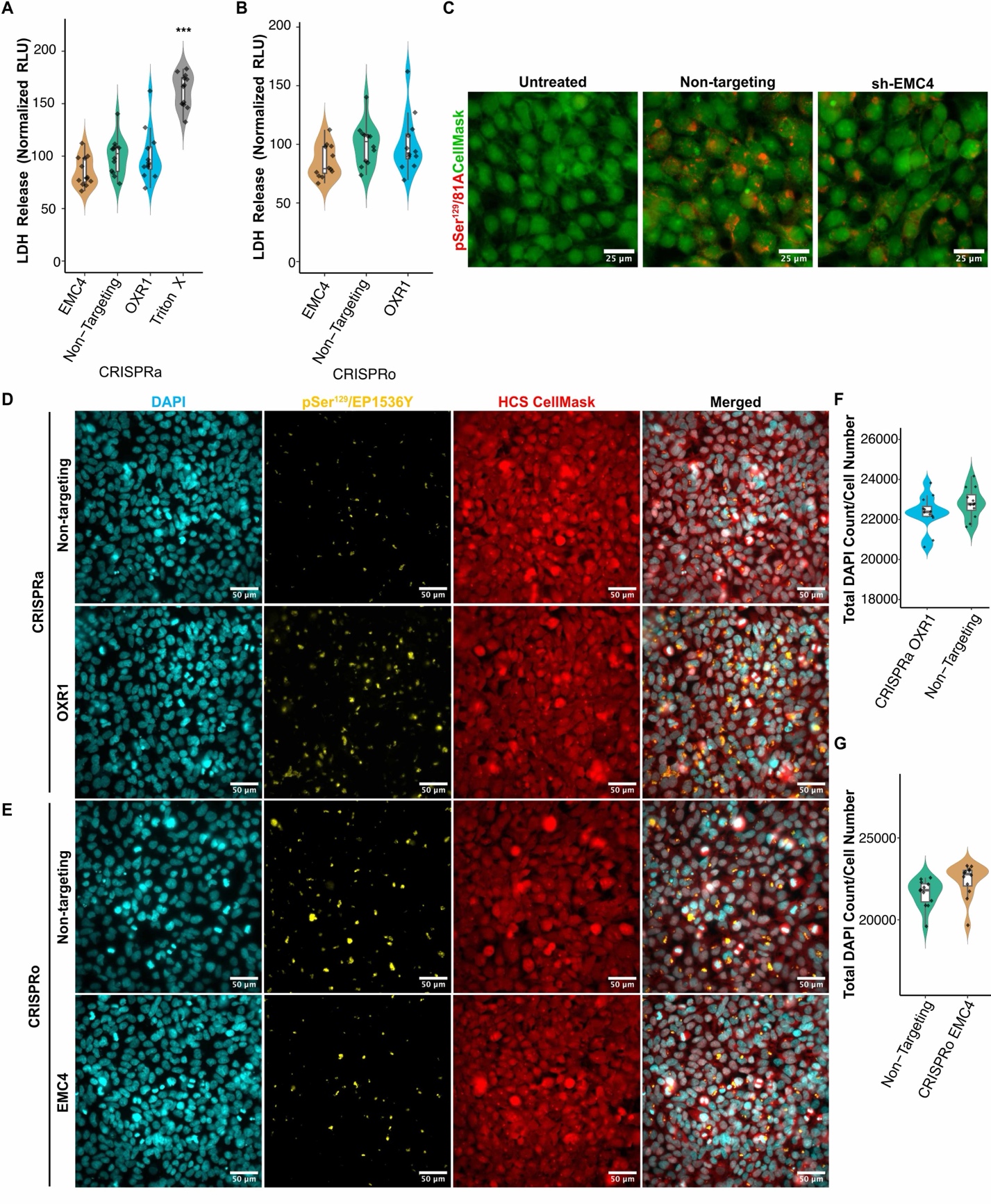


**Figure S3:** **Toxicity assessment and representative immunofluorescence images of HEK^Syn^ cells showing phosphorylated αSynuclein at Ser129 (pSyn^129^) aggregates.** **(A,B)** Measurement of lactate dehydrogenase (LDH) release as a marker of cytotoxicity, expressed as relative luminescence units (RLU) and normalised to the non-targeting control. Cells treated with Triton® X-100 served as a positive control. Statistical comparisons were performed using one-way ANOVA followed by Dunnett's post hoc test, with significance levels denoted as follows: *P < 0.05, **P < 0.01, ***P < 0.001. **(C)** Representative micrographs showing p-Ser^129^ αSyn aggregates detected using the 81A antibody (red), and whole-cell staining with HCS CellMask (green) in HEK^Syn^ cell lines. Scale bar, 25 µm. **(D,E)** DAPI-stained nuclei (cyan), pSyn^129^ aggregates detected with the EP1536Y antibody (yellow), and whole-cell staining with HCS CellMask (red). Rows represent different experimental conditions. CRISPR activation **(D)** and CRISPR ablation **(E).** Scale bar, 50 µm. **(F,G)** Total DAPI-positive nuclei counts used as a proxy for cell number following OXR1 activation **(F)** and EMC4 ablation **(G),** relative to non-targeting controls. Violin plots represent data distribution. Inner box plots display the median (centre line), the 75th percentile (top edge), and the 25th percentile (bottom edge). Statistical comparisons were performed using Welch’s t-test (unequal variance t-test) with significance levels denoted as follows: *P < 0.05, **P < 0.01, ***P < 0.001.


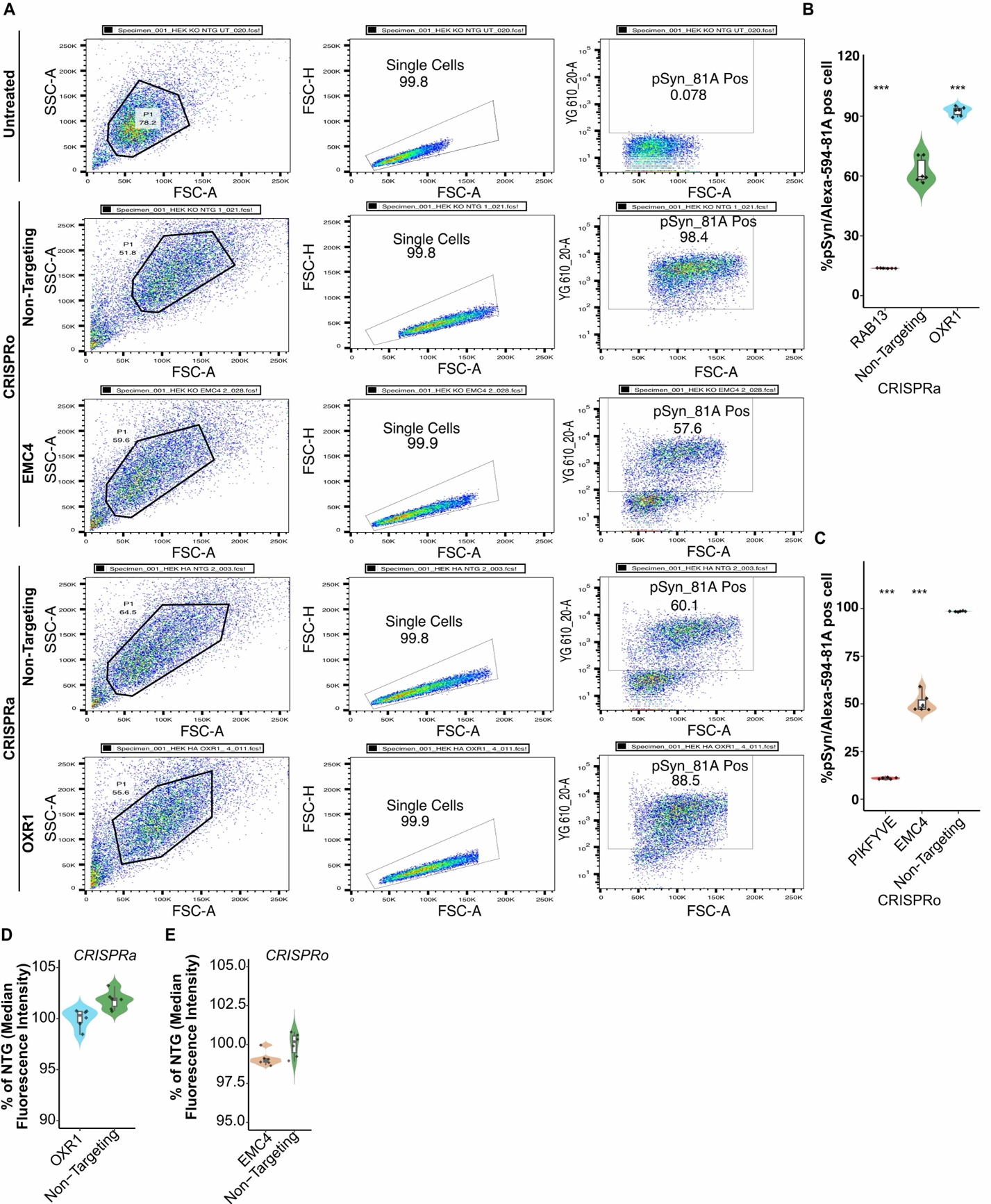


**Figure S4:** **Gating strategy for flow cytometry and measurement of phosphorylated αSynuclein at Ser^129^ (pSyn^129^) positive cells.** **(A)** Gating strategy for CRISPRo hits (upper panel) and for CRISPRa (lower panel) in HEKSyn cell lines. Representation of flow cytometry plots displaying single cell population and the pSyn^129^ (Alexa Fluor 594-labelled 81A antibody) positive cells. **(B)** Percentage of p-Ser^129^-positive cells across CRISPRa hits, based on flow cytometry analysis using the Alexa Fluor 594 81A antibody. **(C)** Percentage of p-Ser^129^-positive cells across CRISPRo hits, based on flow cytometry analysis using the Alexa Fluor 594 81A antibody. Statistical comparisons were performed using one-way ANOVA followed by Dunnett's post hoc test, with significance levels denoted as follows: *P < 0.05, **P < 0.01, ***P < 0.001. **(D,E)** Flow cytometry quantification of Alexa Fluor™ 594 dextran (10 kDa) uptake in CRISPRa OXR1 **(D)** and CRISPRo EMC4 **(E)** lines, expressed as percentage of non-targeting (NTG) median fluorescence intensity. Violin plots represent data distribution. Inner box plots display the median (centre line), the 75th percentile (top edge), and the 25th percentile (bottom edge). Statistical comparisons were performed using Welch’s t-test (unequal variance t-test) with significance levels denoted as follows: *P < 0.05, **P < 0.01, ***P < 0.001.


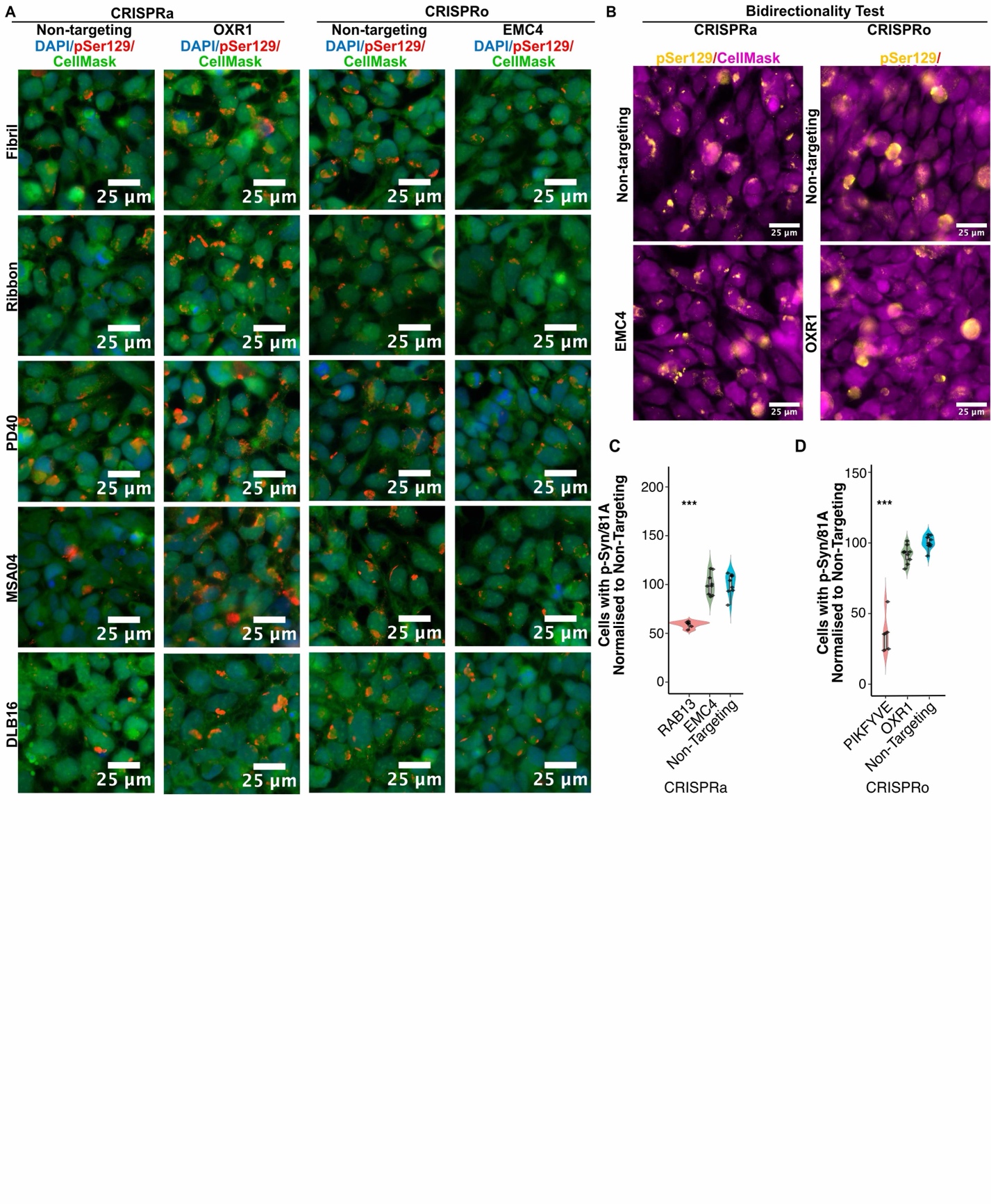


**Figure S5: Effects of CRISPR activation (CRISPRa) /CRISPR ablation (CRISPRo) perturbations on phosphorylated αSynuclein at Ser^129^ (pSyn^129^) levels in response to αSynuclein polymorphs.** **(A)** Representative immunofluorescence images of HEK^Syn^ cells showing phosphorylated αSynuclein at Ser^129^ (pSyn^129^) levels in response to human PD, DLB, and MSA patient-derived fibrils, as well as two distinct recombinant polymorphs: fibrils and ribbons strains. (Cyan: DAPI; Green: HCS CellMask; Red: pSyn^129^/81A). Scale bar, 25 µm. **(B)** Representative images illustrating the effect of hit gene perturbations (bi-directional effects) on pSyn^129^ levels using 81A antibody. (Magenta: HCS CellMask; Yellow: pSyn^129^/81A). Left column: CRISPRa; Right column: CRISPRo. Scale bar, 25 µm. **(C)** Effect of *EMC4* activation on pSyn^129^ levels. **(D)** Effect on pSyn^129^ levels following CRISPRo-mediated ablation of *OXR1*. Statistical comparisons were performed using one-way ANOVA followed by Dunnett's post hoc test, with significance levels denoted as follows: *P < 0.05, **P < 0.01, ***P < 0.001. Violin plots represent data distribution. Inner box plots display the median (centre line), the 75th percentile (top edge), and the 25th percentile (bottom edge).


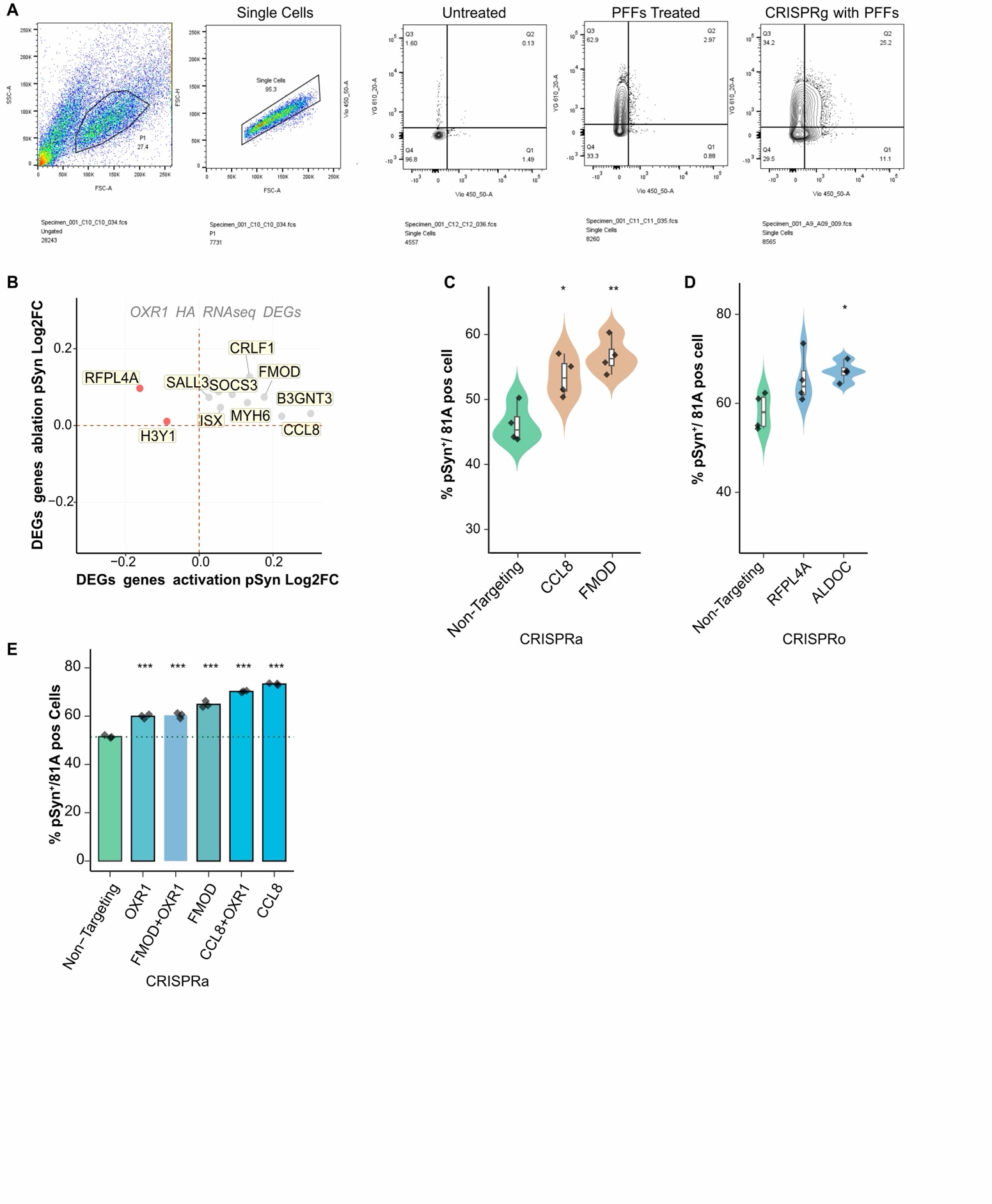


**Figure S6:** **Flow cytometry gating strategy and intersection analysis of differentially expressed genes (DEGs) modulating phosphorylated αSynuclein at Ser129 (pSyn^129^).** **(A)** Flow cytometry gating strategy for the mini-screen of DEGs, including OXR1 activation and EMC4 ablation conditions in HEK^Syn^ cell lines. **(B)** Intersection of log2 fold changes in pSyn^129^ levels for individual perturbed DEGs upon OXR1 activation. **(C-D)** Effect of OXR1 activation DEGs on pSyn^129^ assessed with immunofluorescence imaging. **(E)** Flow cytometry quantification of pSyn^129^ accumulation following single or combined CRISPRa activation of OXR1 with FMOD or CCL8. Bar plots data are presented as mean ± SEM. Violin plots represent data distribution. Inner box plots display the median (centre line), the 75th percentile (top edge), and the 25th percentile (bottom edge). One-way ANOVA followed by Dunnett's post hoc test; *P < 0.05, **P < 0.01, ***P < 0.001.


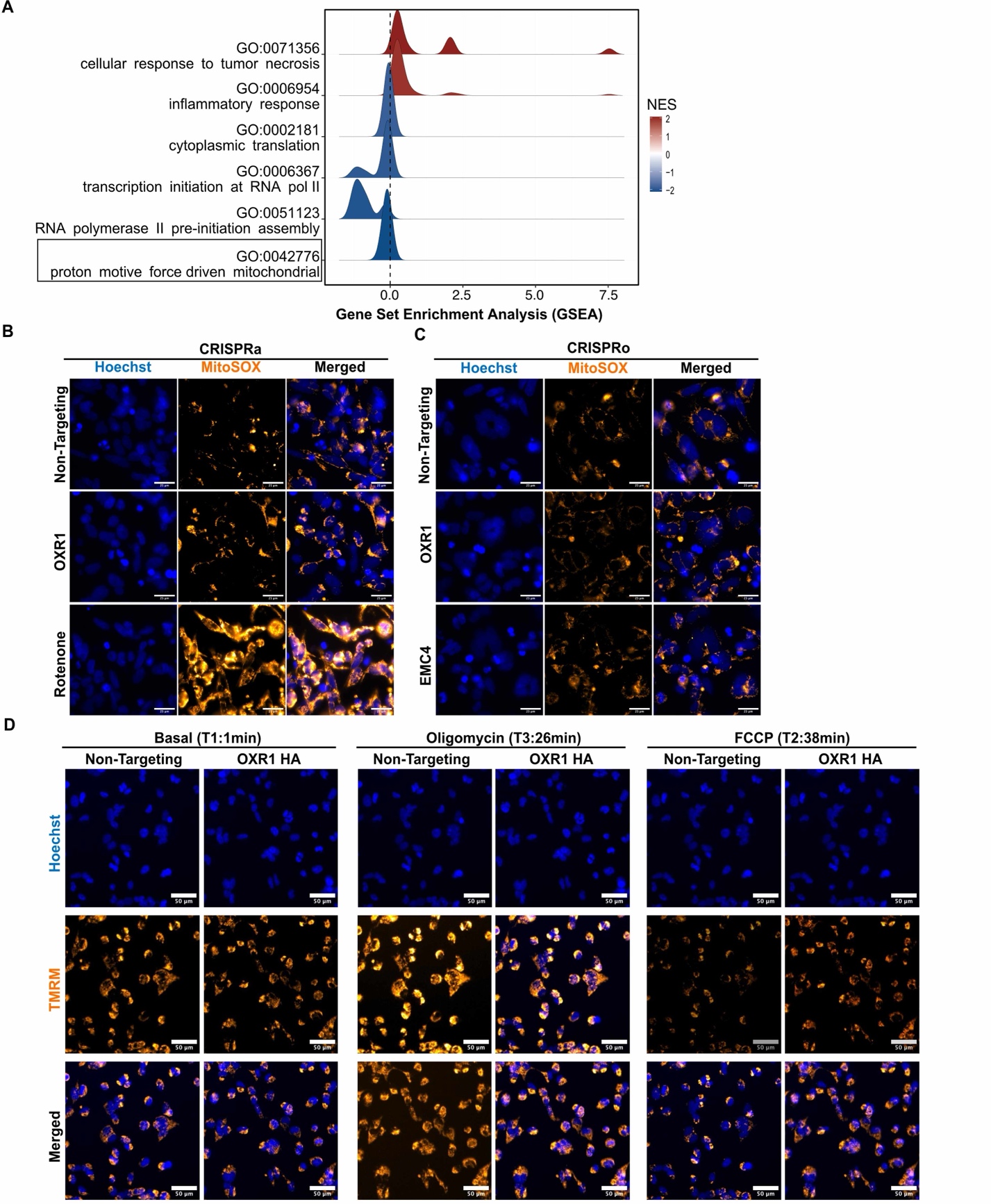


**Figure S7: Pathway enrichment analysis, measurement of MitoSOX-based superoxide levels and TMRM-based mitochondrial membrane potential using live-cell imaging. (A)** Gene set enrichment analysis (GSEA) shows pathways ranked by NES and highlights mitochondria-related pathways. Candidate terms were filtered based on False discovery rate (FDR) ≤ 0.05, and genes were ranked by log2 fold change. **(B)** Representative immunofluorescence images showing mitochondrial superoxide levels stained with MitoSOX™ Red dye upon activation of the hits in HEK^Syn^ cell lines. (Hoechst-stained nuclei: Blue; Orange-hot: MitoSOX™ Red). Rotenone-treated conditions served as the positive control. Scale bar: 25 μm. **(C)** Representative fluorescence images showing mitochondrial superoxide levels stained with MitoSOX™ Red dye upon ablation of the hits in HEK^Syn^ cell lines. (Hoechst-stained nuclei: Blue; Orange-hot: MitoSOX™ Red). Scale bar: 25 μm. (D) Representative immunofluorescence microscopy images showing mitochondrial membrane potential. (Hoechst-stained nuclei: Blue; Orange-hot: TMRM). Images shown were taken at basal conditions (1 min) and following treatment with oligomycin (26 min) and FCCP (38 min). ~1,000 cells across technical replicates were analysed per condition. Scale bar, 25 µm.


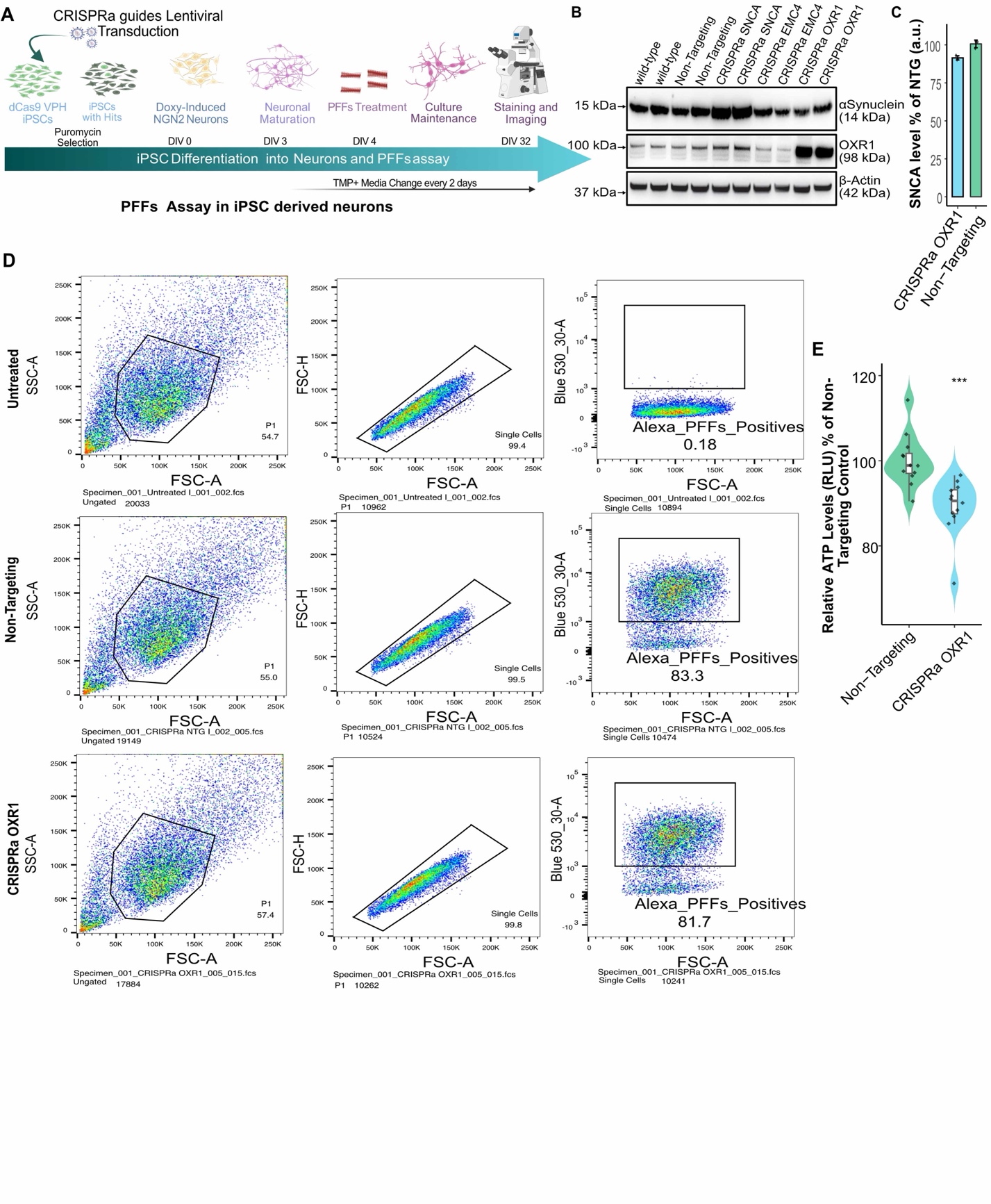


**Figure S8: Generation and analysis of human iPSC-derived cortical neurons.** **(A)** Schematic of iPSC line generation, differentiation, maturation, and PFF treatment in iPSC-derived cortical neurons. **(B)** Representative immunoblot of total αSyn and OXR1 in iPSC-derived cortical neurons following CRISPRa targeting. **(C)** Quantification of SNCA levels normalised to β-actin, shown as percentage of the non-targeting (NTG) control. Bars show mean ± s.e.m. **(D)** Gating strategy for flow cytometry plots displaying single-cell populations and Alexa Fluor 488-positive cells. **(E)** Intracellular ATP levels measured by CellTiter-Glo (RLU: relative luminescence units) and expressed as percentage of the non-targeting control, in iPSC-derived neurons upon OXR1 activation. Violin plots represent data distribution. Inner box plots display the median (centre line), the 75th percentile (top edge), and the 25th percentile (bottom edge). Statistical comparisons were performed using Welch’s t-test (unequal variance t-test) with significance levels denoted as follows: *P < 0.05, **P < 0.01, ***P < 0.001.


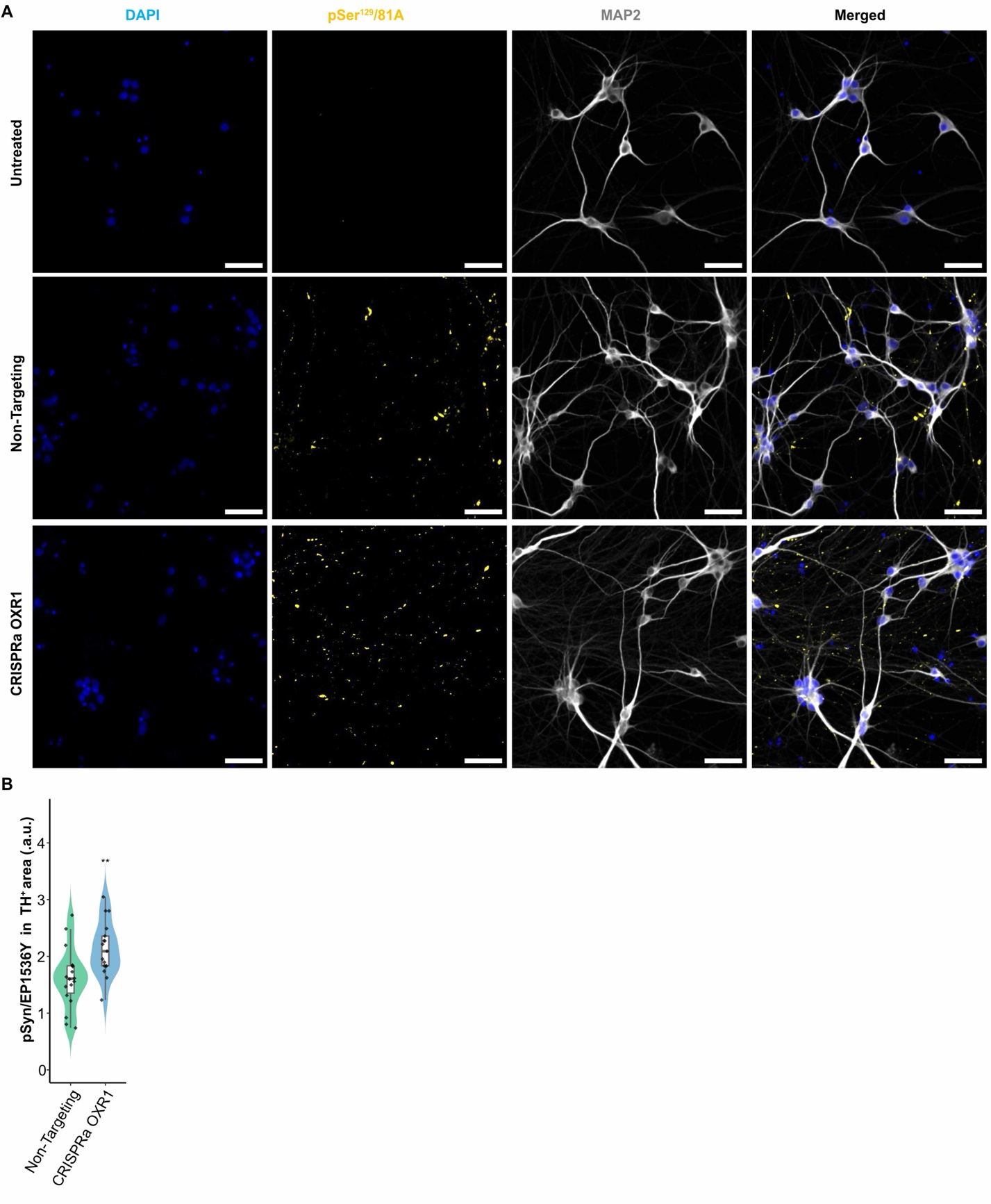


**Figure S9. Assessment of αSynuclein aggregate levels in iPSC-derived dopaminergic (iDA) neurons.** **(A)** Representative immunofluorescence images of human iPSC-derived dopaminergic neurons treated with CRISPRa targeting OXR1 or non-targeting (NTG) controls. iPSC-derived dopaminergic neurons were stained for nuclei (DAPI, blue), neuronal marker MAP2 (grey), and phosphorylated αSynuclein at Ser^129^ (pSyn^129^, detected via 81A antibody, yellow). **(B)** Quantification of pSyn^129^ spot area within TH-positive regions in CRISPRa OXR1 iDA neurons, normalised to TH^+^ area. Scale bar: 25 µm. Violin plots represent data distribution. Inner box plots display the median (centre line), the 75th percentile (top edge), and the 25th percentile (bottom edge). Statistical comparisons were performed using Welch’s t-test (unequal variance t-test) with significance levels denoted as follows: *P < 0.05, **P < 0.01, ***P < 0.001.

**
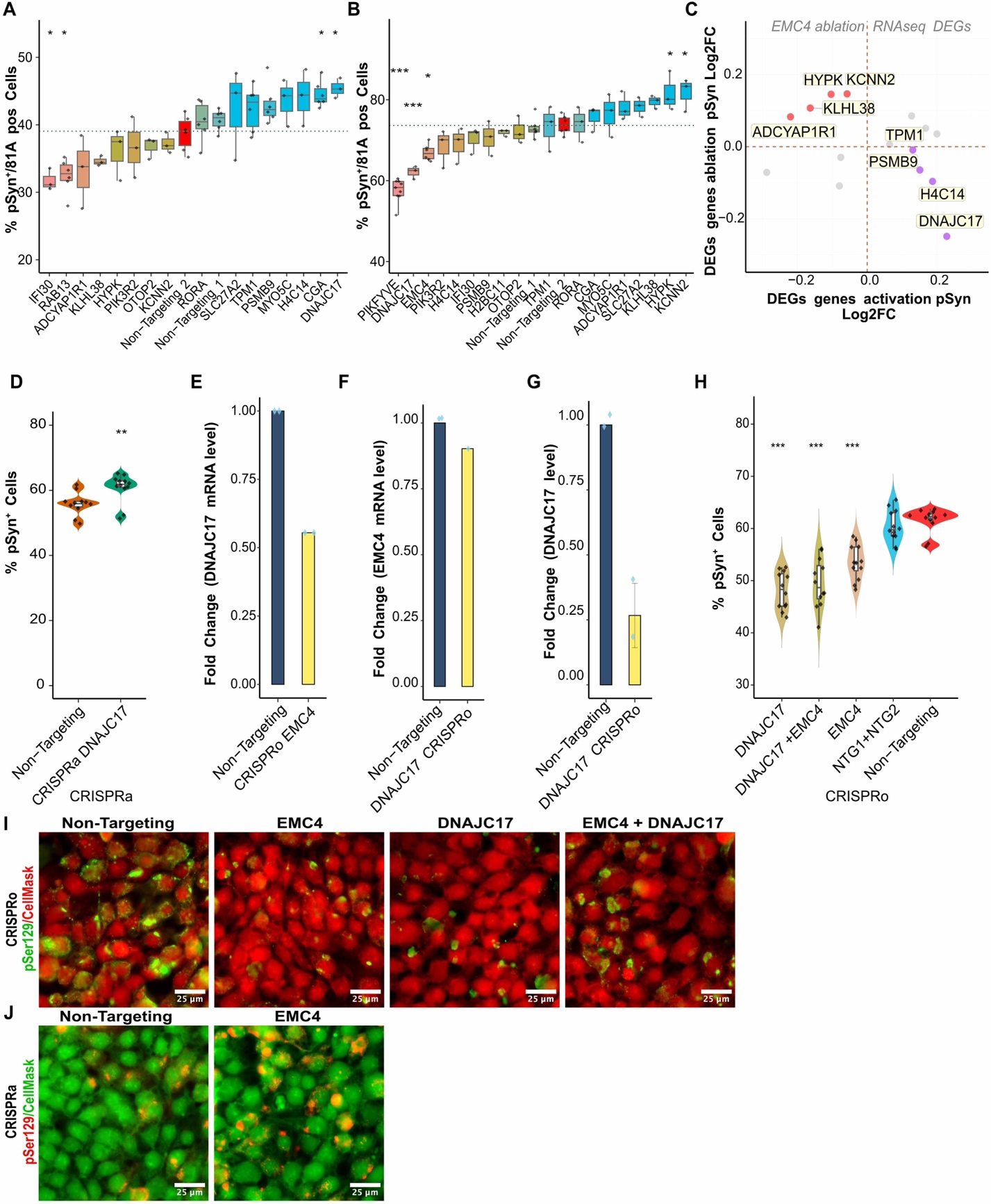
**

**Figure S10:** **Transcriptomic changes and functional effects of downstream genes on phosphorylated αSynuclein at Ser^129^ (pSyn^129^). (A-B)** Individual perturbation of DEGs upon EMC4 ablation in HEK^Syn^ cell lines. Flow cytometry-based percentage of pSyn^129+^cells following CRISPR activation (**A**) and CRISPR ablation (**B**). **(C)** Intersection of log2 fold changes in pSyn^129^ levels for individual perturbed DEGs upon EMC4 ablation. **(D)** Effect of *DNAJC17* activation on pSyn^129^ levels. Statistical comparisons were performed using Welch’s t-test (unequal variance t-test). **(E)** RT-qPCR analysis of *DNAJC17* mRNA levels upon EMC4 ablation. **(F)** RT-qPCR analysis of EMC4 mRNA levels upon *DNAJC17* ablation. **(G)** RT-qPCR analysis of *DNAJC17* mRNA levels upon *DNAJC17* ablation. **(H)** Effect of the combined ablation of EMC4 and *DNAJC17* on pSyn^129^ levels. **(I)** Representative immunofluorescence images showing pSyn^129^ levels in cells with combined ablations of *EMC4* and *DNAJC17* (Green: HCS CellMask; Red: pSyn^129^/81A). Scale bar, 25 µm. **(J)** Representative immunofluorescence images showing pSyn^129^ levels in DNAJC17-activated cells (Green: HCS CellMask; Red: pSyn^129^/81A). Scale bar, 25 µm. Bar plots data are presented as mean ± SEM. Violin plots represent data distribution. Box plots display the median (centre line), the 75th percentile (top edge), and the 25th percentile (bottom edge). Statistical comparisons were performed using one-way ANOVA followed by Dunnett's post hoc test. *P < 0.05, **P < 0.01, ***P < 0.001.

**
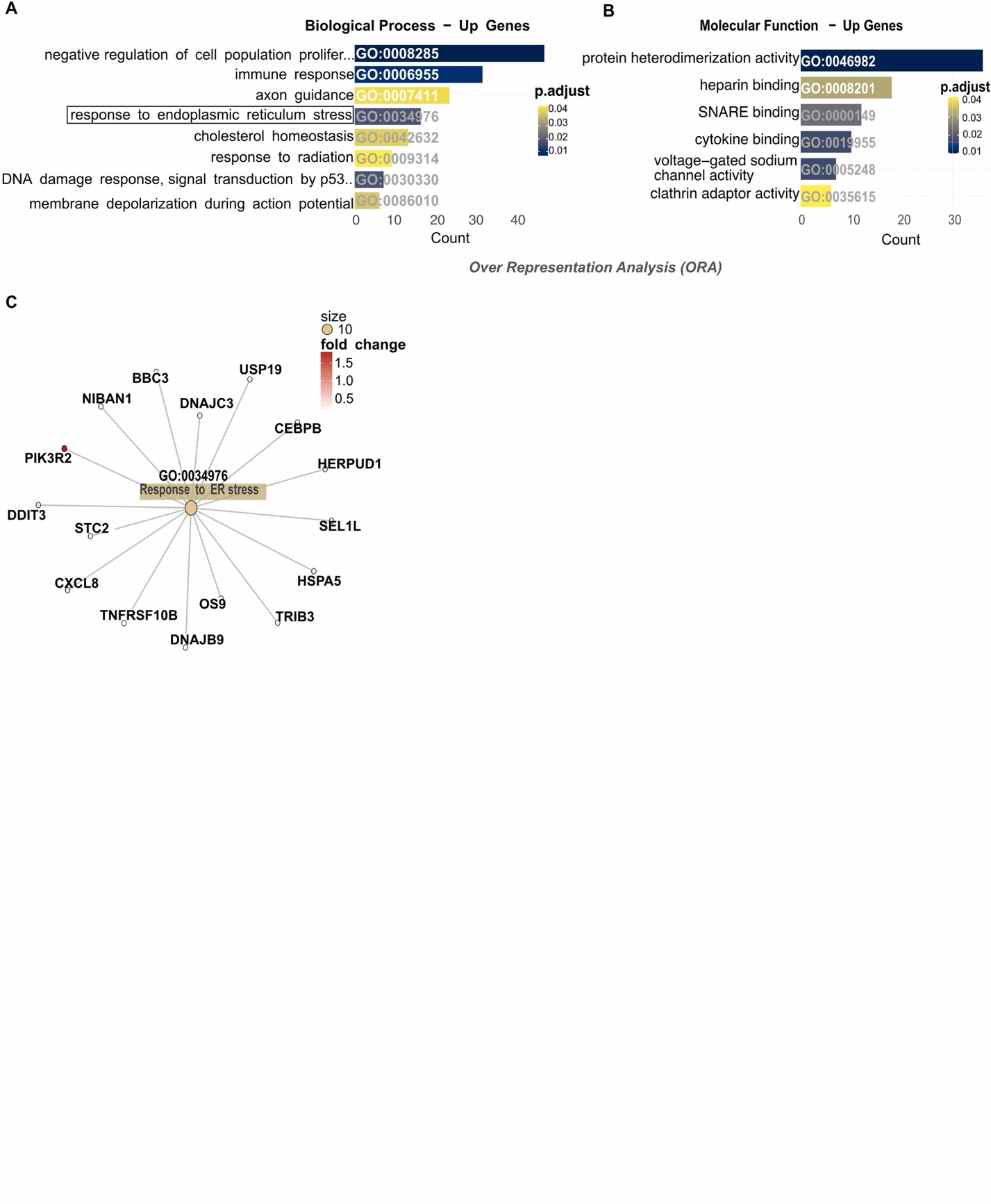
Figure S11: Pathway enrichment analysis in *EMC4* ablated** HEK^Syn^ **cells.** **(A)** Over-representation analysis (ORA) of biological processes for upregulated genes. Bar plot summarises enriched GO terms, highlighting ER stress-related pathways. Cut-off: candidate terms FDR ≤ 0.05. **(B)** ORA of molecular functions for upregulated genes. Cut-off: candidate terms FDR ≤ 0.05. **(C)** Network analysis of the response to endoplasmic reticulum stress (GO:0034976). Nodes represent genes, edges denote gene-term associations, and node colour intensity reflects fold change. Data are derived from RNA sequencing of *EMC4* ablation vs. non-targeting control cells.


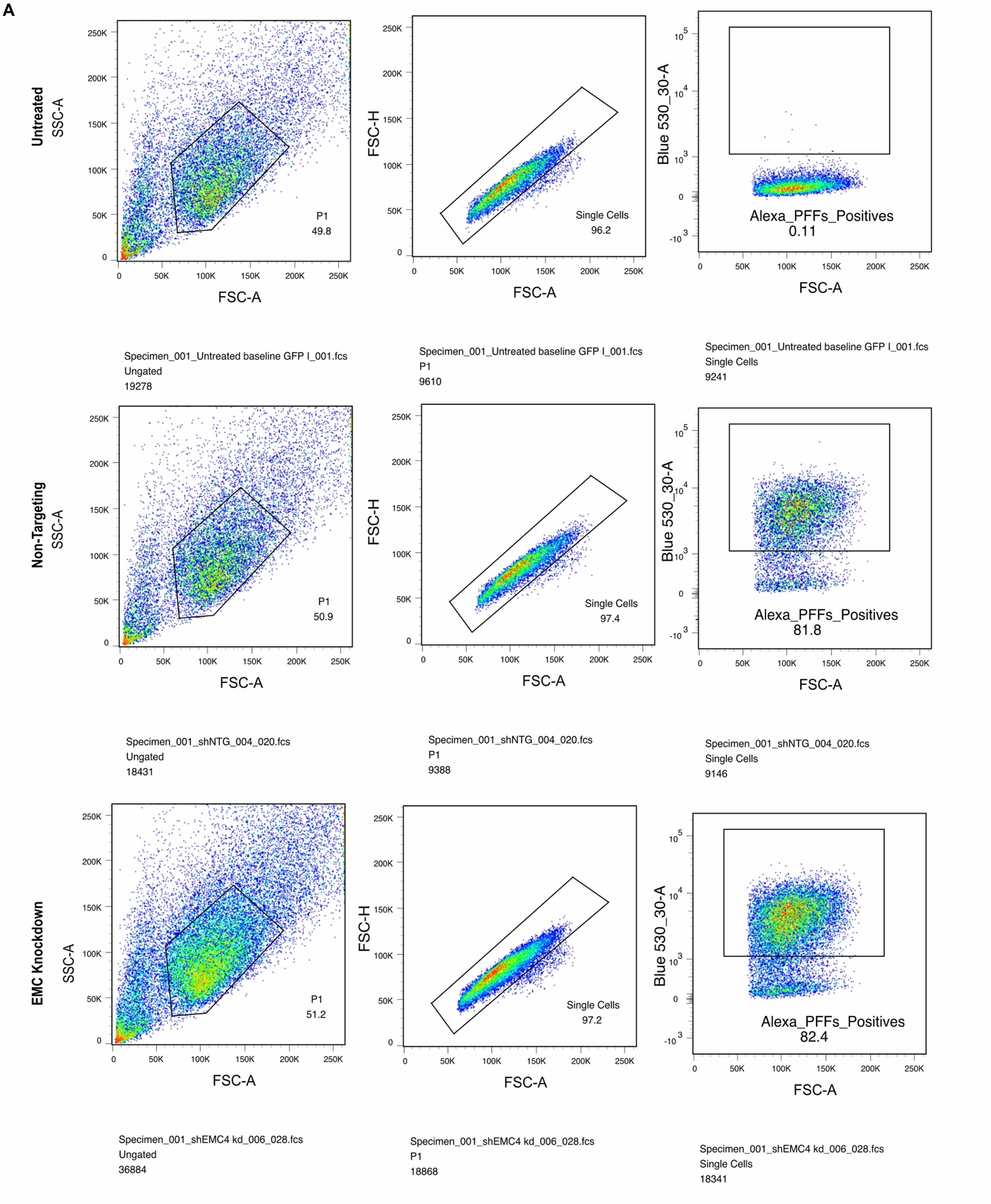


**Figure S12. Gating strategy for quantifying αSyn PFF uptake by flow cytometry.**

**(A)** Representative flow cytometry plots of iPSC-derived neurons illustrating the sequential gating strategy used to quantify Alexa Fluor 488-labelled αSyn PFF-positive cells across experimental conditions.

**
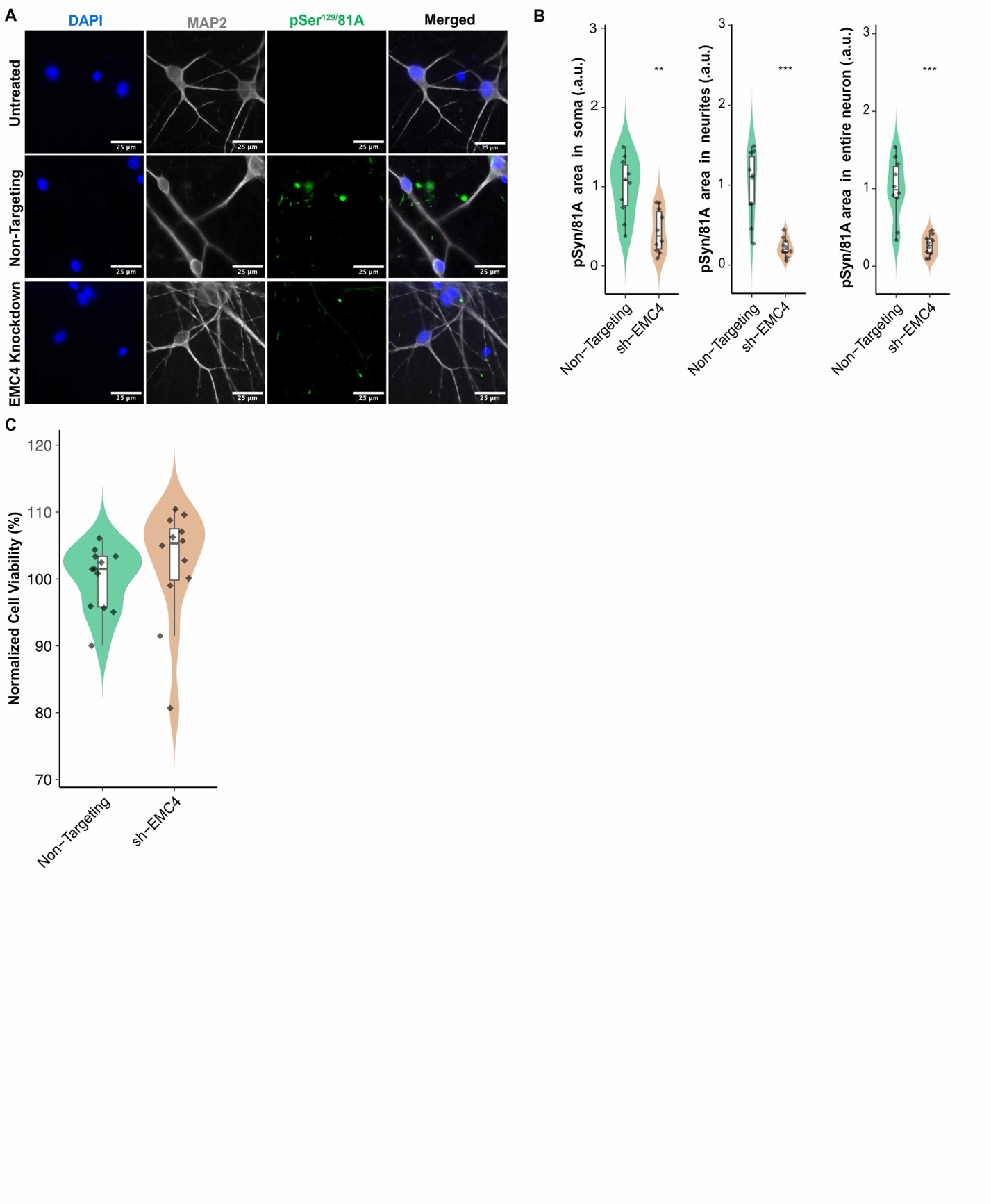
**

**Figure S13. Assessment of αSynuclein aggregate levels and toxicity in iPSC-derived cortical neurons upon EMC4 knockdown. (A)** Neurons subjected to EMC4 shRNA or to control conditions. DAPI (blue), MAP2-labeled neurons (grey), and 81A stained pSyn^129^ aggregates (green). **(B)** Quantification of pSyn^129^ spot area: (left) in soma, (middle) neurites and (right) whole neurons, normalized to respective MAP2 area. Scale bar, 25 µm. (**C)** CellTiter-Glo assay measuring cell viability in i^3^ cortical neurons following EMC4 knockdown relative to the non-targeting control. Violin plots represent data distribution. Inner box plots display the median (centre line), the 75th percentile (top edge), and the 25th percentile (bottom edge). Statistical comparisons were performed using Welch’s t-test (unequal variance t-test) with significance levels denoted as follows: *P < 0.05, **P < 0.01, ***P < 0.001.
