# Supplementary material for: Large‐scale bidirectional arrayed genetic screens identify OXR1 and EMC4 as modifiers of αSynuclein aggregation
Source: FEBS Open Bio. 2026 Mar 30;16(8):1563–92. doi: 10.1002/2211-5463.70233 (PMC13398675; doi:10.1002/2211-5463.70233)
Supplement: Supplementary file 9 — Data S1. Uncut western blot. [file FEB4-16-1563-s008.docx]

**Western Blot Uncropped Data**

**Figure 1**

**Figure 1a**

i dCas9 and Cas9

**
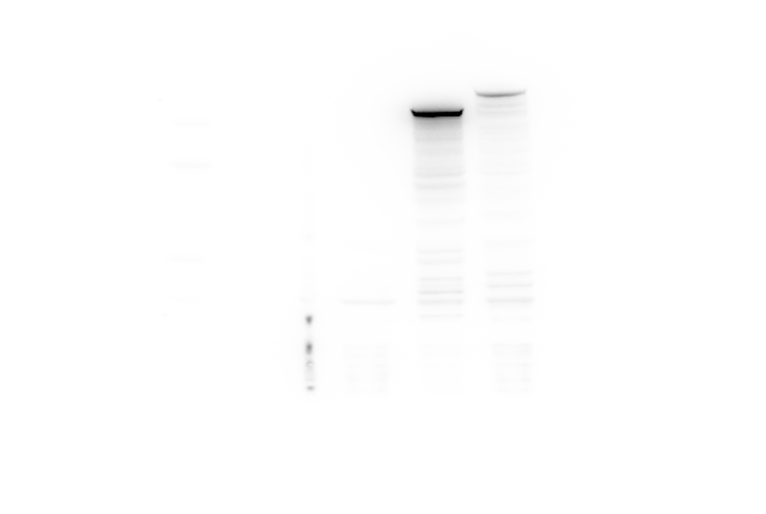
**ii B-actin

**
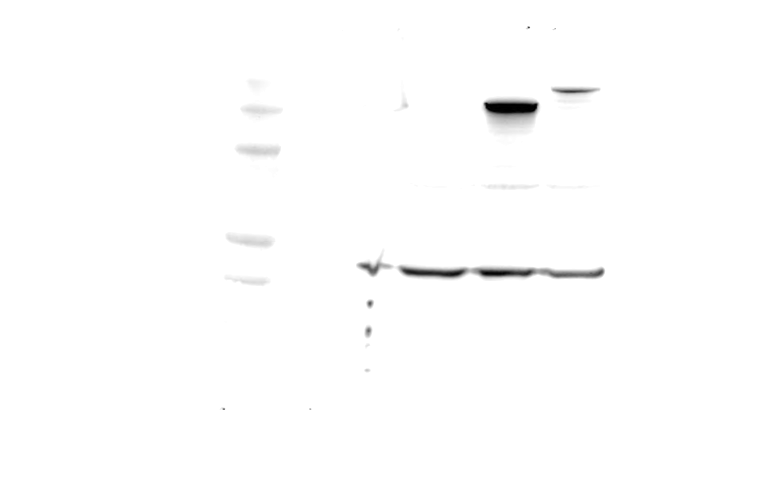
**

iii α-Synuclein

**
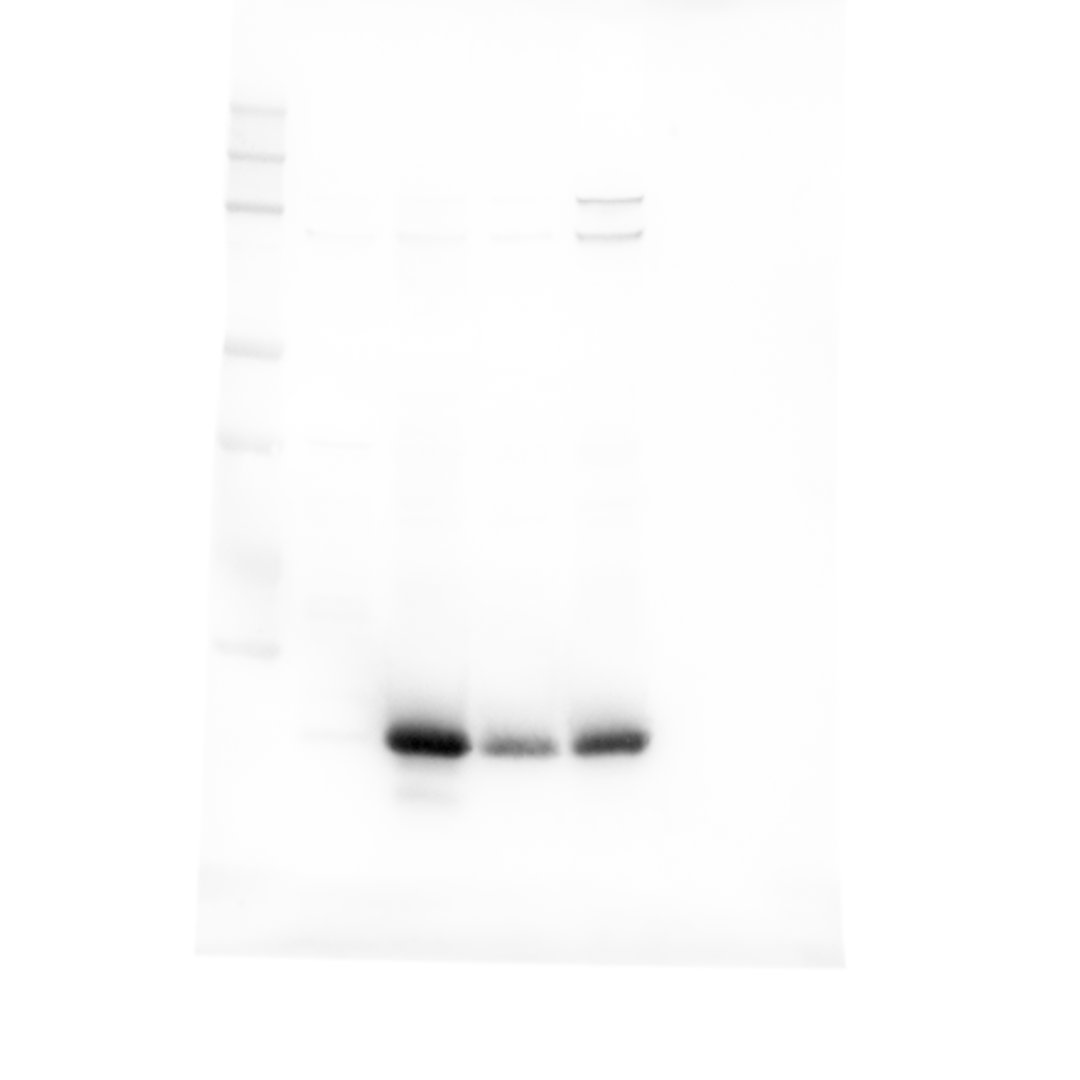
**

iv B-Actin

**
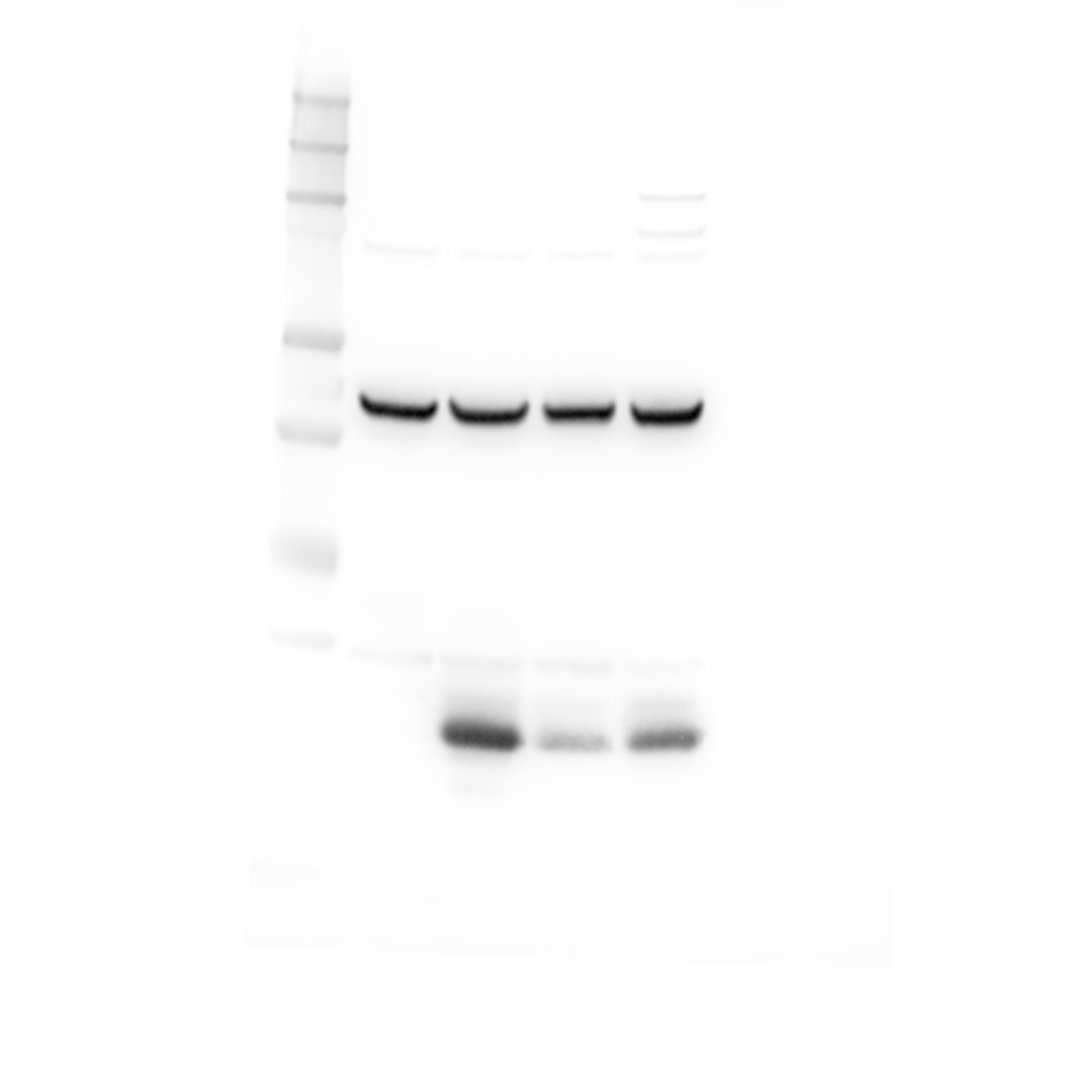
**

**Figure 1c**

i KDM4A

**
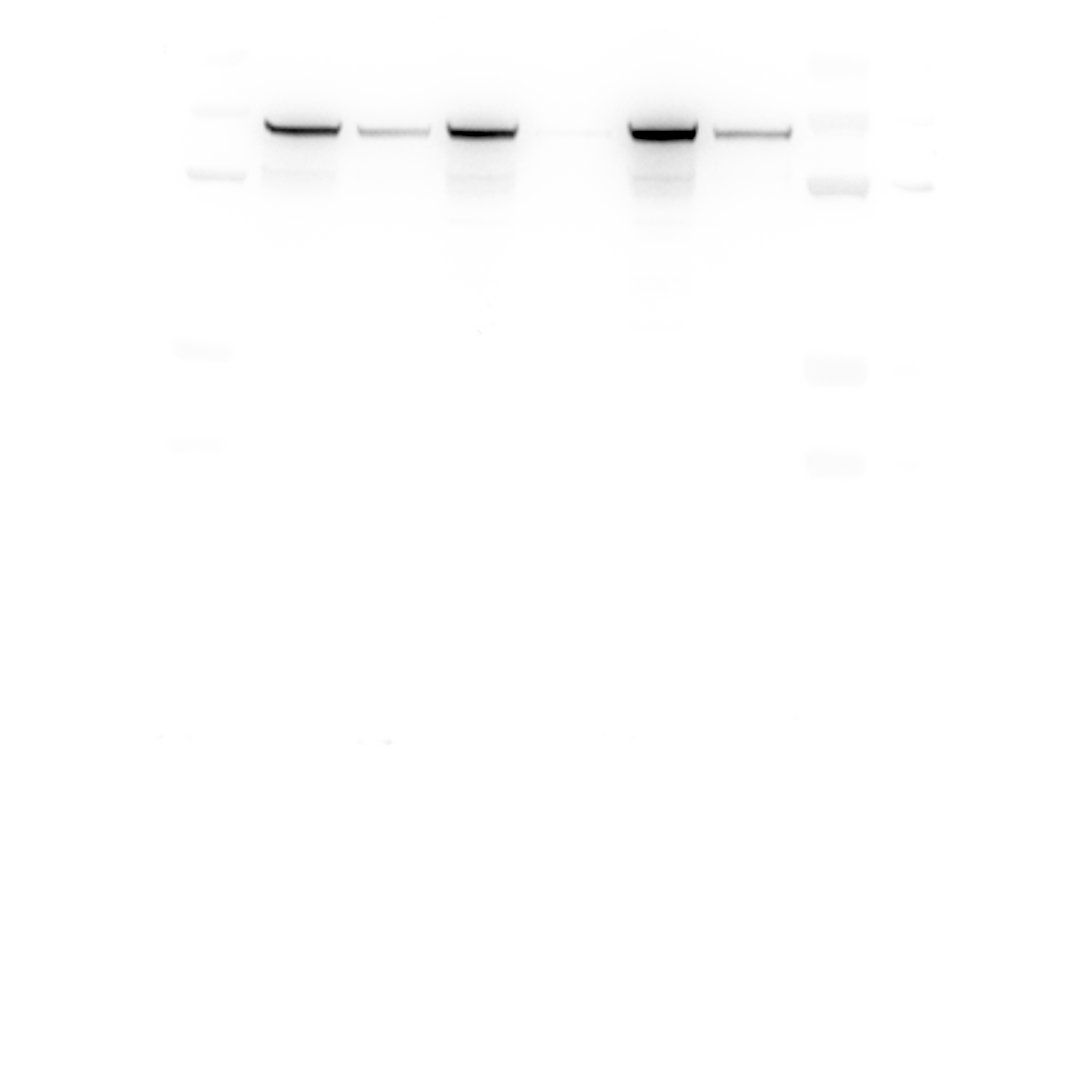
**

ii B-Actin

**
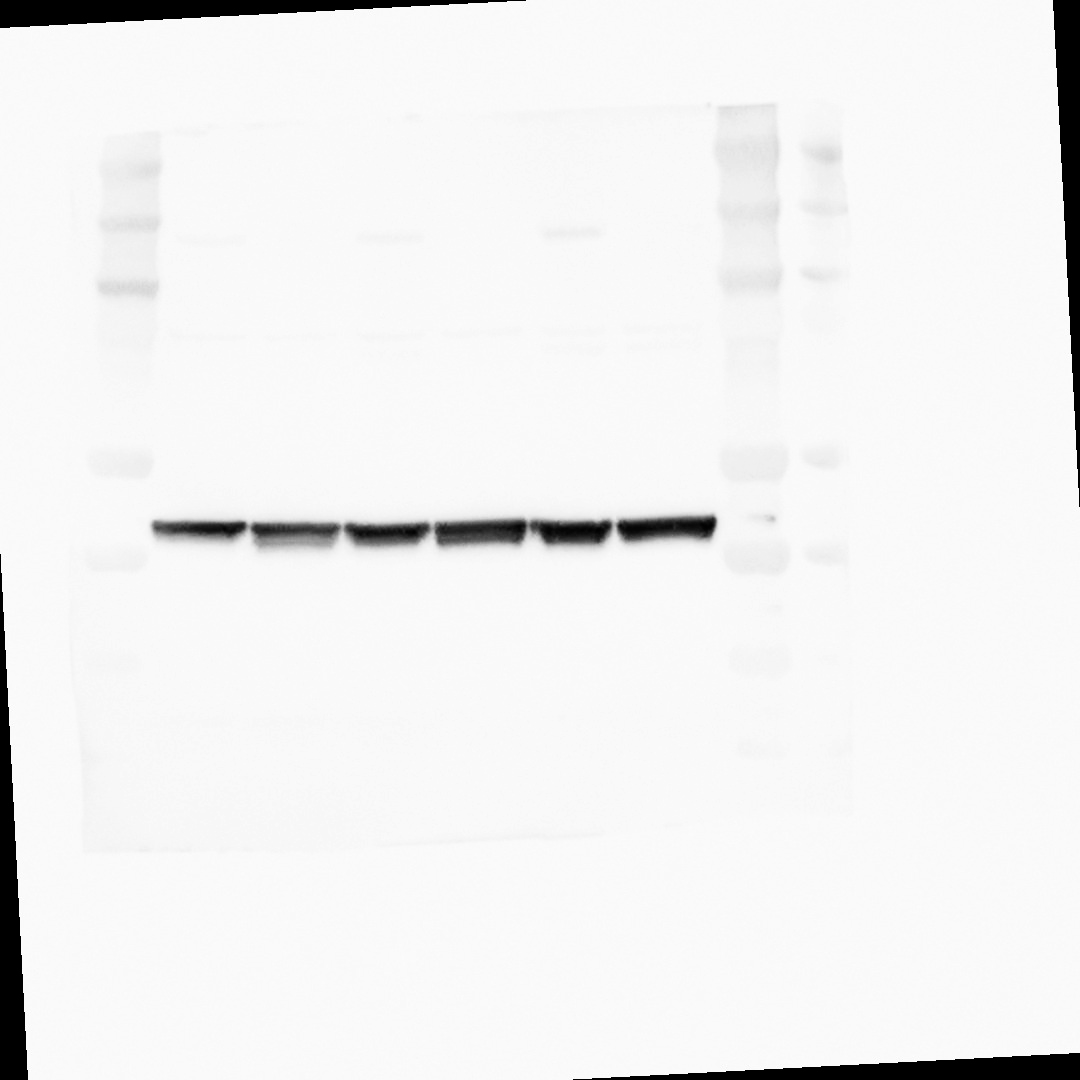
**

**Figure 3**

**Figure 3c**

i EMC4


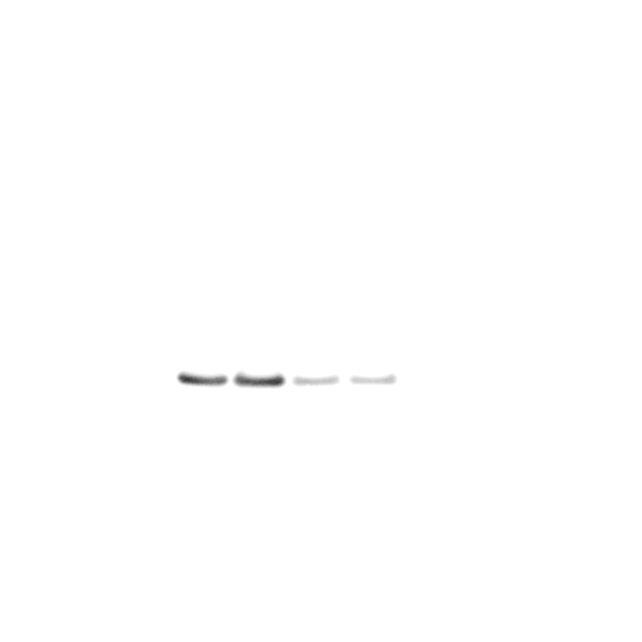


ii B-Actin

**
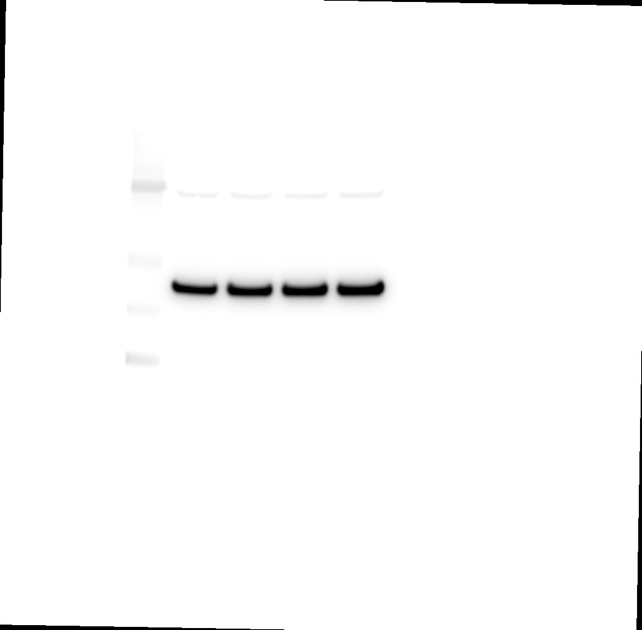
**

iii α-Synuclein

**
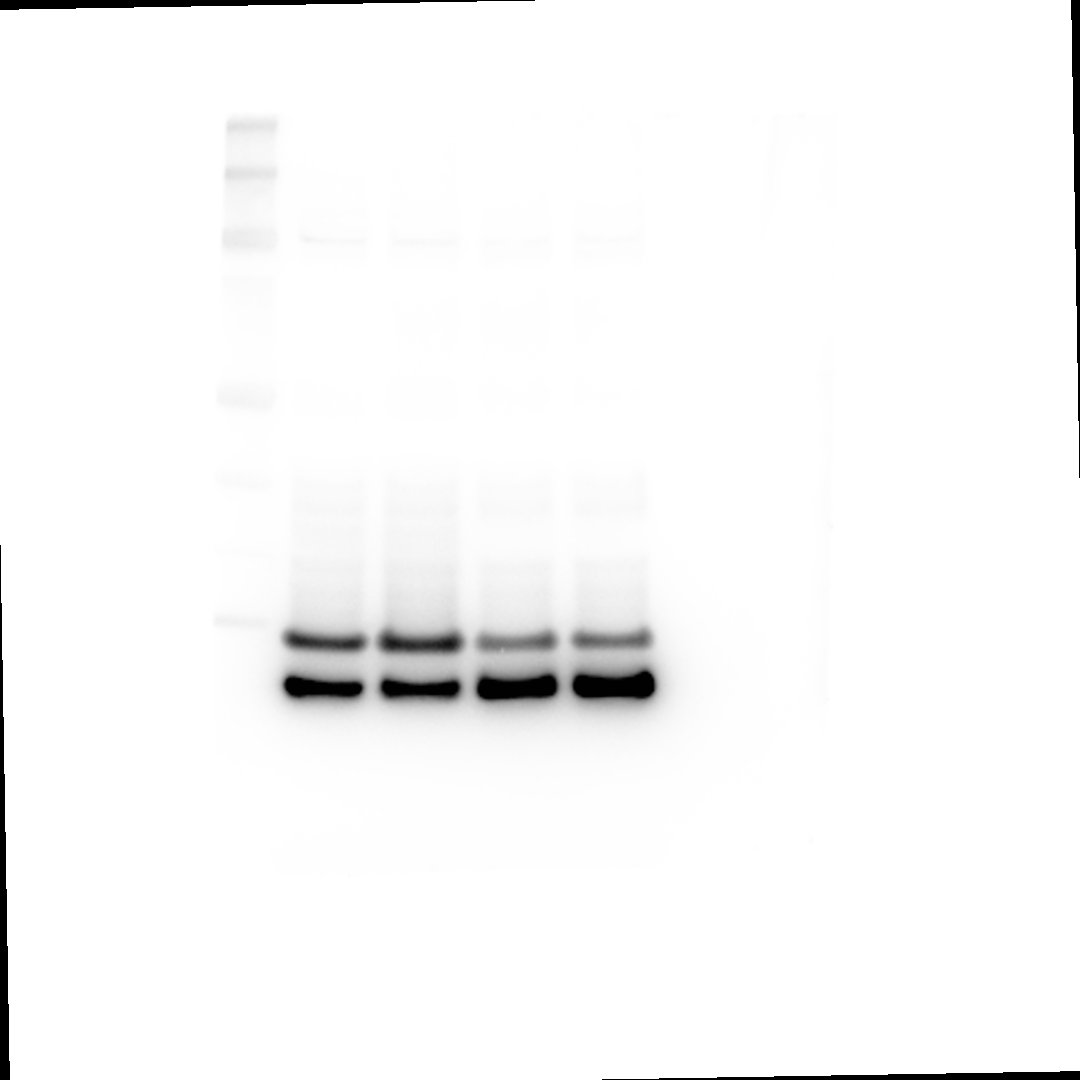
**

**Figure 5**

**Figure 5c**

i OXR1

**
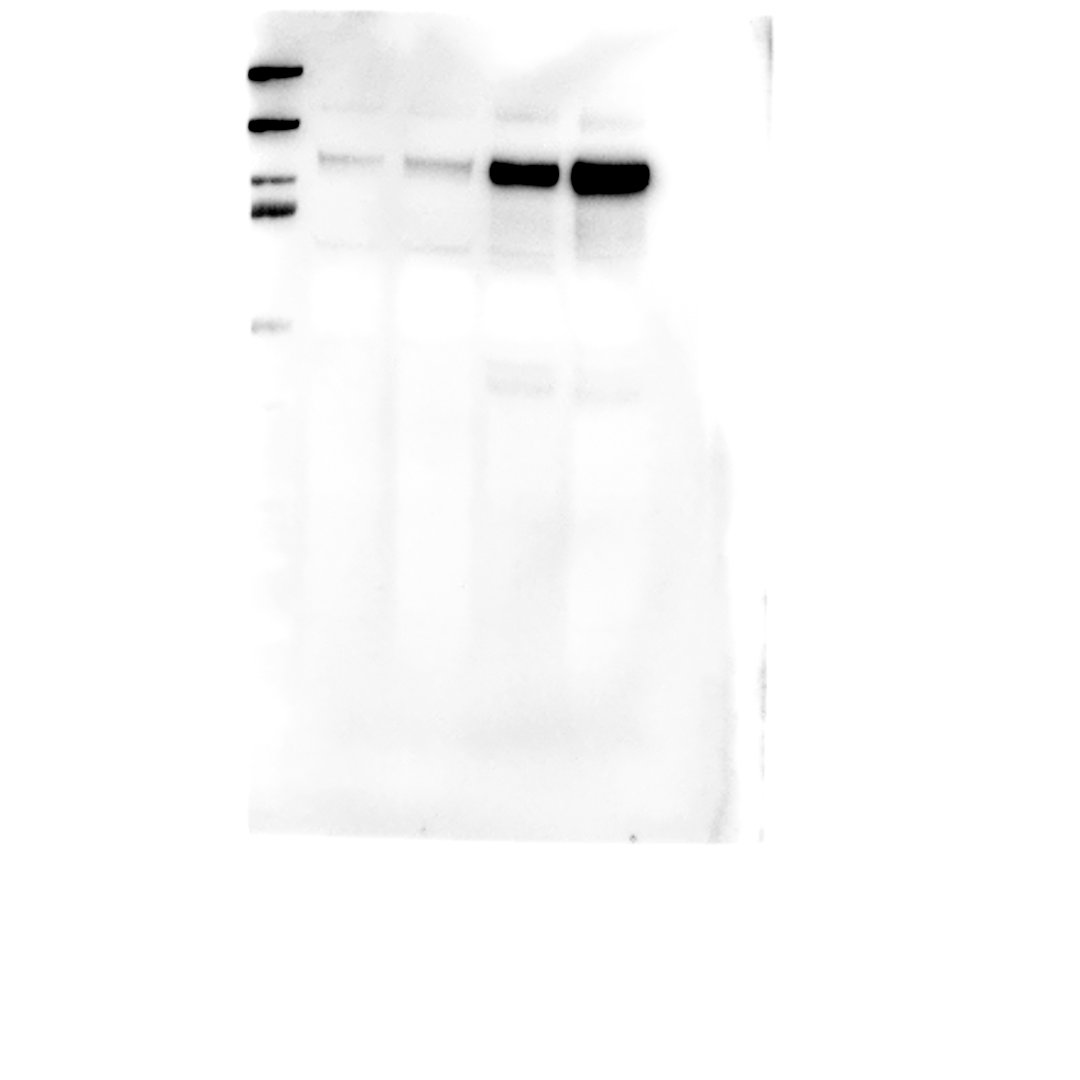
**

ii B-Actin

**
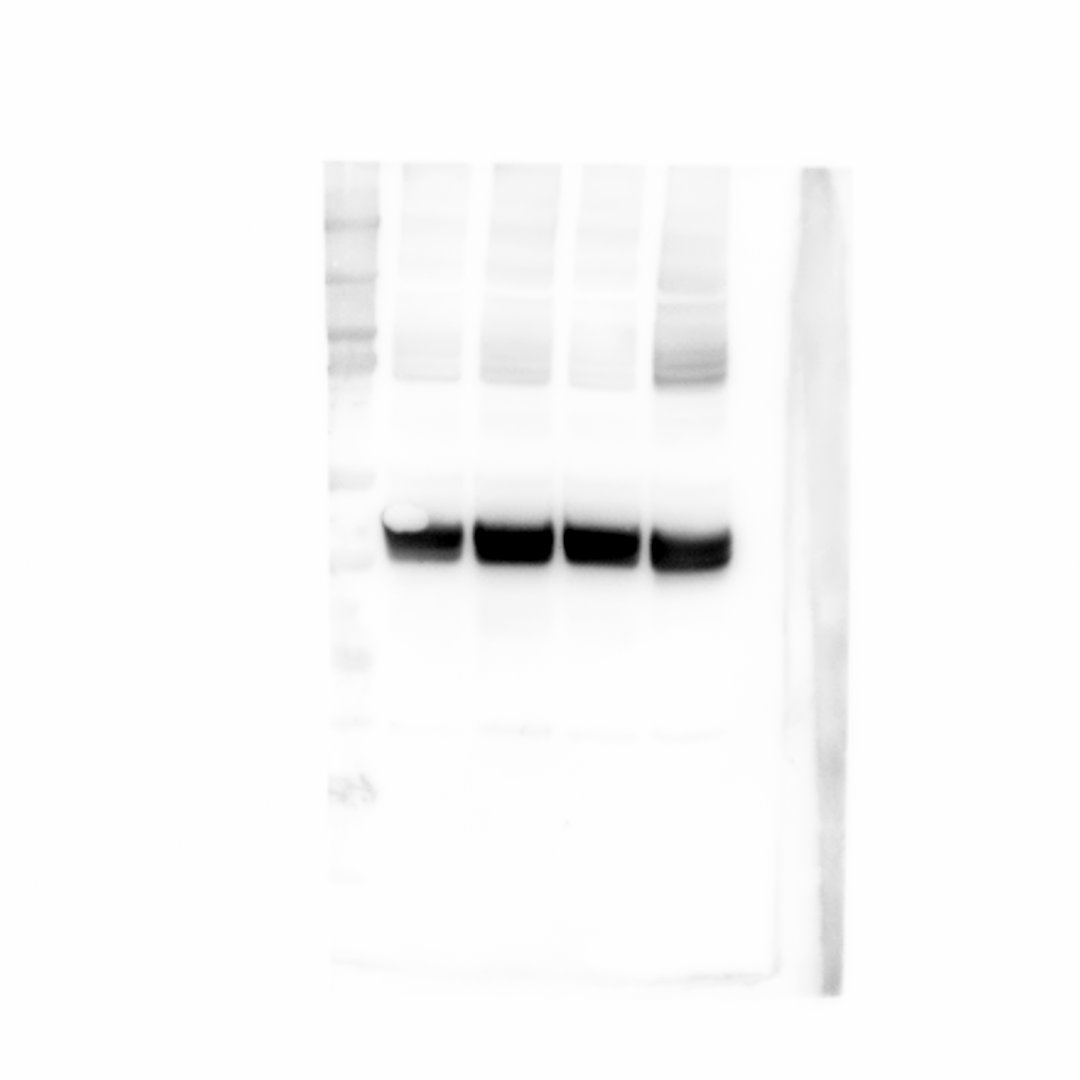
**

**Figure 6**

**Figure 6c**

i α-Synuclein

**
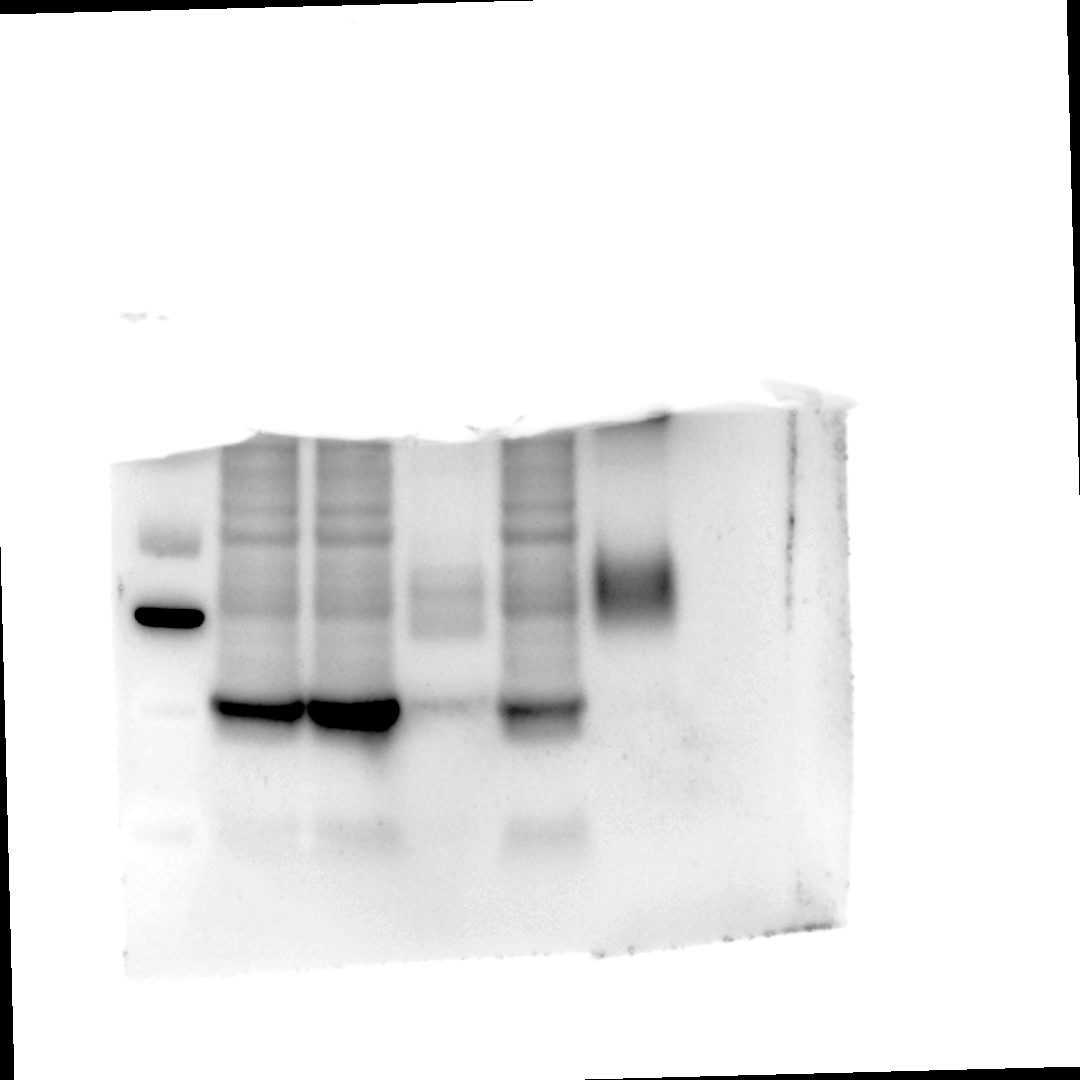
**

ii EMC4

**
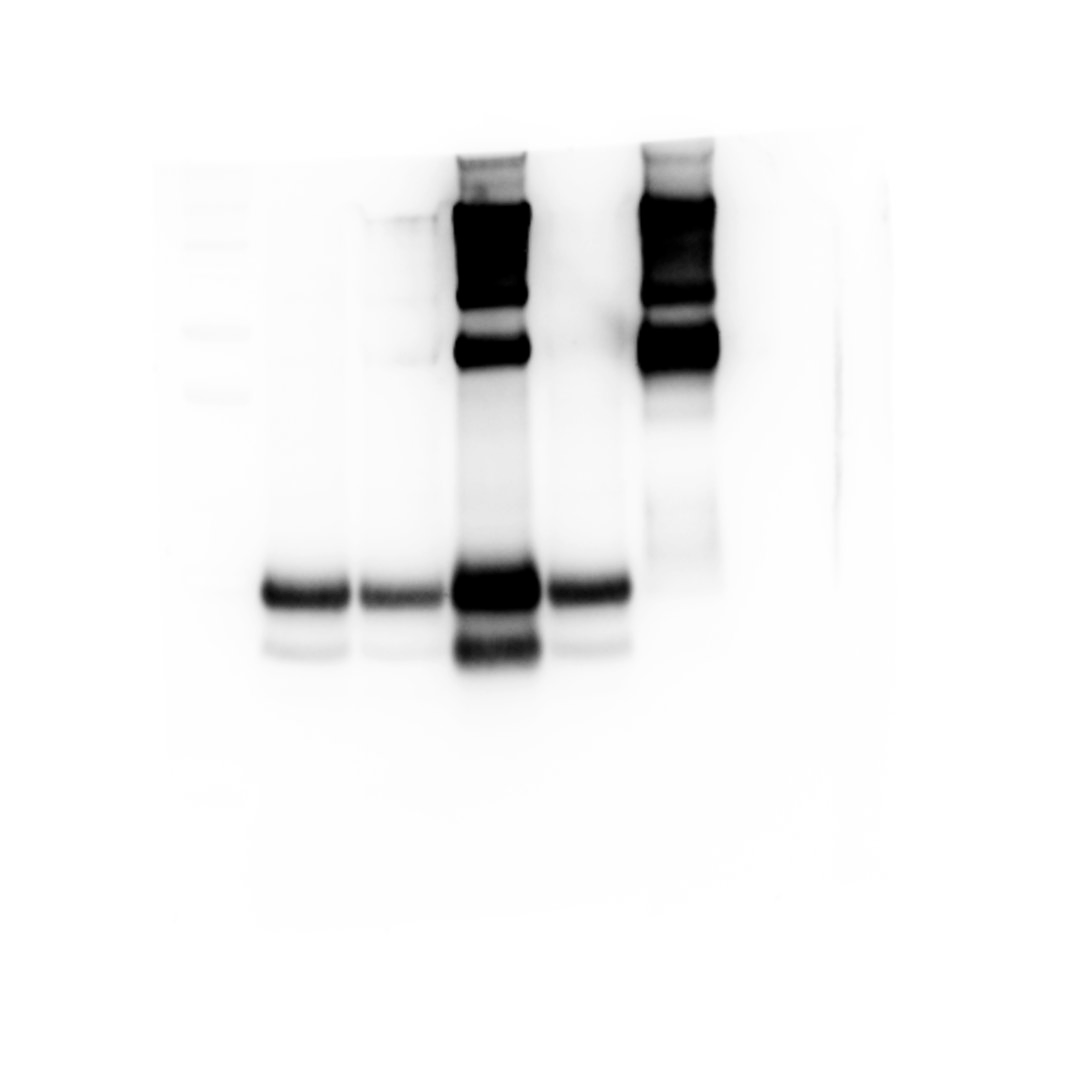
**

iii EMC4

**
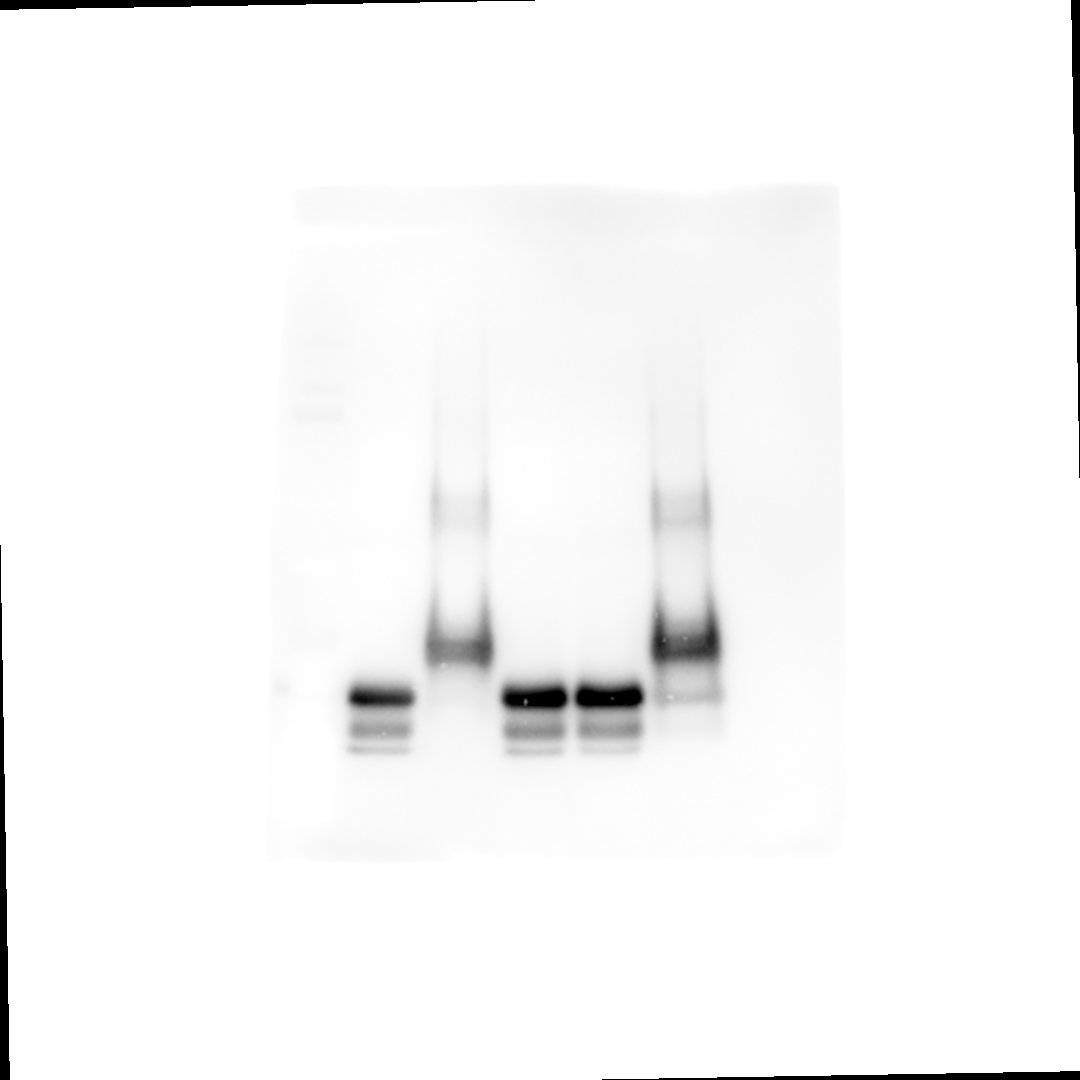
**

iv α-Synuclein

**
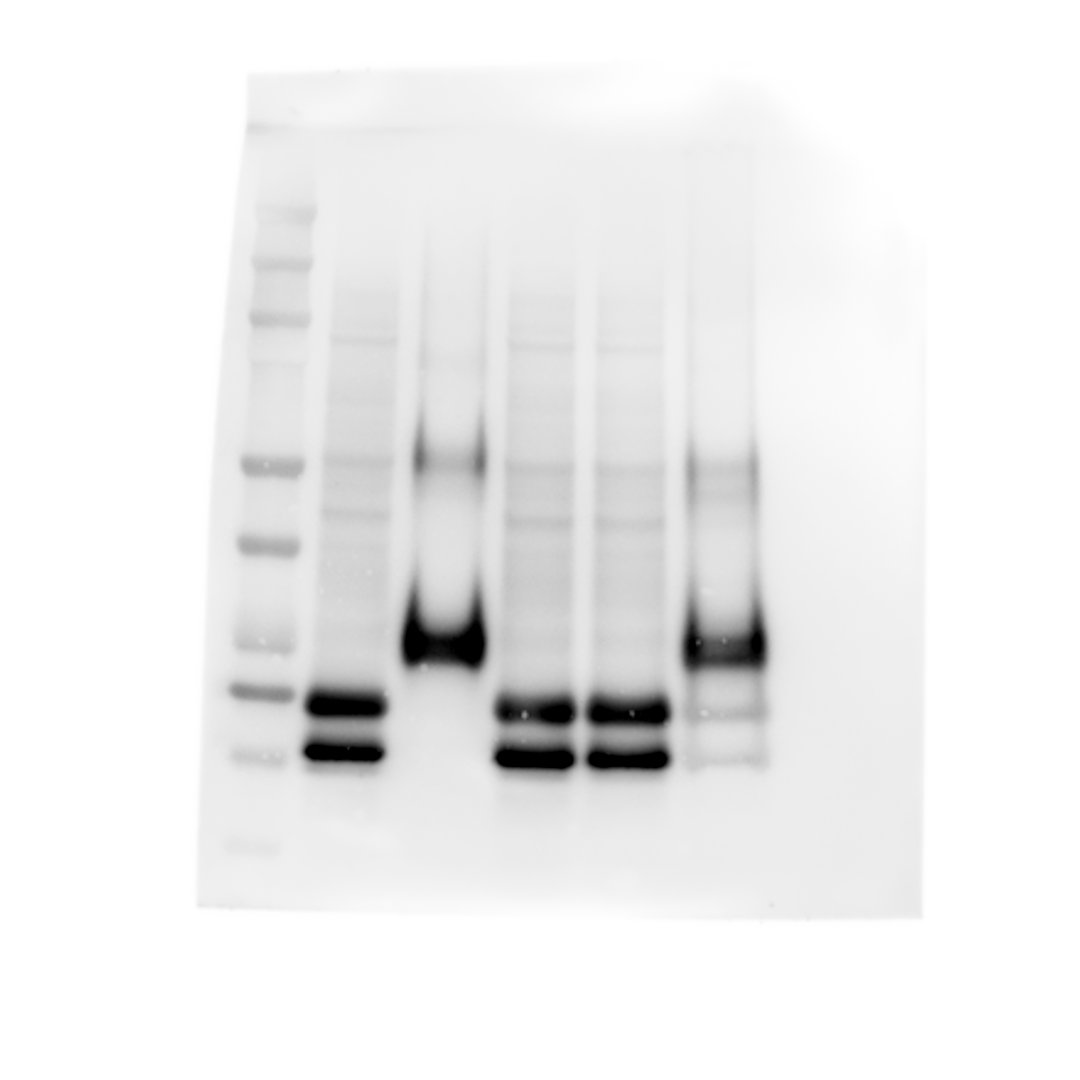
**

**Figure 6 e**

i LAMP1

**
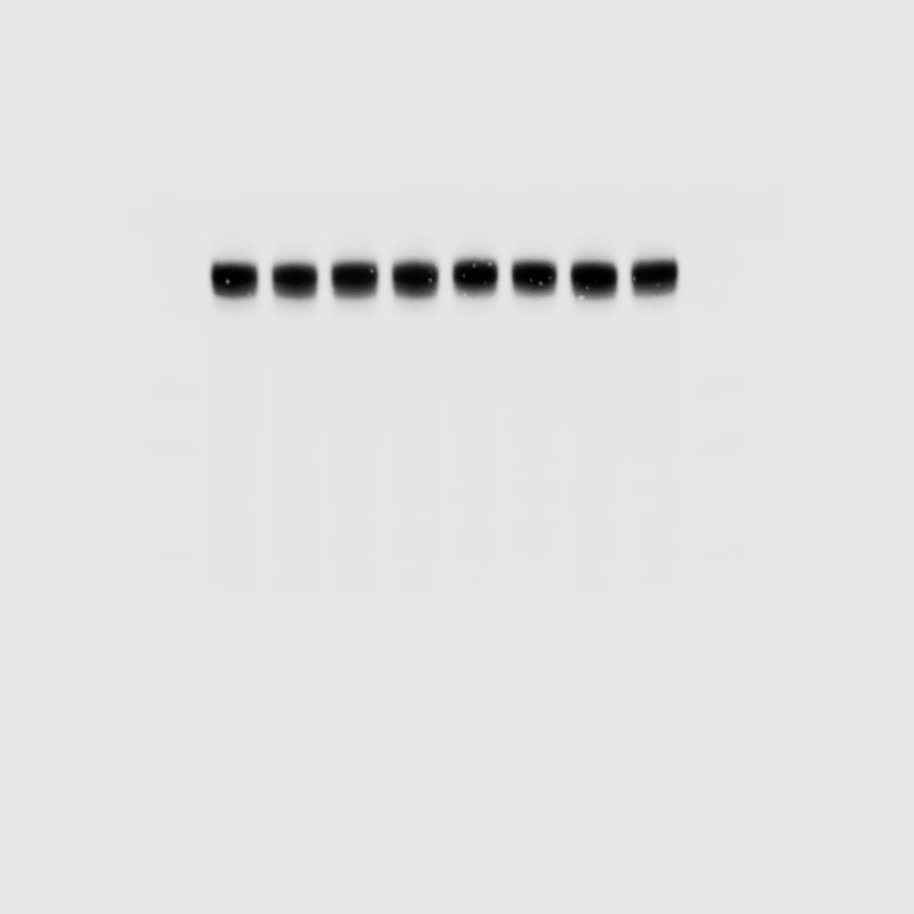
**

ii LC3B

**
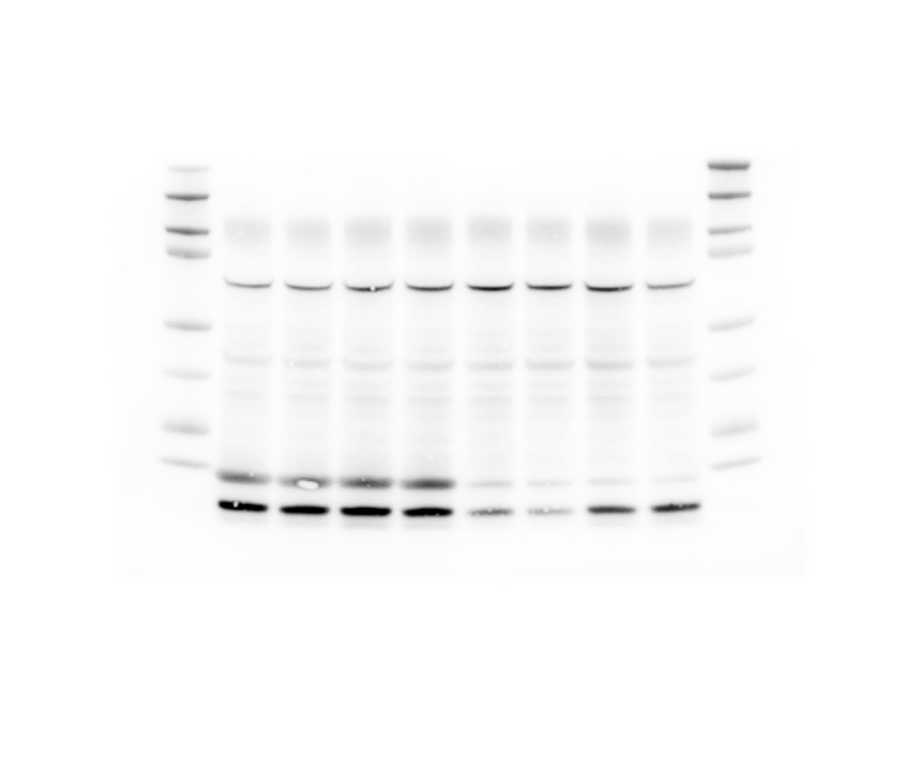
**

iii p62

**
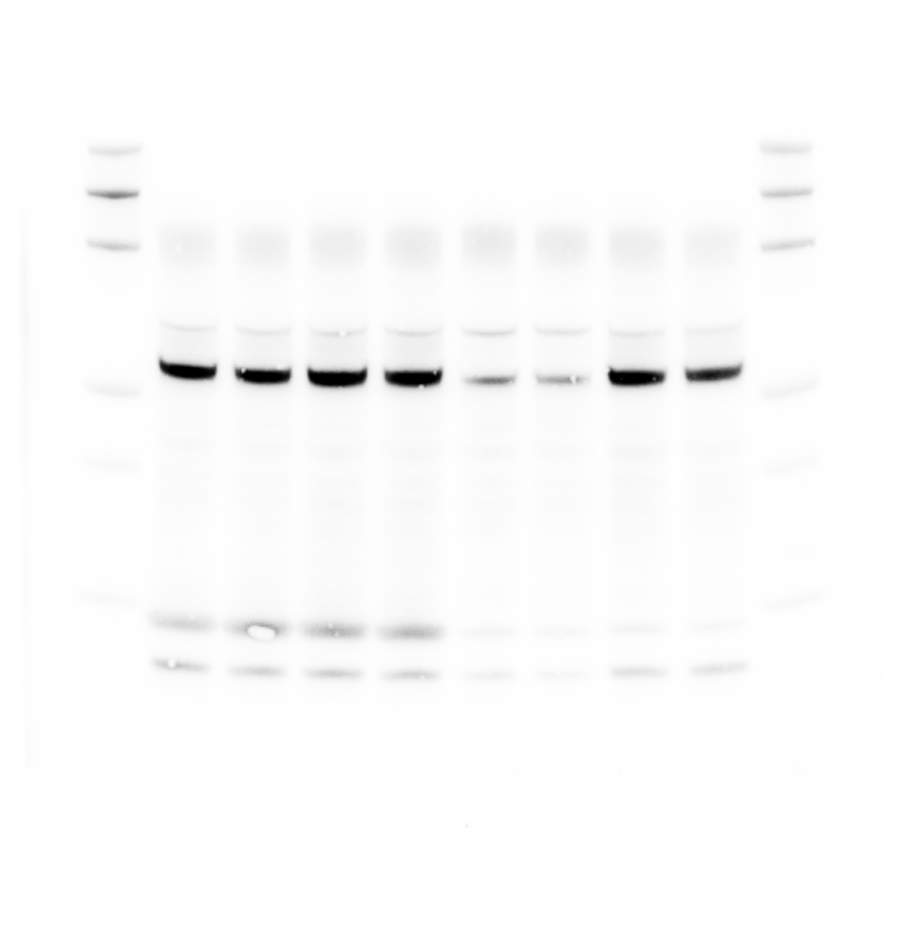
**

iv EMC4

**
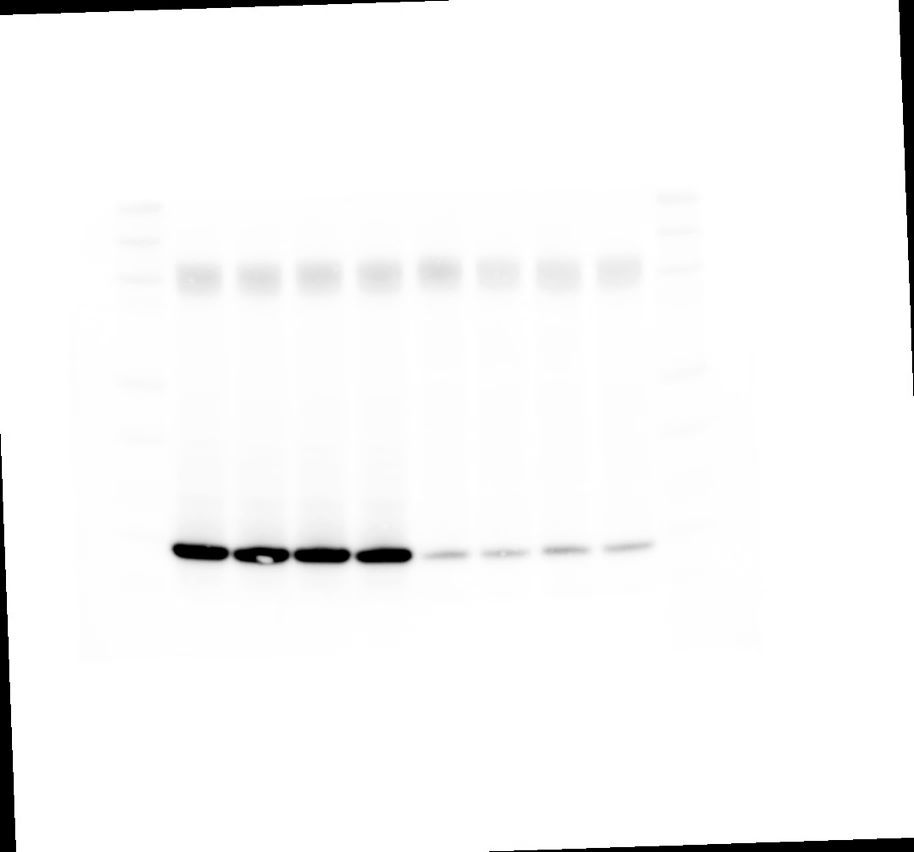
**

v B-Actin **
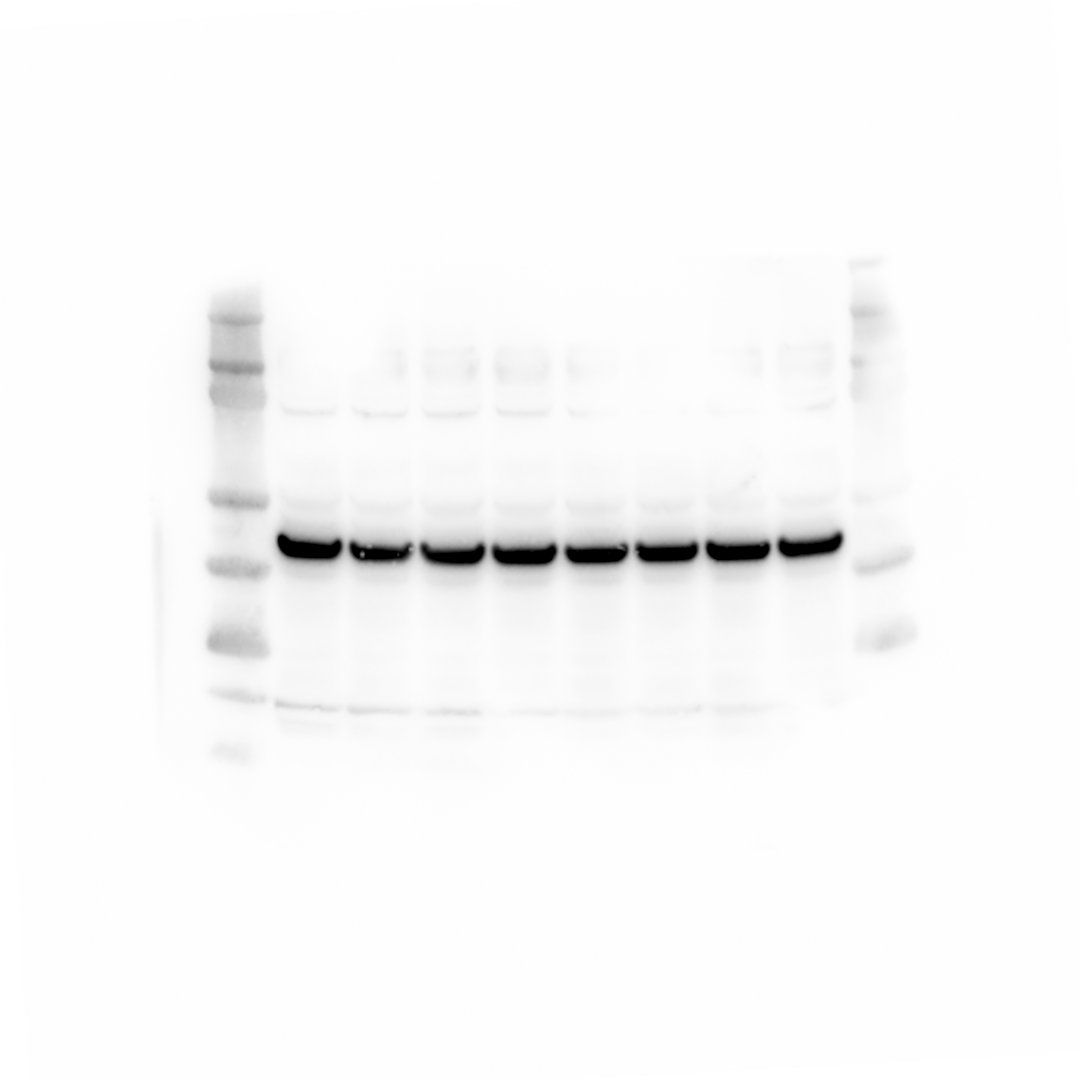
**

**Figure 7**

i EMC4

**
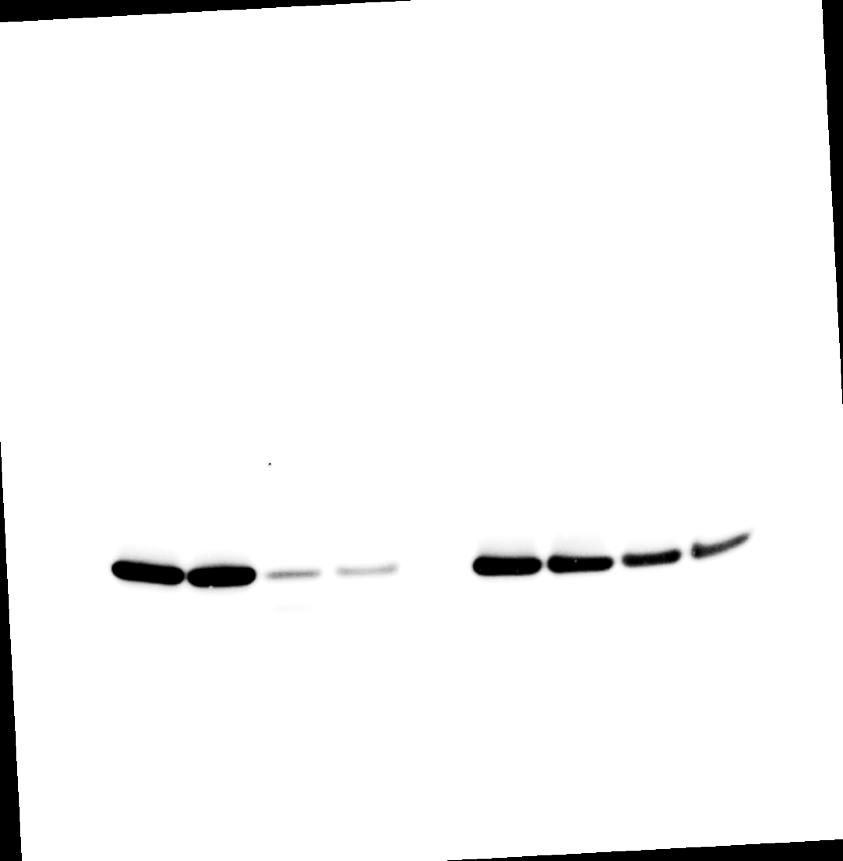
**

ii LC3B

**
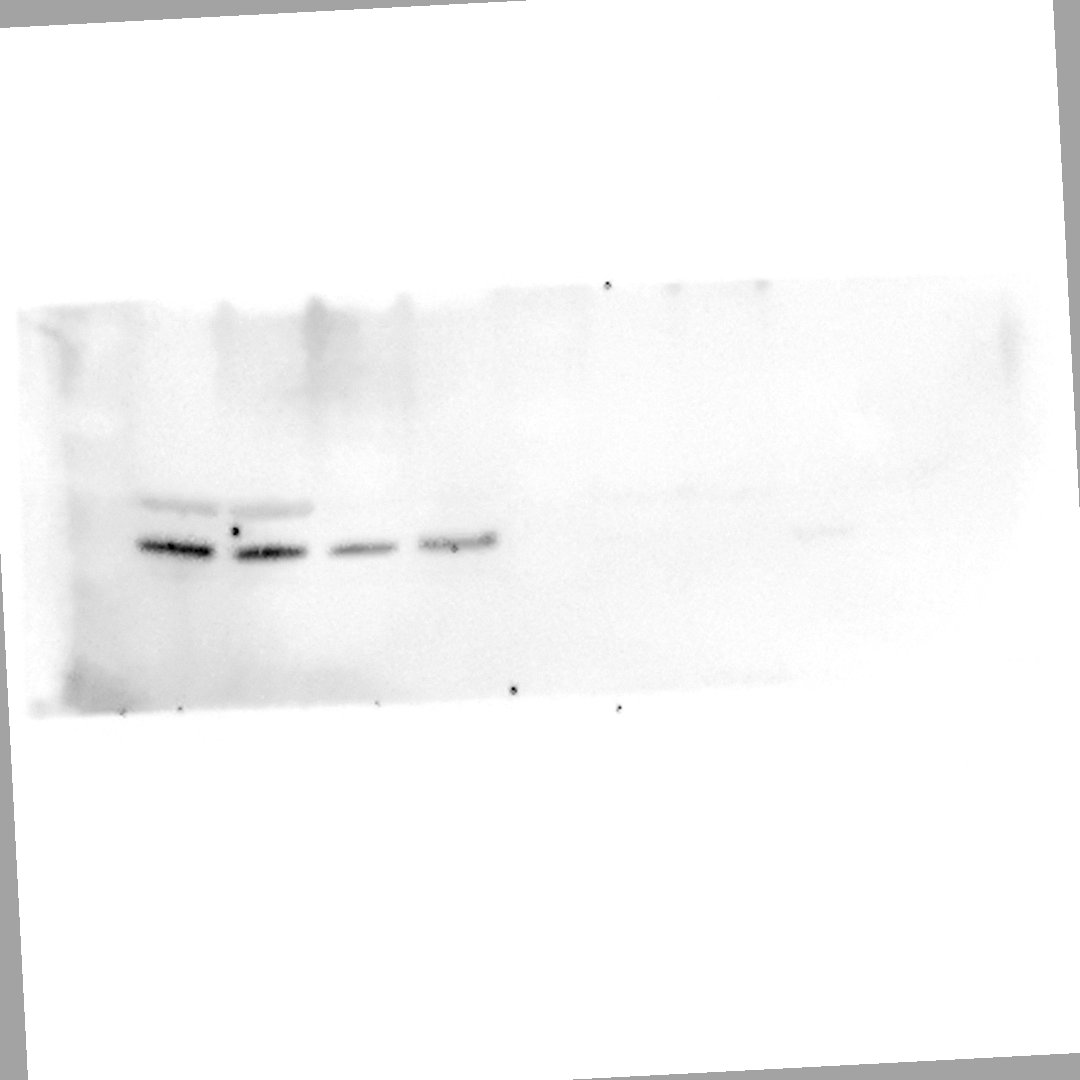
**

iii LAMP1

**
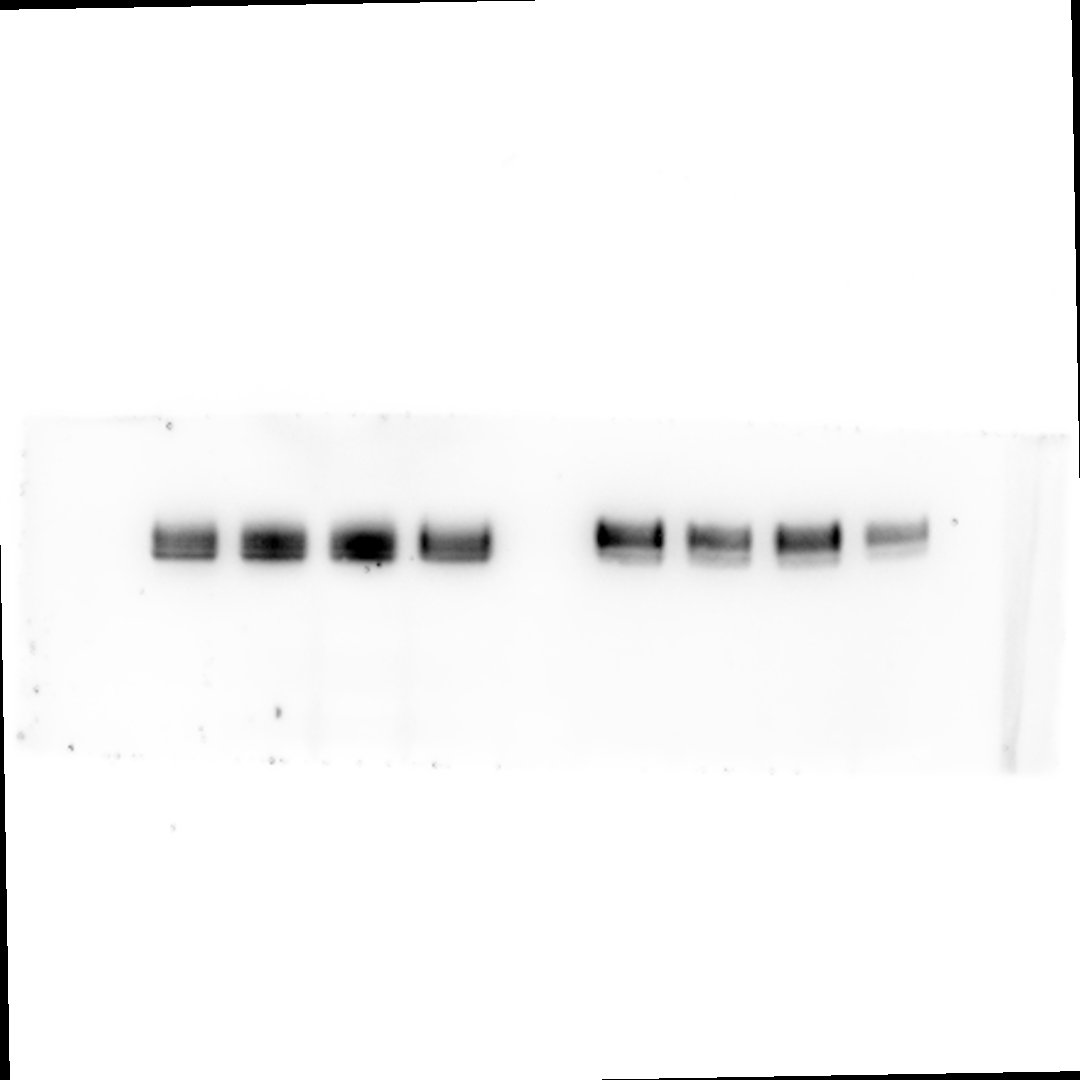
**

iv B-Actin

**
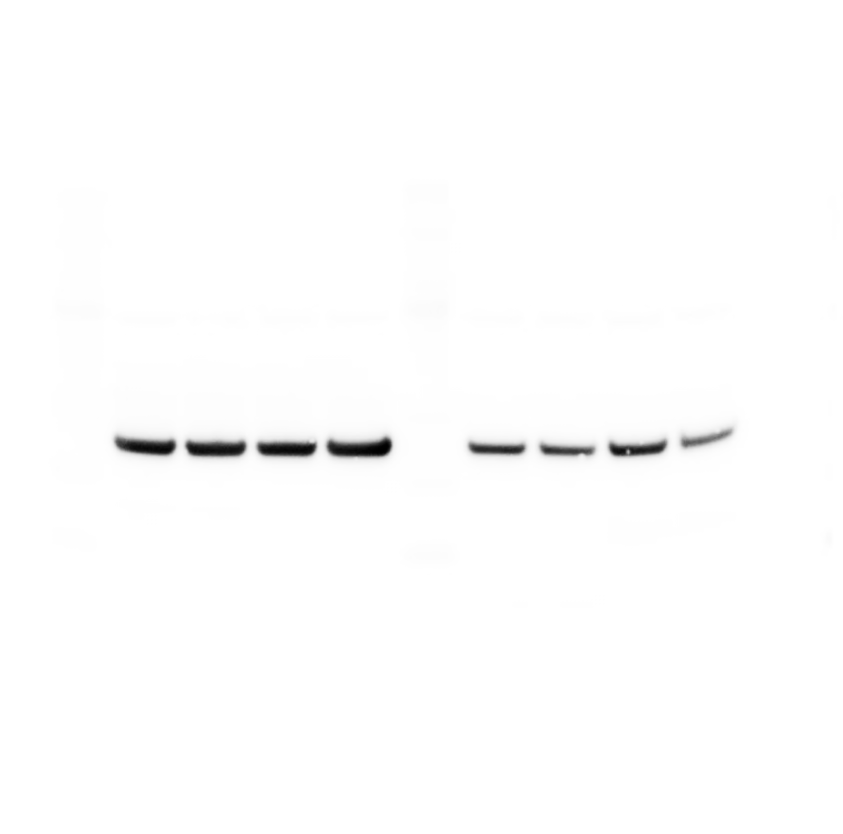
**

**Supplementary Figure 1**

**Supp. 1 c**

i) RAB13

**
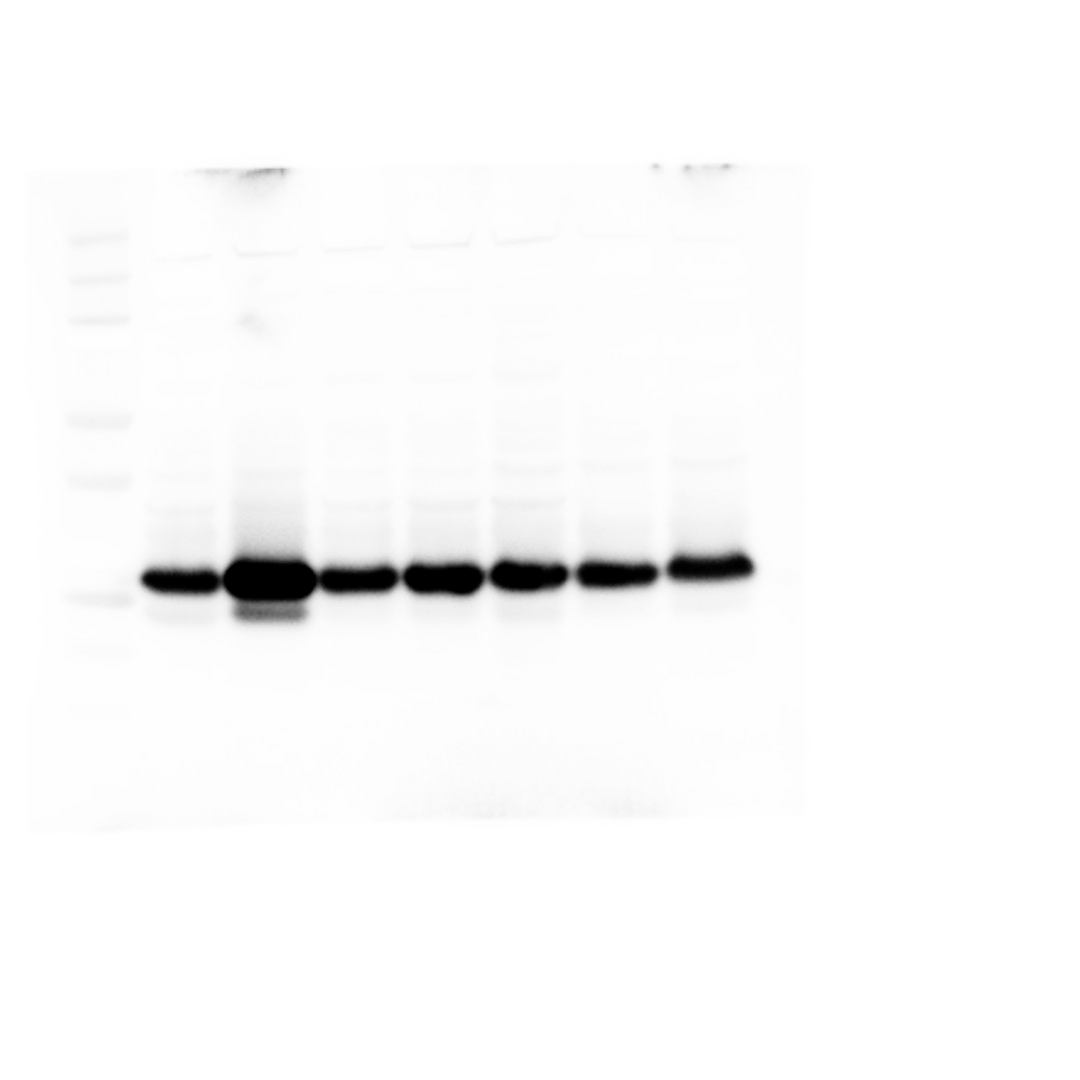
**

ii) B-Actin

**
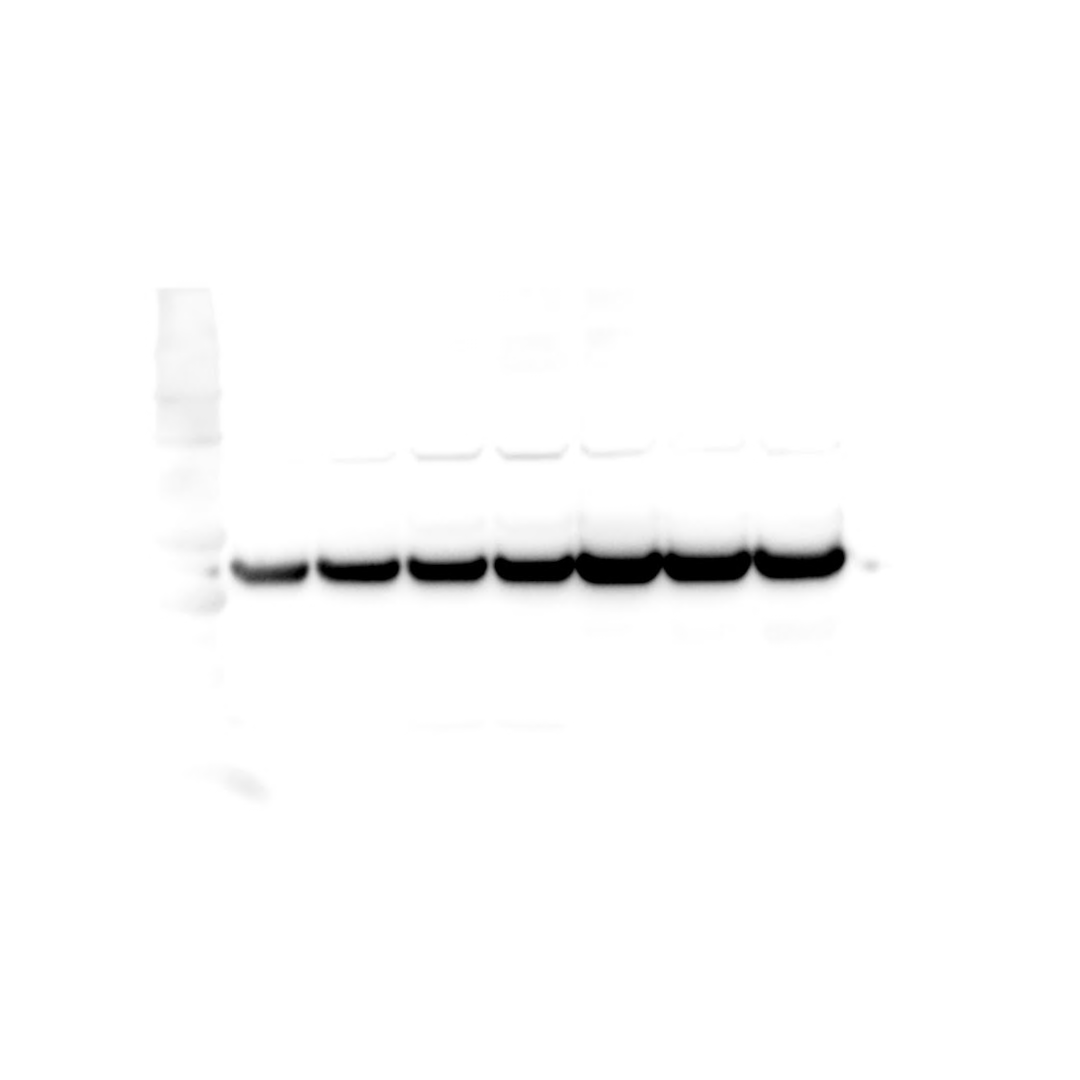
**

**Supp. 1e)**

i) TFAP2C

**
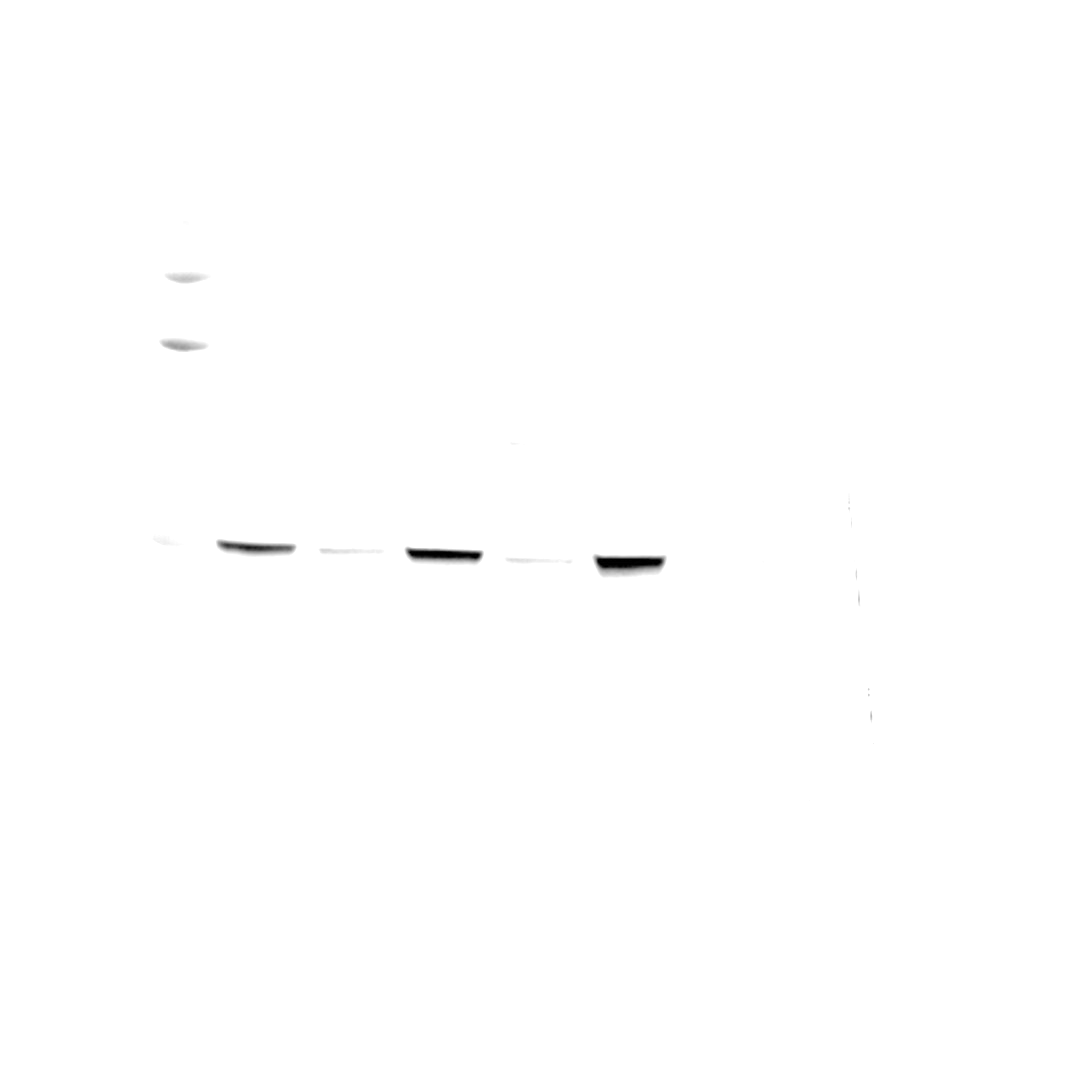
**

ii) B-Actin

**
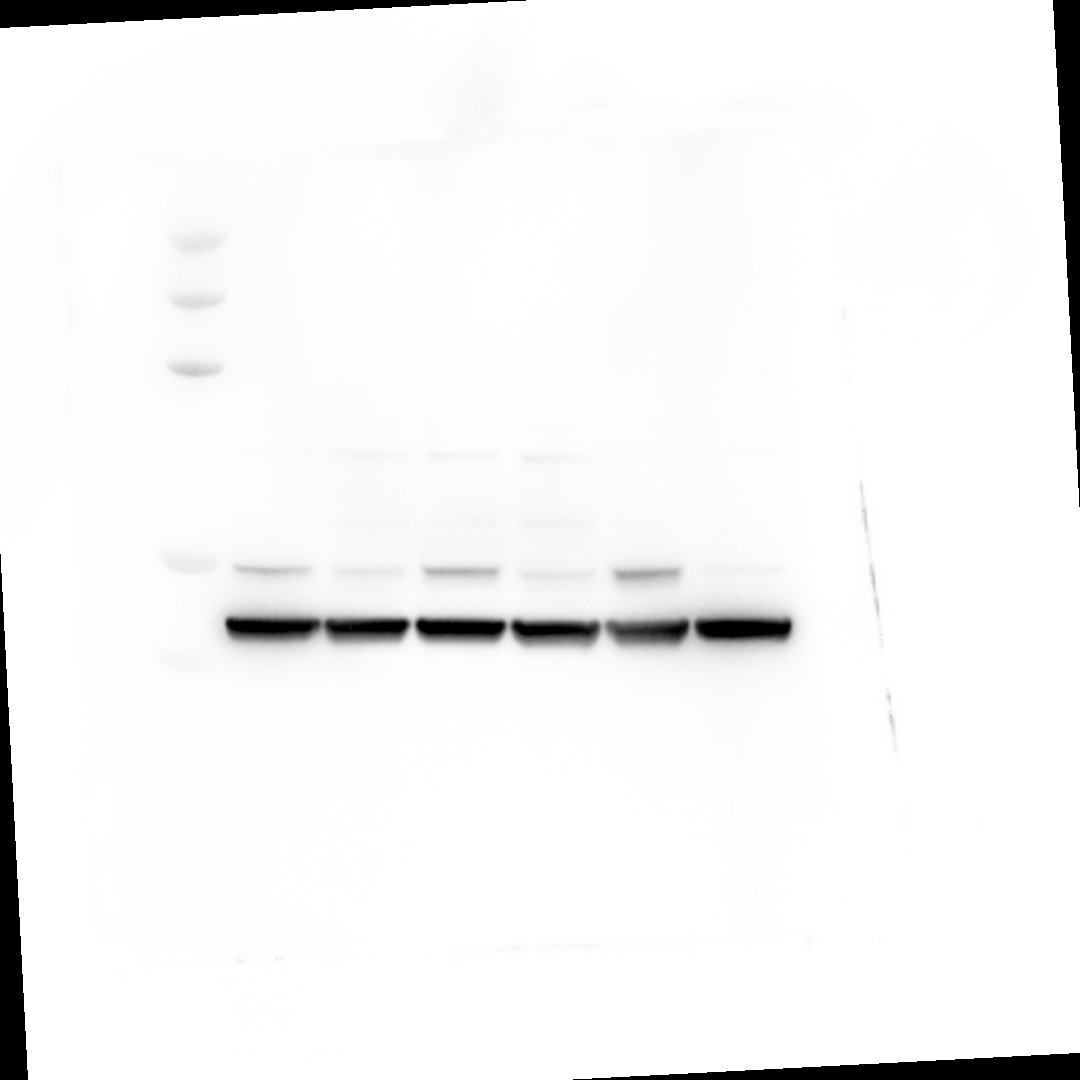
**

**Supp. 1f)** i) TFEB

**
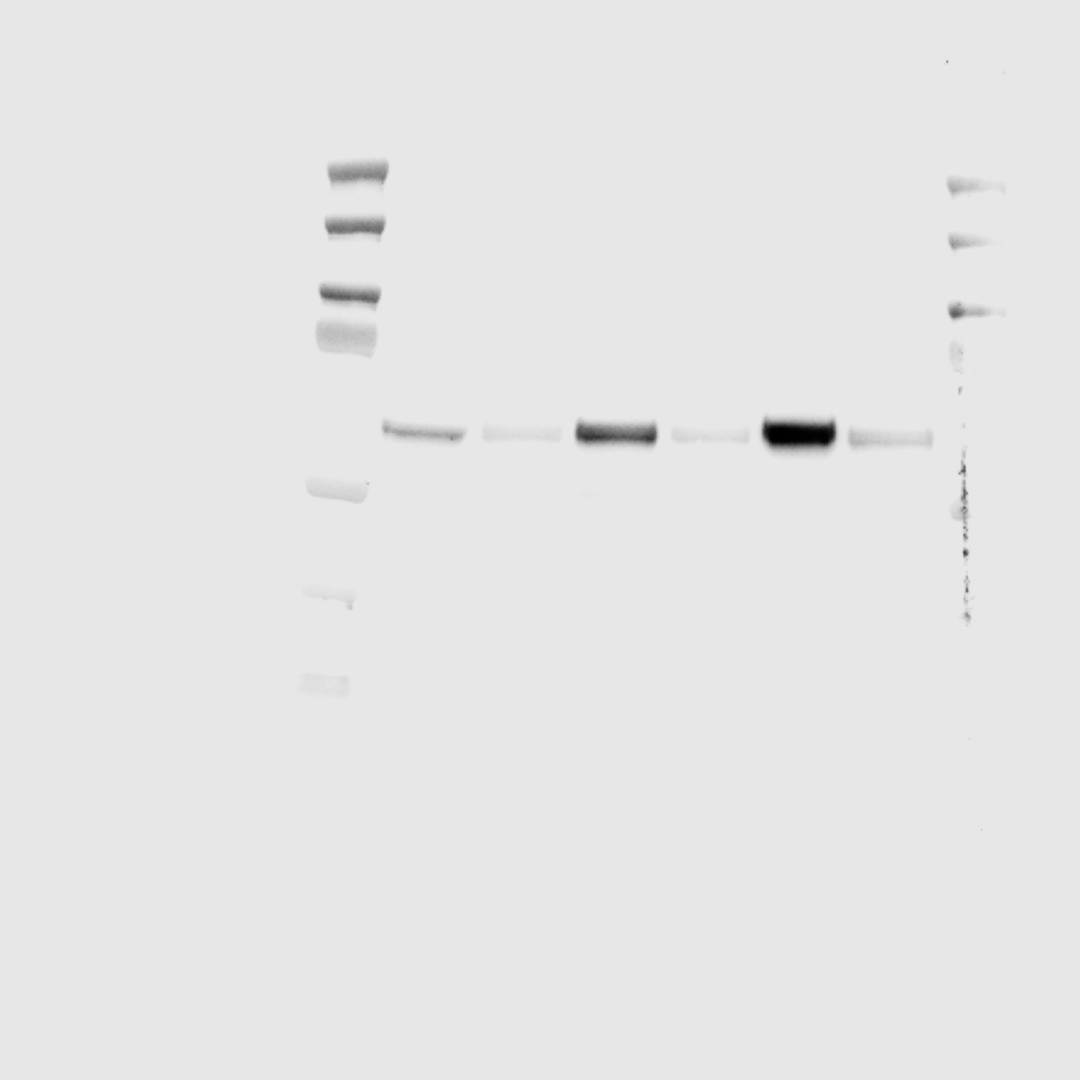
**

ii) B-Actin

**
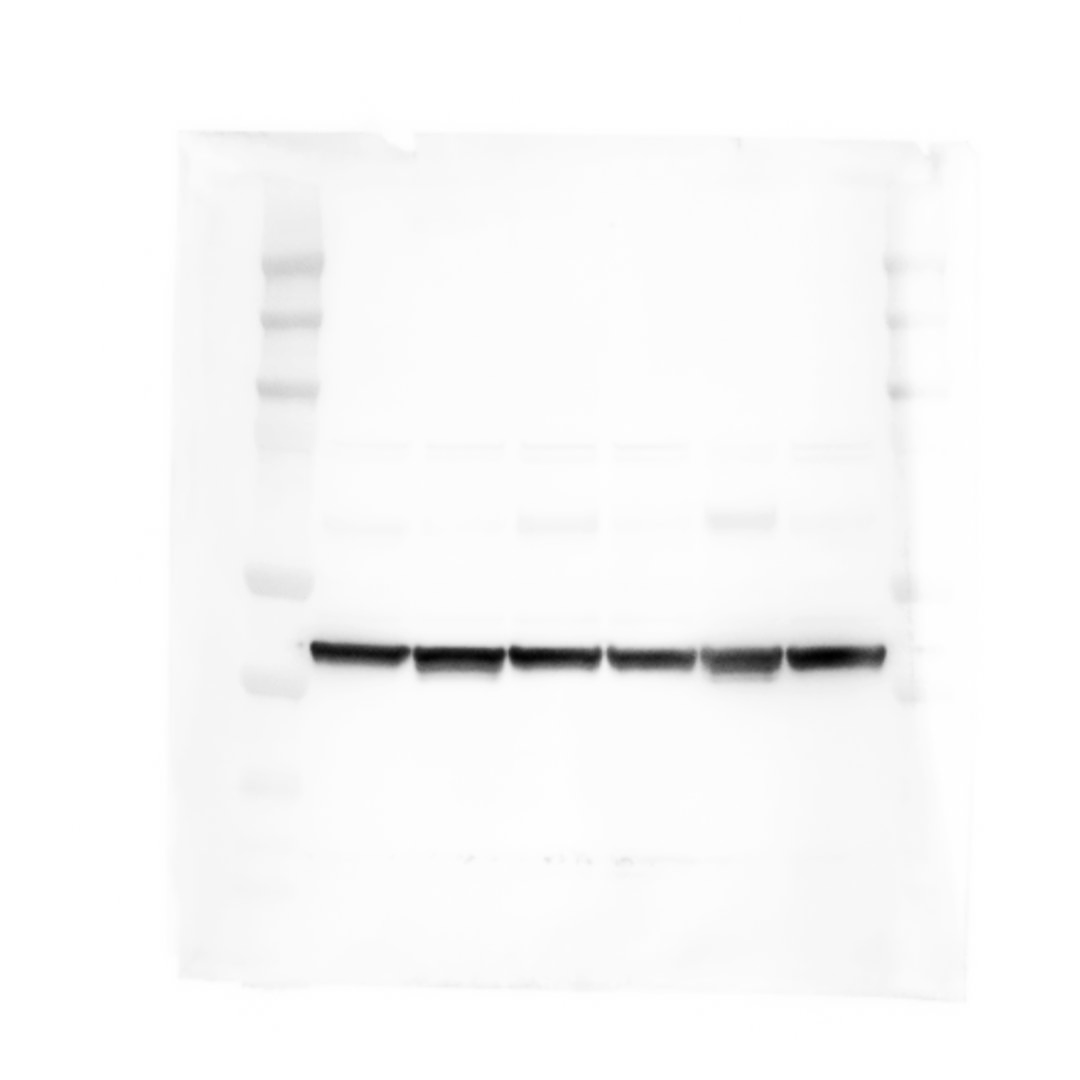


Supp. 1 K**i) B-Actin


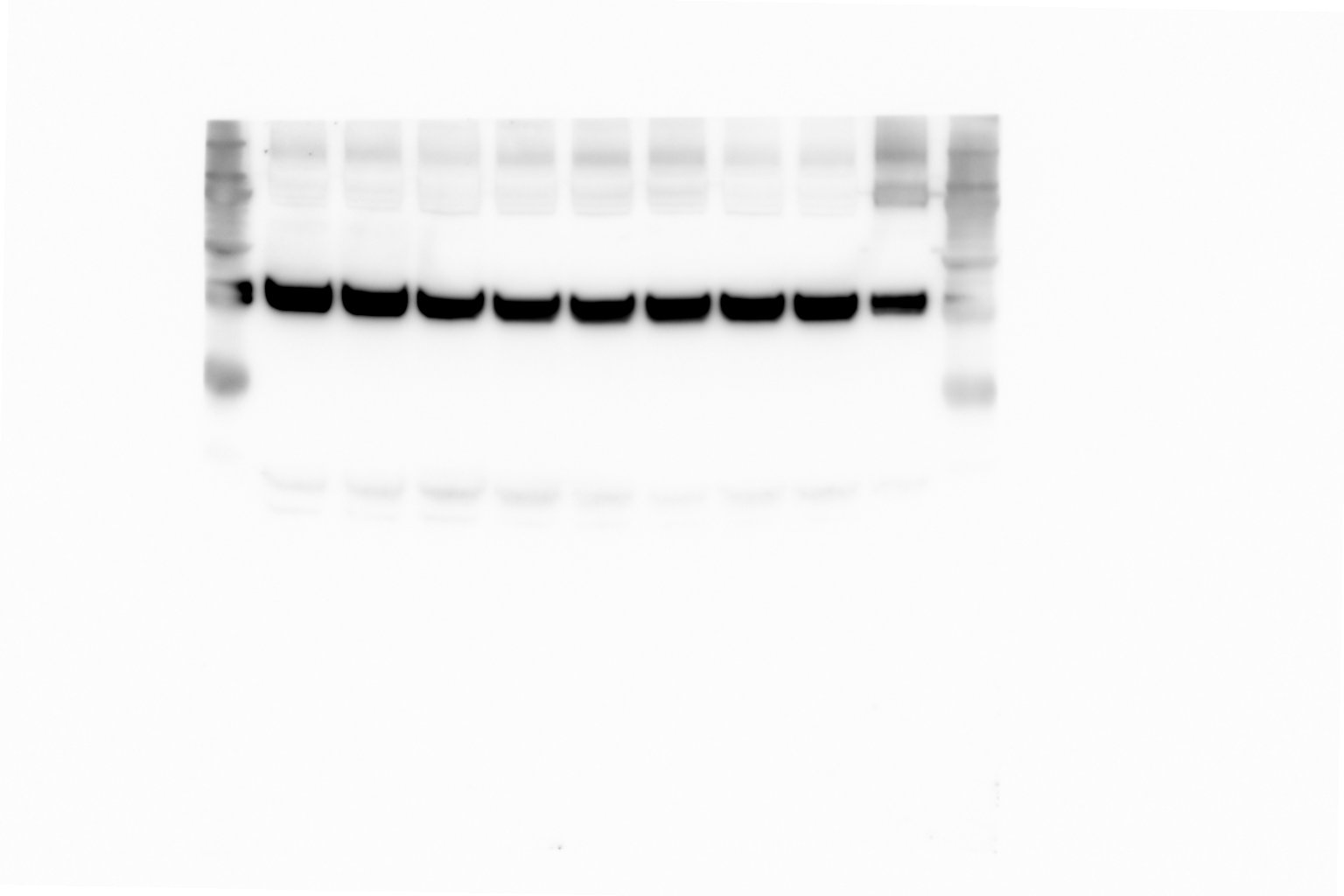


ii) pSynuclein


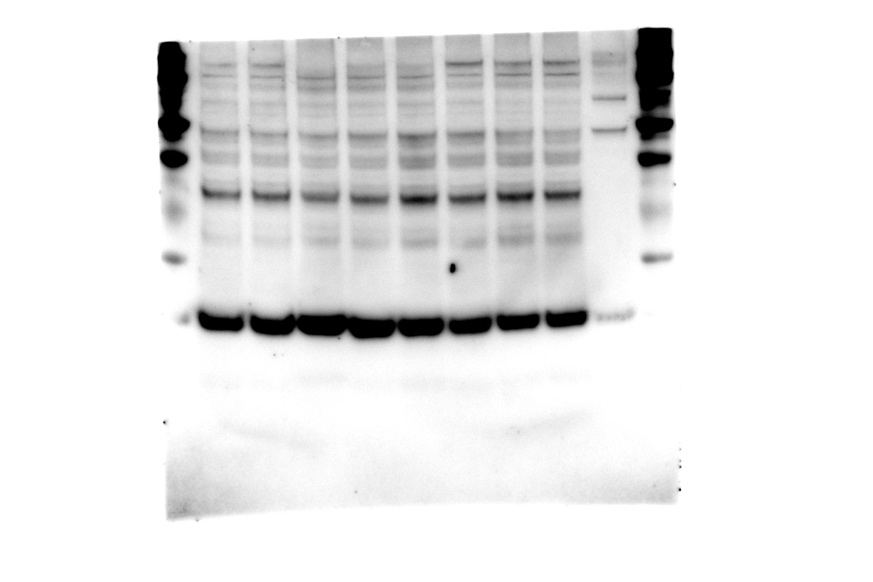


**Supp. 1 L**

**(Left Tx soluble)**i) B-Actin


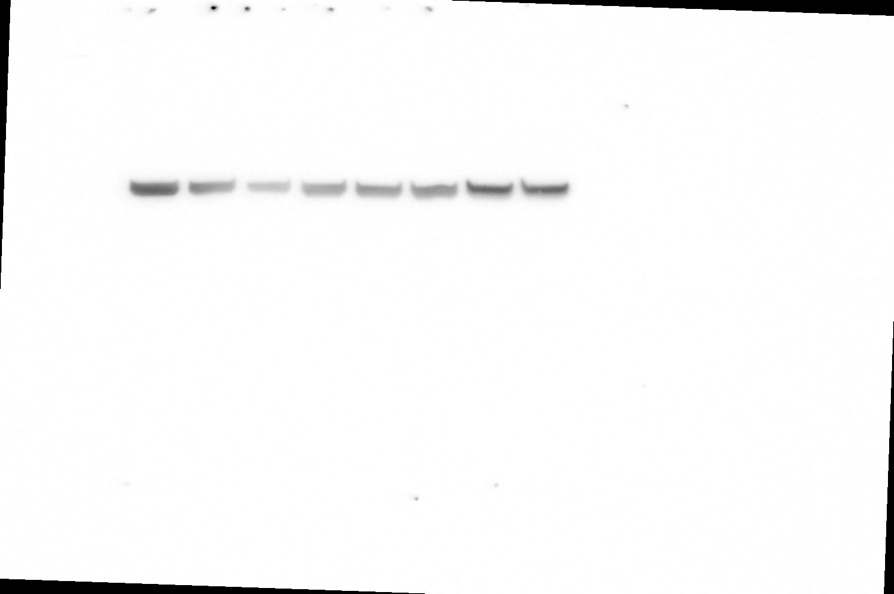


ii) SNCA


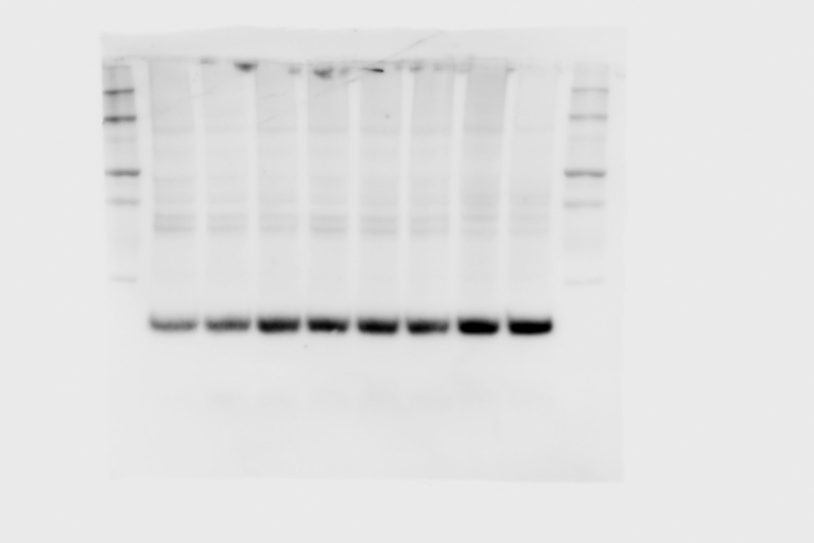


**(Right SDS/Tx insoluble)**

i) SNCA


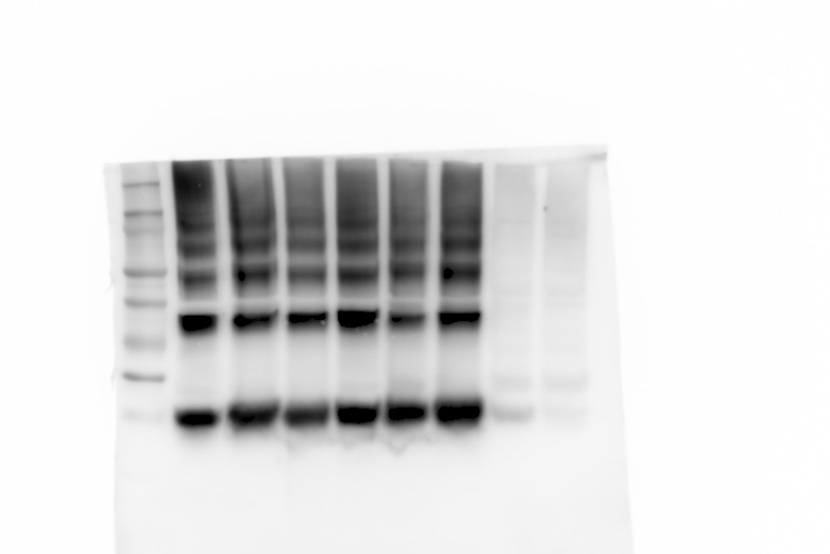


**Supplementary Figure 2**

**Supp. 2i**

i ) EMC4

**
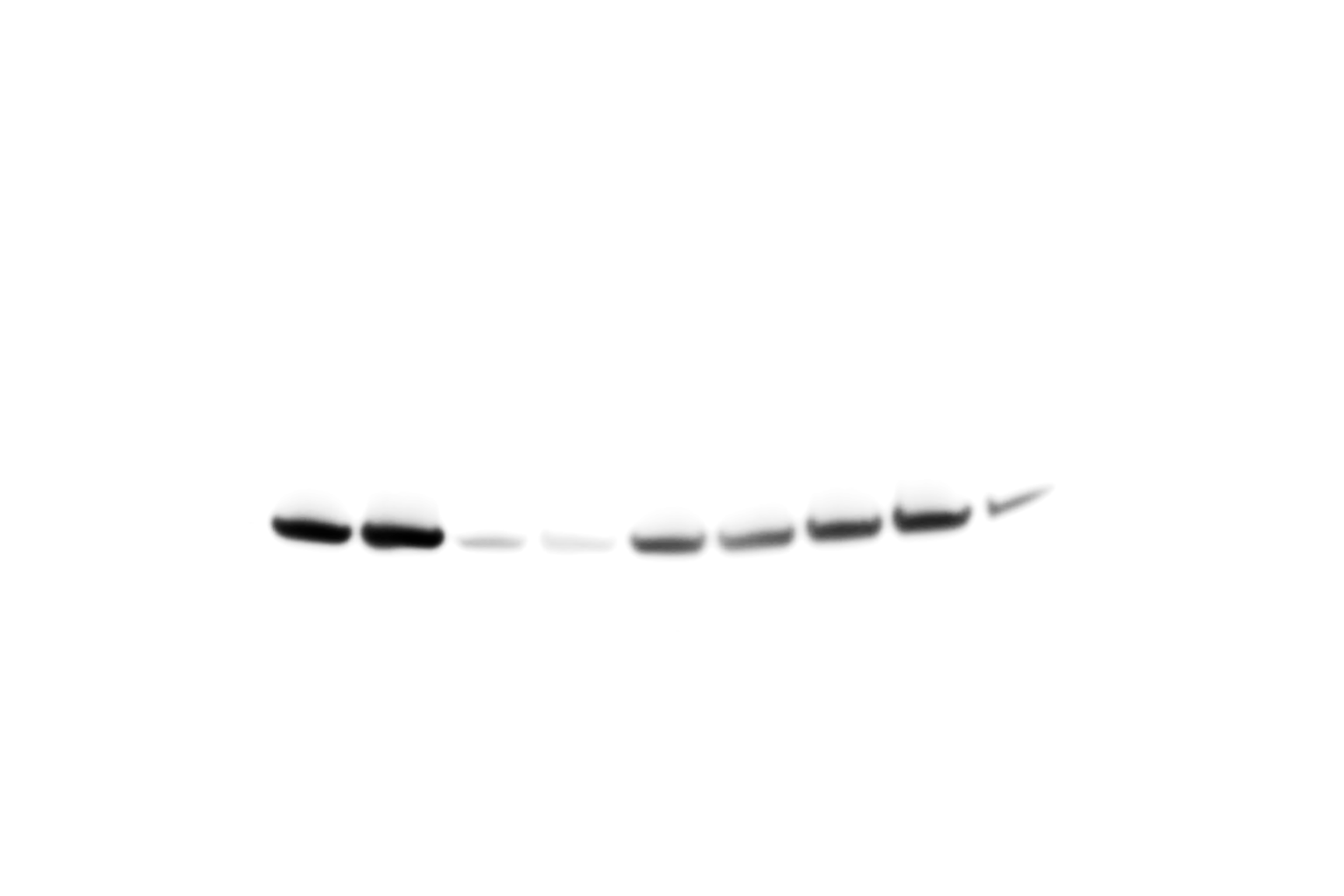
**

ii) B-Actin **
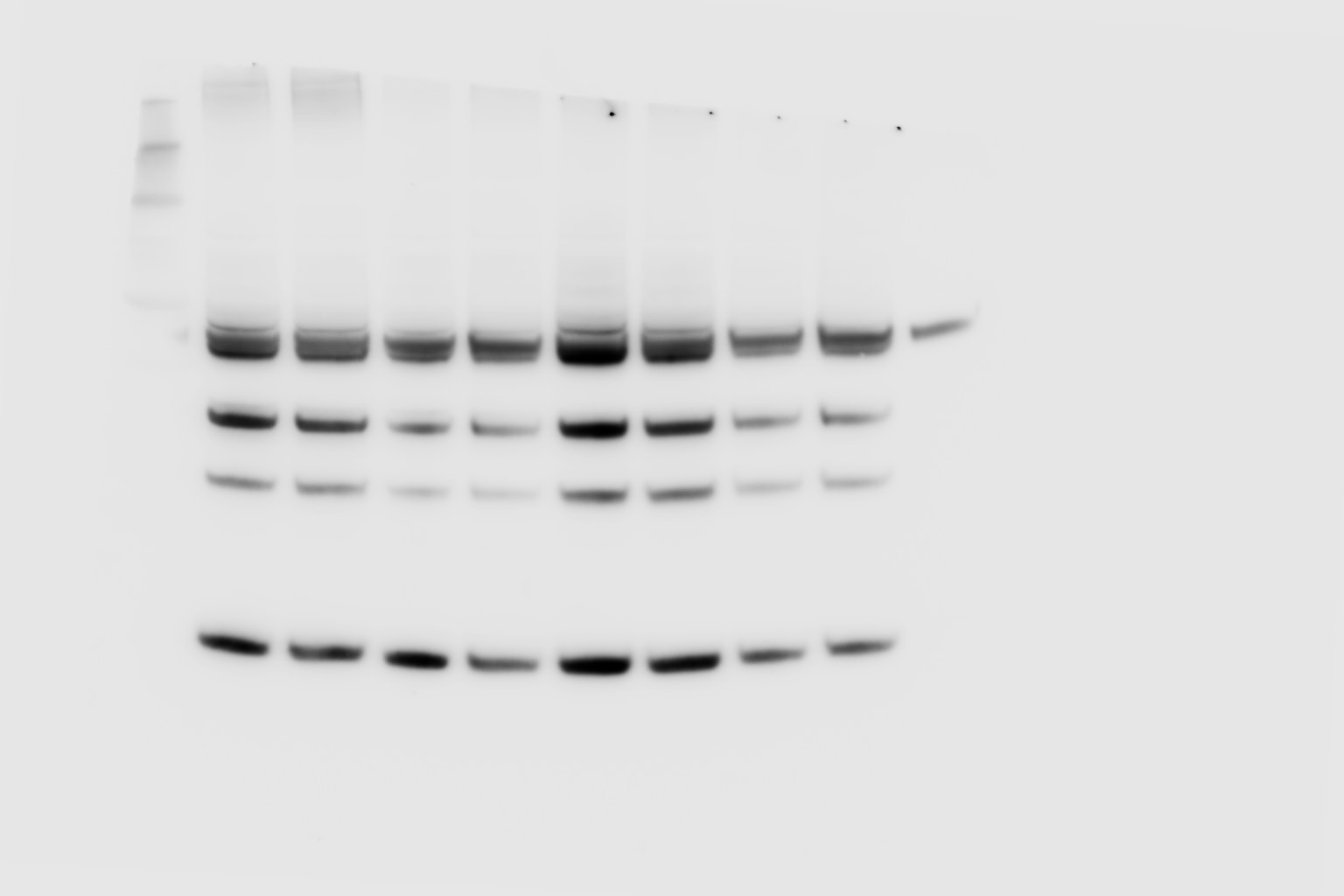
**

**Supp. 2k**i) OXR1 **
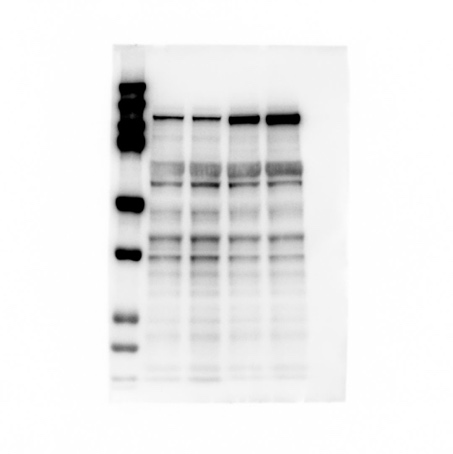
**

ii) B-Actin

**
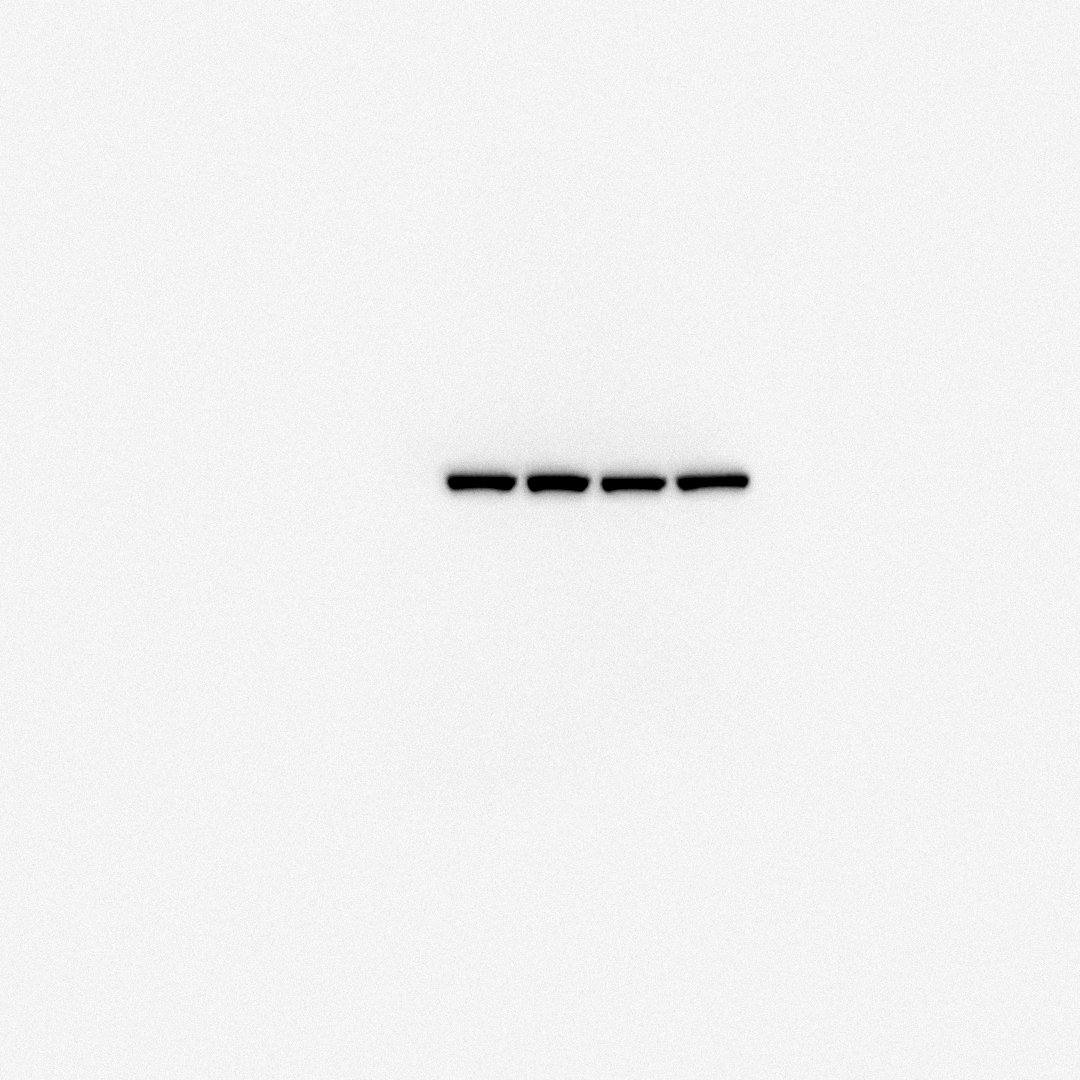
**

**Supplementary Figure 8**

**Supp. 8b**

i) α-Synuclein

**
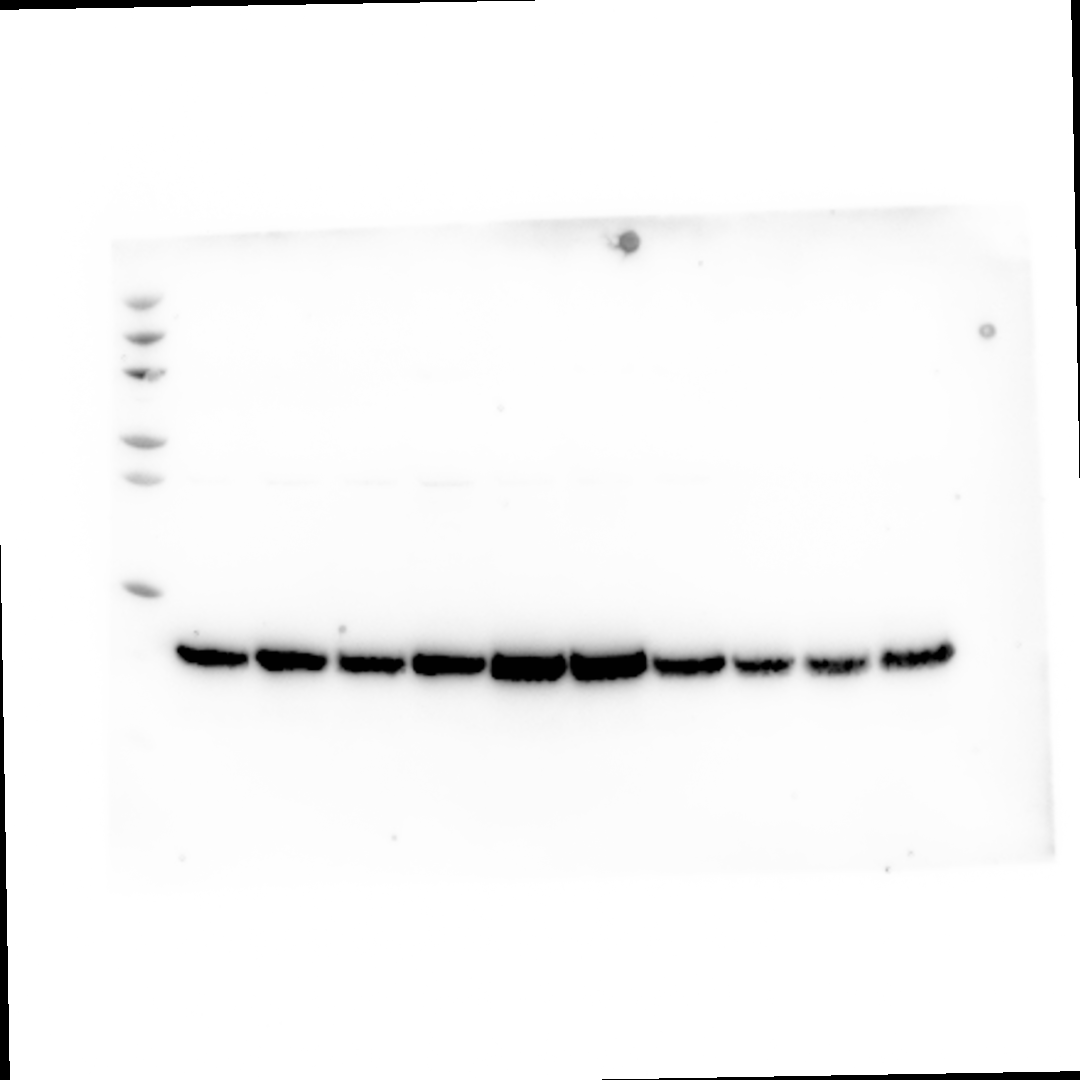
**

ii) OXR1

**
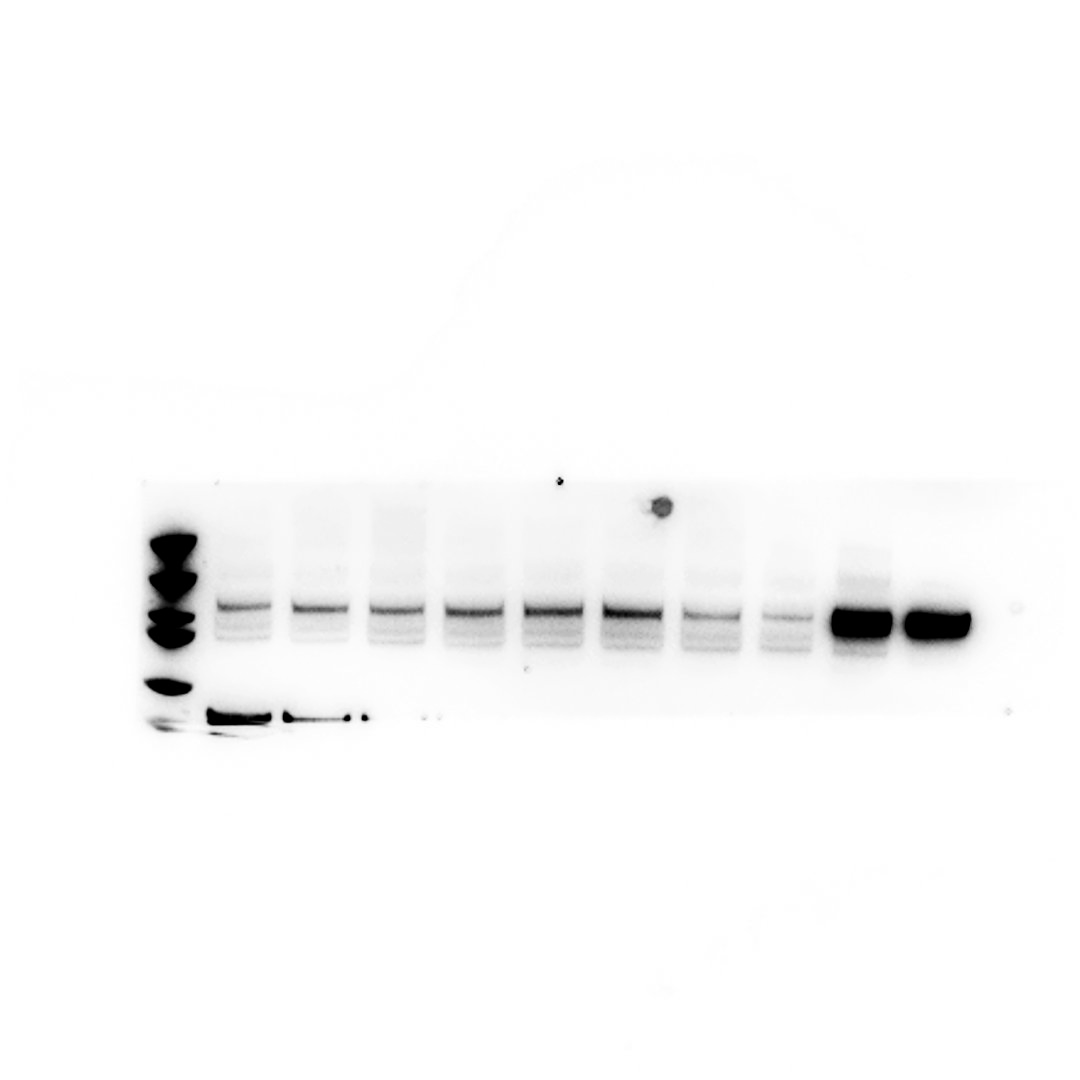
**

iii) B-Actin

**
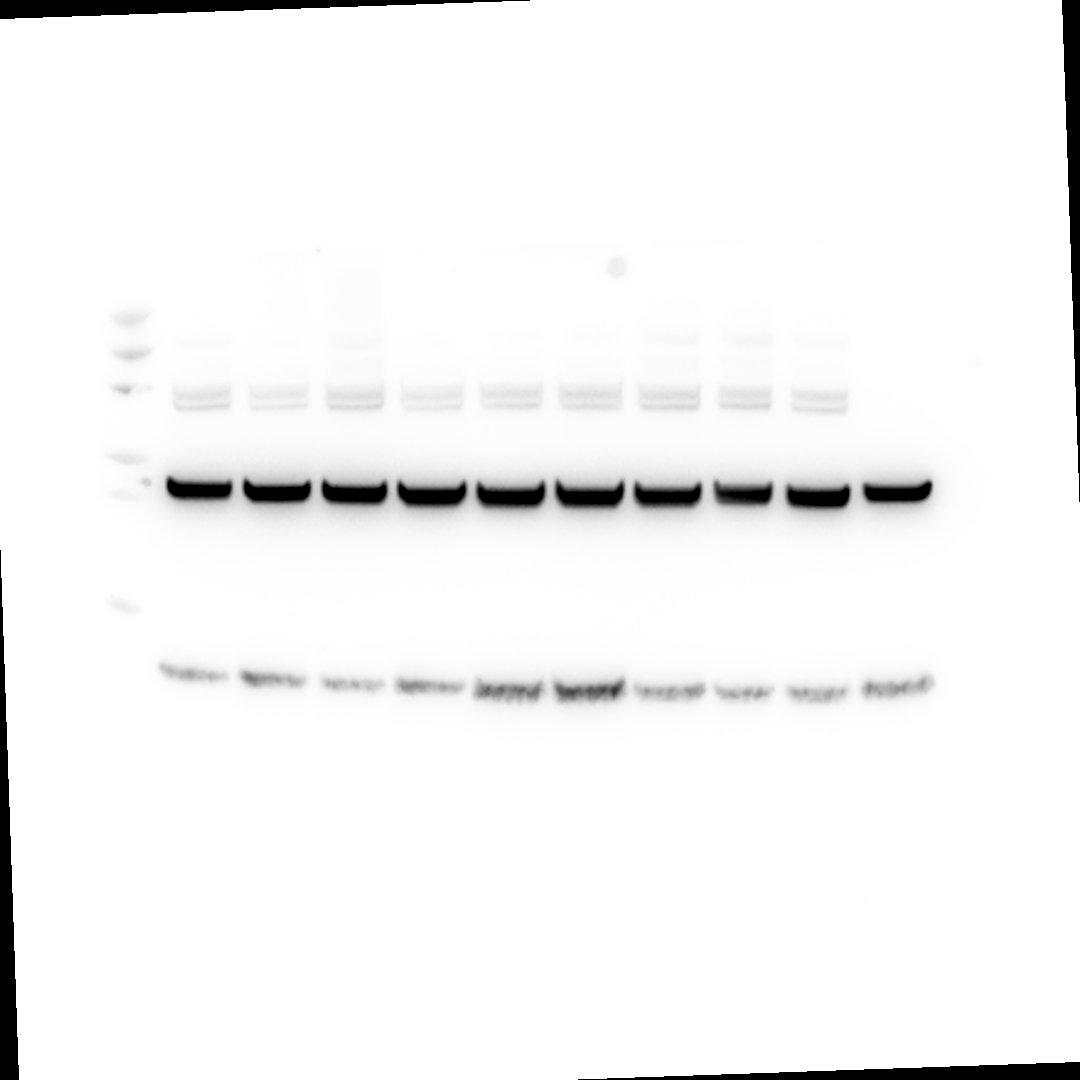
**
